# Synthesis and Evaluation of Phenyltriazole-Deoxynojirimycin Hybrids as Potent α-Glucosidase Inhibitors

**DOI:** 10.3390/molecules29215062

**Published:** 2024-10-26

**Authors:** Lin Wang, Wei Luo, Yonghong Zhao, Xinling Guo, Xiangru Bai, Leilei Guo, Nailiang Zhu

**Affiliations:** 1 School of Pharmacy, Xinyang Agriculture and Forestry University, Xinyang 464000, China; hong104071@163.com (L.W.); xynllw@163.com (W.L.); gxl15637681099@126.com (X.G.); baimaster@126.com (X.B.); gllym216@163.com (L.G.); 2 Nanjing Institute for Food and Drug Control, Nanjing 211198, China; zhaoyonghong1307@163.com

**Keywords:** 1-deoxynojirimycn, α-glucosidase, docking simulation, competitive inhibitor, cytotoxicity

## Abstract

1-deoxynojirimycin (DNJ) is a well-known α-glucosidase inhibitor. A series of phenyltriazole-deoxynojirimycin hybrids containing C_4_ and C_6_ (4 and 6 methylenes, respectively) linkers were synthesized. These novel compounds were assessed for preliminary glucosidase inhibition and cytotoxicity tests in vitro. Among them, compounds **12**–**14** and **16**–**20** (IC_50_: 105 ± 9–11 ± 1 μM) were more active than deoxynojirimycin (DNJ, IC_50_ = 155 ± 15 μM). The kinetics of enzyme inhibition measured by using Lineweaver–Burk plots indicated that compounds **18** and **19** were competitive inhibitors. In addition, a molecular docking study of α-glucosidase revealed that the interaction modes and the orientations of compound **18** and DNJ were clearly different. Furthermore, in tissue culture, HL60 cell compounds showed no cytotoxicity at low concentrations. When the concentration reached 50 µM, only compound **20** exhibited cytotoxicity. The structure–activity relationships exhibit that the length of the linker and the nature of 4-position substituents on the phenyl have a significant effect on the inhibitory potency of glucosidases and cytotoxicity.

## 1. Introduction

**Figure 1:**
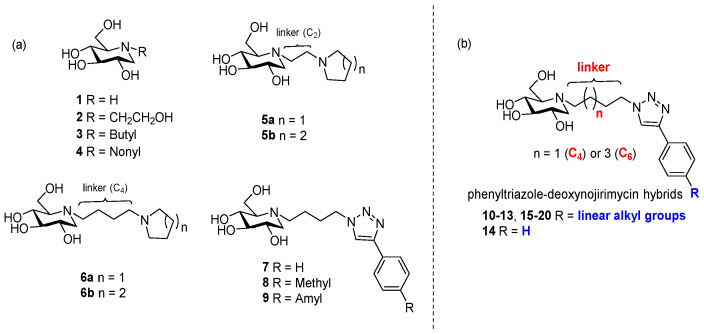
(**a**) Known potent glycosidase inhibitors derived from DNJ and (**b**) general structure of the library of phenyltriazole-deoxynojirimycin hybrids in this work.

Glucosidases are important enzymes which catalyze the hydrolysis of glycosidic bonds. They are found in all organisms and are involved in many biological processes, from the catabolism of carbohydrates to the biosynthesis of *O*- and *N*-glycoproteins and glycolipids [1,2,3]. Inhibitors of α-glucosidase can be used as oral antidiabetic drugs which suppress postprandial blood glucose levels by inhibiting the hydrolysis of disaccharides in the brush border membrane [4,5]. Among these drugs, voglibose [6], acarbose [7], and miglitol **2** [8] (Figure 1a) have been used for the treatment of Type 2 diabetes in the clinical setting. Miglitol was designed from the natural product DNJ **1** (Figure 1a). Resembling *D*-glucose in structure as well as mimicking an oxocarbeniumion-like transition state, DNJ exhibits good α-glucosidase inhibitory activity in a competitive manner, whereas its efficacy in vivo is only moderate [9,10].

The Cu-catalyzed azide-alkyne cycloaddition (CuAAC) is one of the powerful tools for the generation of glycosidase inhibitors based on DNJ. Gouin et al. [11] developed a click procedure to tether hydrophobic substituents to the *N*-propyl-DNJ core. All compounds were evaluated for inhibitory potency of commercially available glucosidases. Several studies have reported the synthesis of multivalent inhibitors using this method, presented on various scaffolds, such as fullerene [12], cyclodextrin [13], calixarene [14], peptoid [15] and self-assembled scaffolds [16]. Multivalent iminosugars allowed a significant enhancement in inhibitory activity and a remarkable selectivity against glycosidases [17]. The multivalent interactions between inhibitors and glycosidases showed a feature called the “multivalent effect” or “glycoside cluster effect” [18].

Modifying a known α-glucosidase inhibitor, especially a natural product, is a viable strategy to obtain more selective and stronger inhibitors. Generally, there are two main strategies for modification of DNJ: (a) introduction of different alkyl groups on the nitrogen atom or (b) alterations of the ring hydroxyl residues [19]. An important structural feature shared by DNJ with therapeutic significance is N-alkylation. Two *N*-alkylated DNJ derivatives, *N*-hydroxyethyl-DNJ (miglitol) and *N*-butyl-DNJ (miglustat) **3** [20], have been approved for the treatment of type II diabetes and Gaucher disease, respectively. Furthermore, it has been demonstrated that a lengthening of the alkyl chain linking DNJ provides better selectivity and potency towards α-glucosidase (e.g., **4**) [21].

The synthetic modification of DNJ is expected to tune and optimize its properties. In our previous work, we found compounds **6a** and **6b** (Figure 1a), which possessed a longer alkyl chain (C_4_), exhibited better inhibition potency against α-glucosidase compared to **5a** and **5b** carrying a C_2_ chain. Nevertheless, they were weaker α-glucosidase inhibitors than DNJ, being inactive against β-glucosidase [22]. In the subsequent structural modification of **6a** and **6b**, compound **9** (IC_50_ = 0.063 ± 0.006 mM) bearing amyl on the 4-position of the phenyl showed increased inhibitory potency against α-glucosidase compared to compounds **7** (IC_50_ = 0.551 ± 0.023 mM), **8** (IC_50_ = 0.324 ± 0.065 mM) and DNJ (IC_50_ = 0.155 ± 0.015 mM), which suggested that the length of alkyl chain on the 4-position of the phenyl had a great effect on inhibition against α-glucosidase [23].

Previous studies have indicated that DNJ with chain lengths above C_8_ exhibited a chain-length-dependent increase in cytotoxicity [24]. With this rationale in mind, and considering that three leads **6a**, **6b** and **9** showed potent α-glucosidase inhibitory activities. We here describe a series of new compounds characterized by the presence of the DNJ moiety linked to the phenyl with linkers of variable length (4 and 6 methylenes, respectively) via a triazole and bearing straight linear alkyl chain of various lengths on the 4-position of the phenyl (Figure 1b). The activity of these new hybrids was evaluated towards commercial α- and β-glucosidases. Kinetic analyses and the molecular docking study were further performed to evaluate their potency and mechanism of action. Finally, the cell toxicity of these compounds was analysed by the CCK-8 assay.

## 2. Results and Discussion

### 2.1. Synthesis of Phenyltriazole-Deoxynojirimycin Hybrids

**Scheme 1:**
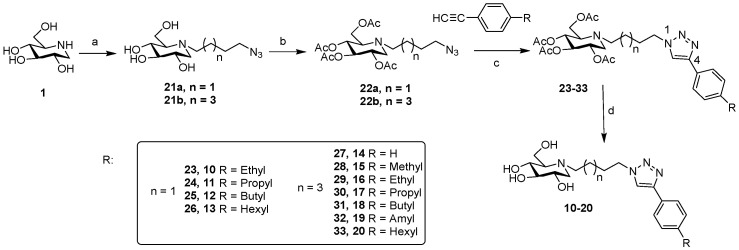
Synthesis of the target 1,4-disubstituted cycloadducts **10–20**. Reaction conditions: (a) K_2_CO_3_, DMF, 80 °C, 4 h; (b) Ac_2_O, Py, r.t., 12 h (over two steps, 86% (**22a**), 83% (**22b**); (c) CuSO_4_·5H_2_O, Na ascorbate, DMF/H_2_O (2:1), r.t., 6 h, 81–92% (**23–33**); (d) MeOH, NaOMe, r.t., 91–96% (**10–20**).

We synthesized the targeted molecules **10–20** (Scheme 1) using a procedure that we implemented for the synthesis of **7**, **8** and **9**. N-alkylation of unprotected DNJ **1** through the nucleophilic displacement of 1-azido-4-iodobutane or 1-azido-6-iodohexyl in DMF in the presence of K_2_CO_3_ at 80 °C afforded **21**, which were followed by peracetylation to provide the click precursors **22** in a mixture of pyridine and acetic anhydride at room temperature.

Compounds **23–33** were obtained between **22** and substituted phenylacetylenes through the copper-catalyzed azide-alkyne cycloaddition (CuAAC). **23–33** were obtained in good (81%) to excellent (92%) yields independently of the chain length of the coupling partners **22a** and **22b**. The structures of **23–33** were confirmed by ^1^H and ^13^C NMR spectroscopy, which showed the presence of a single 1,4-disubstituted regioisomer. Hydrogen chemical shifts of the triazole hydrogen (δ_H5_) was ∼7.76 ppm. The large and positive Δ (δ_C4_–δ_C5_) values (∼28 ppm) were observed by ^13^C NMR spectroscopy in agreement with earlier findings [25,26,27]. Finally, *O*-deacetylation with NaOMe in methanol at room temperature afforded the deprotected **10–20** in near quantitative yield. All compounds (except **15**) were soluble in water. Their structures were confirmed by ^1^H NMR spectroscopy, which displayed the absence of singlets for the COCH_3_ groups, which were further substantiated by HR-MS analysis ([M + H]^+^ for **10–20**).

### 2.2. Glycosidase Inhibitory Activity

Compounds **10–20** (except **15** for solubility reasons) were evaluated by in vitro α-glucosidase (yeast), β-glucosidase (almonds) inhibition studies. The corresponding IC_50_ values of compounds **10–20** were summarized in Table 1. Inhibition data for **7–9** and DNJ had also been included for comparative purposes.

It was first observed for a remarkable correlation between the length of the linker (C_4_ and C_6_) and the inhibition values, with a trend correlating higher inhibition with increased linker. In particular, compounds exhibited higher and more selective inhibitory effects on α-glucosidase. With the exception of analogues **7**, **8**, **10** and **11**, all compounds showed increased inhibitory activities against α-glucosidase compared to the parent DNJ. The inhibition profile was notably different with respect to β-glucosidase. Only compounds equipped with a C_6_ linker displayed more potent inhibition of β-glucosidase than DNJ. These results were in accordance with previous reports [28,29]. The affinity of *N*-alkyl-1-deoxynojirimycin derivatives for α- and β-glucosidase may be explained in terms of the enzyme active site having a lipophilic pocket [30,31].

On the other hand, the straight linear length on the 4-position of the phenyl also had a great effect on inhibition against α- and β-glucosidase. Amongst **7–13**, the simple extension of the alkyl chain by only one CH_2_ (from butyl to amyl) was sufficient to promote a 1.7-fold increase in α-glucosidase inhibitory activity. It was, however, interesting to note that increasing the alkyl chain length had little effect on β-glucosidase activity. For compounds **14–20**, no dramatic effect on α- and β-glucosidase was seen on a further increase in chain length from propyl to hexyl or butyl to hexyl, respectively. These results indicated that straight linear length on the 4-position of the phenyl only partially determined the accessibility of the compounds to the active site of the enzymes [29]. It was noteworthy to mention that **17–20** displayed more potent inhibition of α-glucosidase than β-glucosidase.

### 2.3. Inhibition Kinetics of α-Glucosidase

**Figure 2:**
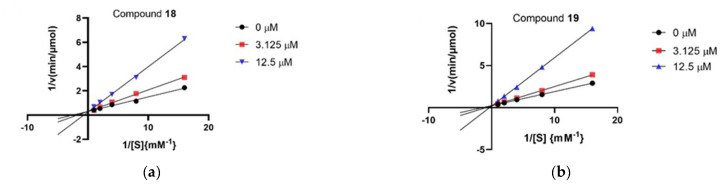
Double-reciprocal plots of the inhibition kinetics of yeast α-glucosidase by compounds **18** (**a**), **19** (**b**). Substrate concentration: 0.0625, 0.125, 0.25, 0.5, 1 mM; inhibitor concentration: 0 µM (control, ●), 3.125 µM (■), 12.5 µM (▲).

In order to gain further insight into how these hybrids interact with α-glucosidase, the mode of inhibition for compounds **18** and **19** as representative examples was analyzed using the Lineweaver–Burk plot (Figure 2). The double reciprocal plots of **18** and **19** showed straight lines with the same *V*_max_, suggesting that both were competitive α-glucosidase inhibitors. The inhibition constants (*K*_i_) were 3.8 µM for compound **18** and 3.7 µM for compound **19**.

### 2.4. Molecular Docking Studies

Molecular docking studies were performed to gain further insights into the binding mode of these DNJ analogues. Compound **18**, as a representative example, was chosen for the docking simulations. The parent compound DNJ was also studied for comparison. Since the X-ray crystal structure of a-glucosidase from Saccharomyces cerevisiae (MAL12, P53341) is currently unavailable, the homology modeling provided by SWISS-MODEL Repository, with the model quality estimation performed, was used as the receptor. From the docking studies, the binding affinity of compound **18** was predicted to be −8.6 kcal/mol, greater than the parent DNJ (−5.8 Kcal/mol). This indicated that compound **18** had stronger binding with α-glucosidase, which was consistent with the results observed in the enzyme inhibitory essays (Table 2).

**Figure 3:**
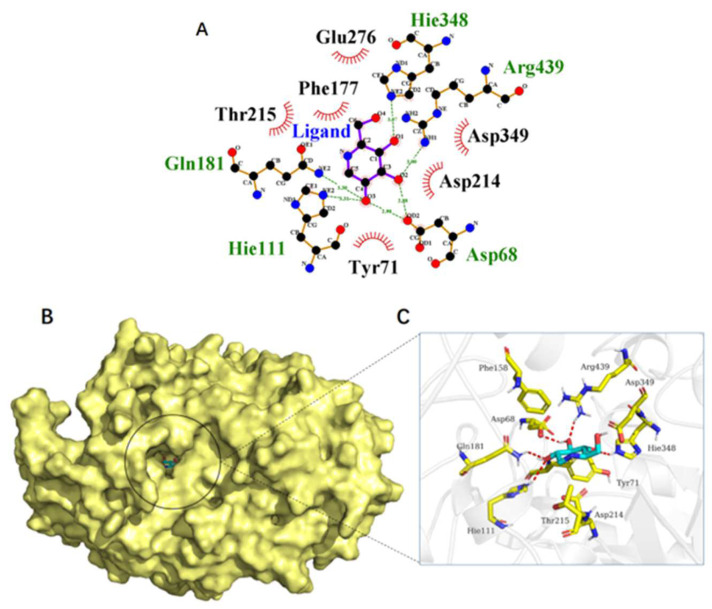
Molecular docking simulation of MAL12 and DNJ. (**A**) The 2D binding mode of MAL12 and DNJ. (**B**) The binding model of DNJ on the molecular surface of MAL12. DNJ is colored in cyan, and the molecular surface of MAL12 is colored in pale yellow. (**C**) The 3D binding mode of MAL12 and DNJ. DNJ is colored in cyan, the surrounding residues in the binding pockets are colored in yellow, and the backbone of the receptor is depicted as white cartoons with transparency.

**Figure 4:**
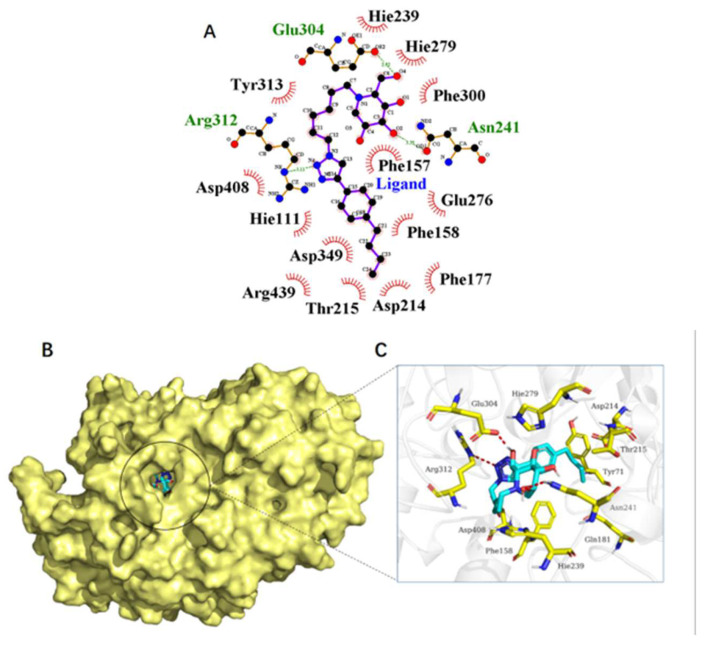
Molecular docking simulation of MAL12 and compound **18**: (**A**) The 2D binding mode of MAL12 and compound **18**. (**B**) The binding model of compound **18** on the molecular surface of MAL12. Compound **18** is colored in cyan, and the molecular surface of MAL12 is colored in pale yellow. (**C**) The 3D binding mode of MAL12 and compound **18**. Compound **18** is colored in cyan, the surrounding residues in the binding pockets are colored in yellow, and the backbone of the receptor is depicted as white cartoons with transparency.

Molecular docking analysis indicated that several types of interactions were formed between compound **18**, DNJ and enzyme upon binding, as seen in Figure 3 and Figure 4. DNJ was positioned to form hydrogen bonds with Hie348, Arg439, Asp68, Hie111 and Gln181. The DNJ moiety of compound **18** could form hydrogen bonds with Asn241 and Glu304. An additional hydrogen bond was established between the triazole moiety and Arg312. However, it was suggested that the alkyl chain containing a C_6_ spacer had additional hydrophobic and van der Waals interactions with hydrophilic residues (Tyr313, Phe300, et al.). Moreover, both interactions also exist between the butyl group on the 4-position of the phenyl hydrophilic residues (Phe158, Phe177, Thr215, et al.). Thus, the strengthening of the hydrophobic effect and van der Waals interactions seemed to compensate for the weakening of the hydrogen bond interactions, which could be an explanation for why compound **18** bonds to α-glucosidase more tightly than DNJ and resulted in stronger inhibitory activity. This was also consistent with the observations from the enzyme kinetic assay that compound **18** was a competitive inhibitor of α-glucosidase.

### 2.5. Cell Toxicity of Deoxynojirimycin-Triazole Hybrid Iminosugars

**Figure 5:**
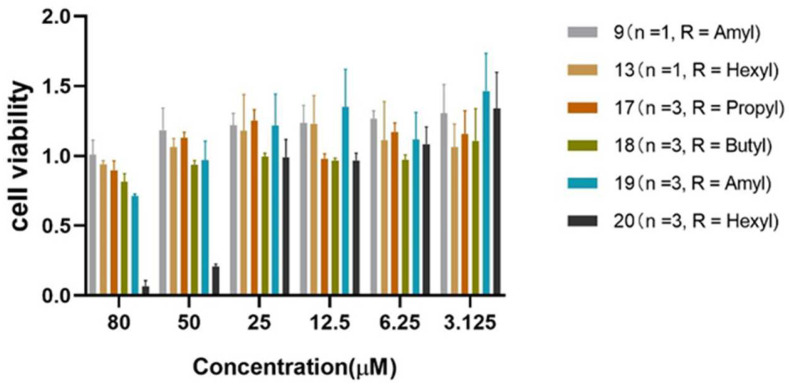
Effect of DNJ analogues on HL60 cell growth.

Compounds **9**, **13** and **17–20** with better inhibitory activity on α-glucosidase were selected to study the effect on cell viability and proliferation by CCK-8 assay (Figure 5). The cell survival rate gradually decreased with the increase in compound concentration. When viability was measured at 50 µM for these compounds, the viability of compound **19** was 96.97% compared with 20.89% for compound **20**. When the concentration was 80 µM, the cell survival rate was still above 80% except for compounds **19** (71.28%) and **20** (6.66%). With the increase in compound concentration, a lot of dead cells and more cell debris were observed, and the culture medium became muddy for **20**, which was not obviously observed for **9**, **13** and **17–19**.

## 3. Experimental Section

### 3.1. General Information

Two glycosidases, α-glucosidase (yeast) and β-glucosidase (almonds), and their corresponding substrates, p-nitrophenyl glycopyranosides, were purchased from Sigma Chemical Co., St. Louis, MO, USA. Other commercial reagents and solvents were used as received. Reactions were monitored by Thin Layer Chromatography (TLC) analyses using silica gel GF_254_ plates bearing a 0.25 mm layer. Visualization of compounds was detected by short-wave UV fluorescence (λ = 254 nm) and/or by dipping into 5% phosphomolybdic acid in EtOH. Column chromatography was carried out by silica gel (200–300 mesh). NMR spectra (^1^H and ^13^C) were recorded with Bruker AV III 500 MHz spectrometer using CDCl_3_ (**22–33**), D_2_O (**10–13**, **16–20**) or CD_3_OD (**14**, **15**) as solvents. High-resolution mass spectra (HRMS) were obtained by direct injection on a mass spectrometer (Thermo Scientific LTQ Orbitrap XL, Waltham, MA, USA) equipped with an electrospray ion source in positive mode (see Supplementary Materials).

### 3.2. General Procedure A—Peracetylation with ***21a*** or ***21b***

To a solution of DNJ (1.0 eq.), 1-azido-4-iodobutane (1.5 eq.) or 1-azido-6-iodohexyl (1.5 eq.) in DMF was added potassium carbonate (2.0 eq.). The reaction mixture was heated to 80 °C for 4 h. Most of the DMF was evaporated under reduced pressure. The crude product was dissolved in pyridine-acetic anhydride (1:1) and stirred for 12 h. The mixture was poured into saturated aqueous NaHCO_3_, and the aqueous phase was extracted with EtOAc. The combined organic layers were dried (Na_2_SO_4_), filtered and concentrated. The residue was purified by silica gel column chromatography (EtOAc/petroleum ether 1:6 to 1:2).

**(2R,3R,4R,5S)-2-(acetoxymethyl)-1-(4-azidobutyl)piperidine-3,4,5-triyl triacetate 22a**

Prepared according to procedure A. DNJ (1.0 g, 6.1 mmol), 1-azido-4-iodobutane (2.1 g, 9.3 mmol), potassium carbonate (2.5 g, 18 mmol), pyridine-acetic anhydride (1:1, 30 mL). Yield: 2.3 g, 5.4 mmol, 86%, a pale-yellow oil. ^1^H NMR (500 MHz, CDCl_3_) δ 5.15–5.02 (m, 2H, H-6), 4.96 (td, J = 9.9, 5.2 Hz, 1H, H-2), 4.17 (qd, J = 12.9, 2.7 Hz, 2H, H-3, H-4), 3.31 (m, 2H, H-10), 3.21 (dd, J = 11.5, 5.1 Hz, 1H, H-1a), 2.88–2.73 (m, 1H, H-7a), 2.64 (dd, J = 6.3, 2.7 Hz, 1H, H-5), 2.56 (m, 1H, H-7b), 2.36–2.25 (m, 1H, H-1b), 2.12–1.92 (4s, 12H, COCH_3_), 1.56 (m, 4H, H-8, H-9); ^13^C NMR (125 MHz, CDCl_3_) δ 170.82, 170.32, 170.04, 169.74 (4 × C=O), 74.57 (C-3), 69.43 (C-4), 69.29 (C-2), 61.74 (C-6), 59.46 (C-5), 52.68 (C-1), 51.14 (C-7), 50.94 (C-10), 26.44 (C-9), 22.45 (C-8), 20.84, 20.79, 20.72, 20.66 (4 × COCH_3_).

**(2R,3R,4R,5S)-2-(acetoxymethyl)-1-(6-azidohexyl)piperidine-3,4,5-triyl triacetate 22b**

Prepared according to procedure A. DNJ (1 g, 6.1 mmol), 1-azido-6-iodohexyl (2.2 g, 9.3 mmol), potassium carbonate (2.5 g, 18 mmol), pyridineyridine-acetic anhydride (1:1, 30 mL). Yield: 2.3 g, 5.1 mmol, 83%, a pale-yellow oil. ^1^H NMR (500 MHz, CDCl_3_) δ 5.06 (dt, J = 18.5, 9.3 Hz, 2H, H-6), 4.98–4.89 (m, 1H, H-2), 4.25–4.08 (m, 2H, H-3, H-4), 3.27 (t, J = 6.9 Hz, 2H, H-12), 3.20 (dd, J = 11.4, 5.1 Hz, 1H, H-1a), 2.75 (ddd, J = 13.8, 9.8, 6.1 Hz, 1H, H-7a), 2.64 (d, J = 9.0 Hz, 1H, H-5), 2.60–2.50 (m, 1H, H-7b), 2.32 (t, J = 10.9 Hz, 1H, H-1b), 2.06 (4s, 12H, 4 × COCH_3_), 1.65–1.53 (m, 2H, H-11), 1.53–1.22 (m, 6H, H-8, H-9. H-10). ^13^C NMR (125 MHz, CDCl_3_) δ 170.95, 170.41, 170.09, 169.79 (4 × C=O), 74.68 (C-3), 69.51 (C-4), 69.41 (C-2), 61.57 (C-6), 59.52 (C-5), 52.89 (C-1), 51.60 (C-7), 51.35 (C-12), 28.81 (C-11), 26.75 (C-8), 26.60 (C-9), 24.77 (C-10), 20.89, 20.85, 20.77, 20.71 (4 × CO*C*H_3_).

### 3.3. General Procedure B—CuAAC Reaction with ***22a*** or ***22b***

To a solution of **22a** or **22b** (1.0 eq.) and substituted phenylacetylenes (2 eq.) in DMF/H_2_O (2:1) was added Copper (II) sulfate (0.3 eq.) and sodium ascorbate (0.6 eq.). The mixture was stirred at room temperature for 6 h. Saturated NaHCO_3_ solution and EtOAc were added. The organic layer was dried over Na_2_SO_4_ and concentrated. The residue was purified by flash column chromatography using EtOAc:PE (2:1 → 1:4).

**(2R,3R,4R,5S)-2-(acetoxymethyl)-1-(4-(4-(4-ethylphenyl)-1H-1,2,3-triazol-1-yl)butyl)piperidine-3,4,5-triyl triacetate 23**

Prepared according to procedure B. Compound **22a** (200 mg, 0.47 mmol), 1-ethyl-4-ethynylbenzene (122 mg, 0.94 mmol), CuSO_4_·5H_2_O (34.96 mg, 0.14 mmol), sodium ascorbate (55.47 mg, 0.28 mmol), DMF/H_2_O (3 mL, 2:1). Yield: 210 mg, 0.38 mmol, 81%, white solid, *R*_f_ = 0.49 (1:2; PE:EtOAc). ^1^H NMR (500 MHz, CDCl_3_) δ 7.86–7.65 (m, 3H, ArH, CH- triazole), 7.39–7.10 (m, 2H, ArH), 5.14–5.00 (m, 2H, H-6), 4.94 (td, J = 9.9, 5.1 Hz, 1H, H-2), 4.42 (dq, J = 13.9, 6.8 Hz, 2H, H-10), 4.16 (qd, J = 12.9, 2.7 Hz, 2H, H-3, H-4), 3.17 (dd, J = 11.5, 5.0 Hz, 1H, H-1a), 2.84 (dt, J = 13.5, 7.9 Hz, 1H, H-7a), 2.68 (q, J = 7.6 Hz, 2H, H-13), 2.61 (dd, J = 6.5, 2.6 Hz, 1H, H-5), 2.53 (ddd, J = 13.2, 8.3, 4.8 Hz, 1H, H-7b), 2.23 (t, J = 10.9 Hz, 1H, H-1b), 2.09–1.97 (m, 12H, 4 × COCH_3_), 1.97–1.85 (m, 2H, H-9), 1.60–1.43 (m, 2H, H-8), 1.26 (t, 3H, H-14). ^13^C NMR (125 MHz, CDCl_3_) δ 170.83, 170.32, 170.13, 169.79 (4 × C=O), 147.99 (C-12), 144.40 (C_ar_), 128.36 (2 × CH_ar_), 128.04 (C_ar_), 125.73 (2 × CH_ar_), 119.15 (C-11), 74.52 (C-3), 69.42 (C-4), 69.25 (C-2), 62.00 (C-6), 59.39 (C-5), 52.52 (C-1), 50.58 (C-7), 49.95 (C-10), 28.69 (C-13), 27.82 (C-9), 22.50 (C-8), 20.87, 20.84, 20.76, 20.70 (4 × CO*C*H_3_), 15.56 (C-14).

**(2R,3R,4R,5S)-2-(acetoxymethyl)-1-(4-(4-(4-propylphenyl)-1H-1,2,3-triazol-1-yl)butyl)piperidine-3,4,5-triyl triacetate 24**

Prepared according to procedure B. Compound **22a** (200 mg, 0.47 mmol), 1-eth-1-ynyl-4-propylbenzene (135 mg, 0.94 mmol), CuSO_4_·5H_2_O (34.96 mg, 0.14 mmol), sodium ascorbate (55.47 mg, 0.28 mmol), DMF/H_2_O (3 mL, 2:1). Yield: 220 mg, 0.39 mmol, 82%, white solid, *R*_f_ = 0.51 (1:2; PE:EtOAc). ^1^H NMR (500 MHz, CDCl_3_) δ 7.89–7.68 (m, 3H, ArH, CH- triazole), 7.25 (dd, J = 8.1, 4.0 Hz, 2H, ArH), 5.16–5.02 (m, 2H, H-6), 4.96 (dd, J = 9.2, 4.8 Hz, 1H, H-2), 4.43 (td, J = 11.7, 7.0 Hz, 2H, H-10), 4.26–4.10 (m, 2H, H-3, H-4), 3.18 (dt, J = 11.2, 4.5 Hz, 1H, H-1a), 2.90–2.80 (m, 1H, H-7a), 2.68–2.58 (m, 3H, H-5, H-13), 2.59–2.48 (m, 1H, H-7b), 2.25 (td, J = 11.4, 3.3 Hz, 1H, H-1b), 2.11–1.86 (m, 14H, 4 × COCH_3_, H-9), 1.68 (ddd, J = 15.1, 7.5, 4.1 Hz, 2H, H-14), 1.58–1.43 (m, 2H, H-8), 0.97 (td, J = 7.3, 4.0 Hz, 3H, H-15). ^13^C NMR (125 MHz, CDCl_3_) δ 170.83, 170.32, 170.13, 169.80 (4 × C=O), 147.97 (C-12), 142.83 (C_ar_), 128.96 (2 × CH_ar_), 128.01 (C_ar_), 125.61 (2 × CH_ar_), 119.19 (C-11), 74.51 (C-3), 69.41 (C-4), 69.25 (C-2), 61.95 (C-6), 59.37 (C-5), 52.49 (C-1), 50.57 (C-7), 49.93 (C-10), 37.81 (C-13), 27.80 (C-9), 24.50 (C-14), 22.44 (C-8), 20.86, 20.82, 20.74, 20.69 (4 × CO*C*H_3_), 13.81 (C-15).

**(2R,3R,4R,5S)-2-(acetoxymethyl)-1-(4-(4-(4-butylphenyl)-1H-1,2,3-triazol-1-yl)butyl)piperidine-3,4,5-triyl triacetate 25**

Prepared according to procedure B. Compound **22a** (200 mg, 0.47 mmol), 1-butyl-4-eth-1-ynylbenzene (148 mg, 0.94 mmol), CuSO_4_·5H_2_O (34.96 mg, 0.14 mmol), sodium ascorbate (55.47 mg, 0.28 mmol), DMF/H_2_O (3 mL, 2:1). Yield: 220 mg, 0.38 mmol, 80%, white solid, *R*_f_ = 0.53 (1:2; PE:EtOAc). ^1^H NMR (500 MHz, CDCl_3_) δ 7.85–7.67 (m, 3H, ArH, CH- triazole), 7.23 (d, J = 8.0 Hz, 2, ArH), 5.04 (dt, J = 15.5, 6.1 Hz, 2H, H-6), 4.94 (d, J = 4.4 Hz, 1H, H-2), 4.53–4.35 (m, 2H, H-10), 4.16 (qd, J = 12.9, 2.4 Hz, 2H, H-3, H-4), 3.16 (dd, J = 11.3, 4.7 Hz, 1H, H-1a), 2.92–2.77 (m, 1H, H-7a), 2.62 (m, 3H, H-5, H-13), 2.57–2.46 (m, 1H, H-7b), 2.23 (t, J = 10.9 Hz, 1H, H-1b), 2.09–1.82 (m, 14H, 4 × COCH_3_, H-9), 1.71–1.57 (m, 2H, H-14), 1.54–1.41 (m, 2H, H-8), 1.41–1.31 (m, 2H, H-15), 0.93 (t, J = 7.3 Hz, 3H, H-16). ^13^C NMR (125 MHz, CDCl_3_) δ 170.80, 170.29, 170.10, 169.77 (4 × C=O), 147.94 (C-12), 143.04 (C_ar_), 128.89 (2 × CH_ar_), 127.96 (C_ar_), 125.61 (2 × CH_ar_), 119.20 (C-11), 74.50 (C-3), 69.41 (C-4), 69.24 (C-2), 61.95 (C-6), 59.37 (C-5), 52.48 (C-1), 50.57 (C-7), 49.92 (C-10), 35.42 (C-13), 33.55 (C-14), 27.78 (C-9), 22.43 (C-15), 22.31 (C-8), 20.83, 20.80, 20.72, 20.66 (4 × CO*C*H_3_), 13.96 (C-16).

**(2R,3R,4R,5S)-2-(acetoxymethyl)-1-(4-(4-(4-hexylphenyl)-1H-1,2,3-triazol-1-yl)butyl)piperidine-3,4,5-triyl triacetate 26**

Prepared according to procedure B. Compound **22a** (200 mg, 0.47 mmol), 1-ethynyl-4-hexylbenzene (174 mg, 0.94 mmol), CuSO_4_·5H_2_O (34.96 mg, 0.14 mmol), sodium ascorbate (55.47 mg, 0.28 mmol), DMF/H_2_O (3 mL, 2:1). Yield: 260 mg, 0.42 mmol, 90%, white solid, *R*_f_ = 0.55 (1:2; PE:EtOAc). ^1^H NMR (500 MHz, CDCl_3_) δ 7.82–7.69 (m, 3H, ArH, CH- triazole), 7.24 (d, J = 8.1 Hz, 2H, ArH), 5.11–5.00 (m, 2H, H-6), 4.94 (td, J = 9.9, 5.1 Hz, 1H, H-2), 4.54–4.33 (m, 2H, H-10), 4.16 (qd, J = 12.9, 2.7 Hz, 2H, H-3, H-4), 3.16 (dd, J = 11.5, 5.0 Hz, 1H, H-1a), 2.83 (m, 1H, H-7a), 2.71–2.56 (m, 3H, H-5, H-13), 2.52 (m, 1H, H-7b), 2.34–2.16 (m, 1H, H-1b), 2.07–1.87 (m, 14H, 4 × COCH_3_, H-9), 1.62 (dt, J = 15.3, 7.6 Hz, 2H, H-14), 1.49 (m, 2H, H-8), 1.42–1.27 (m,6H, H-15, H-16, H-17), 0.88 (t, J = 6.9 Hz, 3H, H-18). ^13^C NMR (125 MHz, CDCl_3_) δ 170.83, 170.32, 170.12, 169.79 (4 × C=O), 147.99 (C-12), 143.11 (C_ar_), 128.90 (2 × CH_ar_), 127.97 (C_ar_), 125.63 (2 × CH_ar_), 119.16 (C-11), 74.51 (C-3), 69.42 (C-4), 69.25 (C-2), 61.97 (C-6), 59.37 (C-5), 52.50 (C-1), 50.57 (C-7), 49.93 (C-10), 35.76 (C-13), 31.73 (C-14), 31.39 (C-15), 28.96 (C-16), 27.80 (C-9), 22.62 (C-17), 22.45 (C-8), 20.86, 20.83, 20.75, 20.69 (4 × CO*C*H_3_), 14.12 (C-18).

**(2R,3R,4R,5S)-2-(acetoxymethyl)-1-(6-(4-phenyl-1H-1,2,3-triazol-1-yl)hexyl)piperidine-3,4,5-triyl triacetate 27**

Prepared according to procedure B. Compound **22b** (200 mg, 0.44 mmol), phenylacetylene (89 mg, 0.88 mmol), CuSO_4_·5H_2_O (33 mg, 0.13 mmol), sodium ascorbate (52 mg, 0.26 mmol), DMF/H_2_O (3 mL, 2:1). Yield: 190 mg, 0.34 mmol, 78%, white solid, *R*_f_ = 0.17 (1:1; PE:EtOAc). ^1^H NMR (500 MHz, CDCl_3_) δ 7.86–7.81 (m, 2H, ArH), 7.80 (s, 1H, CH- triazole), 7.42 (t, J = 7.6 Hz, 2H, ArH), 7.32 (dd, J = 10.3, 4.3 Hz, 1H, ArH), 5.16–5.00 (m, 2H, H-6), 4.95 (td, J = 9.8, 5.1 Hz, 1H, H-2), 4.38 (t, J = 7.1 Hz, 2H, H-12), 4.14 (qd, J = 12.9, 2.5 Hz, 2H, H-3, H-4), 3.18 (dd, J = 11.4, 5.0 Hz, 1H, H-1a), 2.77–2.66 (m, 1H, H-7a), 2.61 (d, J = 8.8 Hz, 1H, H-5), 2.57–2.46 (m, 1H, H-7b), 2.28 (t, J = 10.9 Hz, 1H, H-1b), 2.09–1.89 (m, 14H, 4×COCH_3_, H-11), 1.53–1.31 (m, 6H, H-8, H-9, H-10). ^13^C NMR (125 MHz, CDCl_3_) δ 170.86, 170.33, 170.07, 169.77 (4 × C=O), 147.70 (C-14), 130.70 (C_ar_), 128.83 (2 × CH_ar_), 128.09 (C_ar_), 125.67 (2 × CH_ar_), 119.59 (C-13), 74.63 (C-3), 69.51 (C-4), 69.38 (C-2), 61.62 (C-6), 59.49 (C-5), 52.79 (C-1), 51.49 (C-7), 50.23 (C-12), 30.25 (C-11), 26.56 (C-8), 26.32 (C-9), 24.78 (C-10), 20.86, 20.83, 20.74, 20.68 (4 × CO*C*H_3_).

**(2R,3R,4R,5S)-2-(acetoxymethyl)-1-(6-(4-(p-tolyl)-1H-1,2,3-triazol-1-yl)hexyl)piperidine-3,4,5-triyl triacetate 28**

Prepared according to procedure B. Compound **22b** (200 mg, 0.44 mmol), 4-ethynyltoluene (102 mg, 0.88 mmol), CuSO_4_·5H_2_O (33 mg, 0.13 mmol), sodium ascorbate (52 mg, 0.26 mmol), DMF/H_2_O (3 mL, 2:1). Yield: 180 mg, 0.32 mmol, 72%, white solid, *R*_f_ = 0.21 (1:1; PE:EtOAc). ^1^H NMR (500 MHz, CDCl_3_) δ 7.86–7.66 (m, 3H, ArH, CH- triazole), 7.24 (d, J = 7.9 Hz, 2H, ArH), 5.17–5.00 (m, 2H, H-6), 5.00–4.91 (m, 1H, H-2), 4.39 (t, J = 7.1 Hz, 2H, H-12), 4.16 (qd, J = 12.9, 2.4 Hz, 2H, H-3, H-4), 3.20 (dd, J = 11.4, 5.0 Hz, 1H, H-1a), 2.81–2.69 (m, 1H, H-7a), 2.63 (d, J = 8.8 Hz, 1H, H-5), 2.55–2.48 (m, 1H, H-7b), 2.39 (s, 3H, H-15), 2.30 (t, J = 10.9 Hz, 1H, H-1b), 2.12–1.88 (m, 14H, 4 × COCH_3_, H-11), 1.55–1.32 (m, 6H, H-8, H-9, H-10). ^13^C NMR (125 MHz, CDCl_3_) δ 170.89, 170.36, 170.09, 169.78 (4 × C=O), 147.81 (C-14), 137.92 (C_ar_), 129.52 (2 × CH_ar_), 127.87 (C_ar_), 125.60 (2 × CH_ar_), 119.21 (C-13), 74.65 (C-3), 69.52 (C-4), 69.39 (C-2), 61.63 (C-6), 59.51 (C-5), 52.81 (C-1), 51.51 (C-7), 50.22 (C-12), 30.27 (C-11), 26.58 (C-8), 26.34 (C-9), 24.78 (C-10), 21.29 (C-15), 20.87, 20.84, 20.75, 20.69 (4 × CO*C*H_3_).

**(2R,3R,4R,5S)-2-(acetoxymethyl)-1-(6-(4-(4-ethylphenyl)-1H-1,2,3-triazol-1-yl)hexyl)piperidine-3,4,5-triyl triacetate 29**

Prepared according to procedure B. Compound **22b** (200 mg, 0.44 mmol), 1-ethyl-4-ethynylbenzene (114 mg, 0.88 mmol), CuSO_4_·5H_2_O (33 mg, 0.13 mmol), sodium ascorbate (52 mg, 0.26 mmol), DMF/H_2_O (3 mL, 2:1). Yield: 220 mg, 0.38 mmol, 86%, white solid, *R*_f_ =0.25 (1:1; PE:EtOAc). ^1^H NMR (500 MHz, CDCl_3_) δ 7.89–7.67 (m, 3H, ArH, CH- triazole), 7.24 (d, J = 7.4 Hz, 2H, ArH), 5.13–4.99 (m, 2H, H-6), 4.95 (dd, J = 9.2, 4.9 Hz, 1H, H-2), 4.36 (t, J = 6.7 Hz, 2H, H-12), 4.21–4.06 (m, 2H, H-3, H-4), 3.17 (dd, J = 11.3, 4.4 Hz, 1H, H-1a), 2.72–2.50 (m, 5H, H-5, H-7, H-15), 2.28 (t, J = 10.8 Hz, 1H, H-1b), 2.16–1.87 (m, 14H, 4×COCH_3_, H-11), 1.55–1.23 (m, 8H, H-8, H-9, H-10, H-16). ^13^C NMR (125 MHz, CDCl_3_) δ 170.79, 170.26, 170.00, 169.72 (4 × C=O), 147.69 (C-14), 144.21 (C_ar_), 128.28 (2 × CH_ar_), 128.11 (C_ar_), 125.63 (2 × CH_ar_), 119.32 (C-13), 74.61 (C-3), 69.48 (C-4), 69.34 (C-2), 61.57 (C-6), 59.45 (C-5), 52.75 (C-1), 51.47 (C-7), 50.14 (C-12), 30.20 (C-11), 28.62 (C-15), 26.52 (C-8), 26.27 (C-9), 24.71 (C-10), 20.81, 20.77, 20.69, 20.63 (4 × CO*C*H_3_), 15.52 (C-16).

**(2R,3R,4R,5S)-2-(acetoxymethyl)-1-(6-(4-(4-propylphenyl)-1H-1,2,3-triazol-1-yl)hexyl)piperidine-3,4,5-triyl triacetate 30**

Prepared according to procedure B. Compound **22b** (200 mg, 0.44 mmol), 1-eth-1-ynyl-4-propylbenzene (126.9 mg, 0.88 mmol), CuSO_4_·5H_2_O (33 mg, 0.13 mmol), sodium ascorbate (52 mg, 0.26 mmol), DMF/H_2_O (3 mL, 2:1). Yield: 230 mg, 0.38 mmol, 87%, white solid, *R*_f_ = 0.31 (1:1; PE:EtOAc). ^1^H NMR (500 MHz, CDCl_3_) δ 7.82 (s, 1H, CH- triazole), 7.75 (d, J = 7.9 Hz, 2H, ArH), 7.22 (d, J = 7.9 Hz, 2H, ArH), 5.16–4.99 (m, 2H, H-6), 4.96 (dd, J = 9.4, 4.9 Hz, 1H, H-2), 4.36 (t, J = 6.9 Hz, 2H, H-12), 4.16 (d, J = 12.6 Hz, 2H, H-3, H-4), 3.18 (dd, J = 11.3, 4.9 Hz, 1H, H-1a), 2.72 (dt, J = 14.4, 7.6 Hz, 1H, H-7a), 2.60 (t, J = 7.5 Hz, 3H, H-5, H-15), 2.56–2.45 (m, 1H, H-7b), 2.29 (t, J = 10.8 Hz, 1H, H-1b), 2.13–1.81 (m, 14H, 4×COCH_3_, H-11), 1.66 (dd, J = 14.9, 7.4 Hz, 2H, H-16), 1.51–1.21 (m, 6H, H-8, H-9, H-10), 0.95 (t, J = 7.3 Hz, 3H, H-17). ^13^C NMR (125 MHz, CDCl_3_) δ 170.67, 170.16, 169.91, 169.65 (4 × C=O), 147.59 (C-14), 142.54 (C_ar_), 128.84 (2 × CH_ar_), 128.13 (C_ar_), 125.48 (2 × CH_ar_), 119.38 (C-13), 74.56 (C-3), 69.44 (C-4), 69.30 (C-2), 61.51 (C-6), 59.40 (C-5), 52.70 (C-1), 51.43 (C-7), 50.06 (C-12), 37.69 (C-15), 30.14 (C-11), 26.46 (C-8), 26.20 (C-9), 24.64 (C-10), 24.40 (C-16), 20.74, 20.71, 20.62, 20.57 (4 × CO*C*H_3_), 13.72 (C-17).

**(2R,3R,4R,5S)-2-(acetoxymethyl)-1-(6-(4-(4-butylphenyl)-1H-1,2,3-triazol-1-yl)hexyl)piperidine-3,4,5-triyl triacetate 31**

Prepared according to procedure B. Compound **22b** (200 mg, 0.44 mmol), 1-butyl-4-eth-1-ynylbenzene (138 mg, 0.88 mmol), CuSO_4_·5H_2_O (33 mg, 0.13 mmol), sodium ascorbate (52 mg, 0.26 mmol), DMF/H_2_O (3 mL, 2:1). Yield: 230 mg, 0.37 mmol, 85%, white solid, *R*_f_ =0.35 (1:1; PE:EtOAc). ^1^H NMR (500 MHz, CDCl_3_) δ 7.82–7.69 (m, 3H, ArH, CH- triazole), 7.22 (d, J = 6.3 Hz, 2H, ArH), 5.03 (dd, J = 15.8, 7.9 Hz, 2H, H-6), 4.95 (m, 1H, H-2), 4.37 (d, J = 5.0 Hz, 2H, H-12), 4.14 (d, J = 3.6 Hz, 2H, H-3, H-4), 3.17 (d, J = 6.7 Hz, 1H, H-1a), 2.72 (m, 1H, H-7a), 2.62 (m, 3H, H-5, H-15), 2.52 (m, 1H, H-7b), 2.28 (t, J = 10.7 Hz, 1H, H-1b), 2.14–1.87 (m, 14H, 4 × COCH_3_, H-11), 1.68–1.55 (m, 2H, H-16), 1.50–1.20 (m, 8H, H-8, H-9, H-10, H-17), 0.92 (t, J = 5.9 Hz, 3H, H-18). ^13^C NMR (125 MHz, CDCl_3_) δ 170.82, 170.30, 170.04, 169.74 (4 × C=O), 147.77 (C-14), 142.94 (C_ar_), 128.86 (2 × CH_ar_), 128.06 (C_ar_), 125.57(2 × CH_ar_), 119.27 (C-13), 74.63 (C-3), 69.50 (C-4), 69.36 (C-2), 61.60 (C-6), 59.47 (C-5), 52.78 (C-1), 51.49 (C-7), 50.17 (C-12), 35.40 (C-15), 33.54 (C-16), 30.23 (C-11), 26.55 (C-8), 26.30 (C-9), 24.74 (C-10), 22.30 (C-17), 20.84, 20.80, 20.72, 20.66 (4 × CO*C*H_3_), 13.95 (C-18).

**(2R,3R,4R,5S)-2-(acetoxymethyl)-1-(6-(4-(4-pentylphenyl)-1H-1,2,3-triazol-1-yl)hexyl)piperidine-3,4,5-triyl triacetate 32**

Prepared according to procedure B. Compound **22b** (200 mg, 0.44 mmol), 1-ethynyl-4-pentylbenzene (151 mg, 0.88 mmol), CuSO_4_·5H_2_O (33 mg, 0.13 mmol), sodium ascorbate (52 mg, 0.26 mmol), DMF/H_2_O (3 mL, 2:1). Yield: 250 mg, 0.4 mmol, 91%, white solid, *R*_f_ = 0.38 (1:1; PE:EtOAc). ^1^H NMR (500 MHz, CDCl_3_) δ 7.74 (m, 3H, ArH, CH-triazole), 7.23 (d, J = 8.0 Hz, 2H, ArH), 5.13–5.00 (m, 2H, H-6), 4.95 (dd, J = 14.0, 9.4 Hz, 1H, H-2), 4.38 (t, J = 6.9 Hz, 2H, H-12), 4.15 (qd, J = 13.0, 2.5 Hz, 2H, H-3, H-4), 3.18 (dd, J = 11.3, 4.9 Hz, 1H, H-1a), 2.72 (dd, J = 11.8, 7.8 Hz, 1H, H-7a), 2.62 (m, 3H, H-5, H-15), 2.51 (dd, J = 15.9, 6.9 Hz, 1H, H-7b), 2.28 (t, J = 10.8 Hz, 1H, H-1b), 2.12–1.85 (m, 14H, 4 × COCH_3_, H-11), 1.70–1.57 (m, 2H, H-16), 1.52–1.19 (m, 10H, H-8, H-9, H-10, H-17, H-18), 0.89 (d, J = 7.0 Hz, 3H, H-19). ^13^C NMR (125 MHz, CDCl_3_) δ 170.87, 170.34, 170.07, 169.77 (4 × C=O), 147.83 (C-14), 143.01 (C_ar_), 128.87 (2 × CH_ar_), 128.07 (C_ar_), 125.60 (2 × CH_ar_), 119.22 (C-13), 74.65 (C-3), 69.51 (C-4), 69.38 (C-2), 61.62 (C-6), 59.49 (C-5), 52.81 (C-1), 51.51 (C-7), 50.19 (C-12), 35.70 (C-15), 31.46 (C-16), 31.09 (C-17), 30.26 (C-11), 26.57 (C-8), 26.33 (C-9), 24.77 (C-10), 22.54 (C-18), 20.86, 20.83, 20.74, 20.68 (4 × CO*C*H_3_), 14.05 (C-19).

**(2R,3R,4R,5S)-2-(acetoxymethyl)-1-(6-(4-(4-hexylphenyl)-1H-1,2,3-triazol-1-yl)hexyl)piperidine-3,4,5-triyl triacetate 33**

Prepared according to procedure B. Compound **22b** (200 mg, 0.44 mmol), 1-ethynyl-4-hexylbenzene (163 mg, 0.88 mmol), CuSO_4_·5H_2_O (33 mg, 0.13 mmol), sodium ascorbate (52 mg, 0.26 mmol), DMF/H_2_O (3 mL, 2:1). Yield: 220 mg, 0.34 mmol, 78%, white solid, *R*_f_ =0.41 (1:1; PE:EtOAc). ^1^H NMR (500 MHz, CDCl_3_) δ 7.84–7.69 (m, 3H, ArH, CH-triazole), 7.31–7.17 (m, 2H, ArH), 5.15–5.01 (m, 2H, H-6), 4.97 (dd, J = 9.4, 4.6 Hz, 1H, H-2), 4.38 (q, J = 6.7 Hz, 2H, H-12), 4.24–4.08 (m, 2H, H-3, H-4), 3.26–3.10 (m, 1H, H-1a), 2.74 (m, 1H, H-7a), 2.63 (t, J = 10.0 Hz, 3H, H-5, H-15), 2.53 (td, J = 9.1, 4.6 Hz, 1H, H-7b), 2.30 (td, J = 11.2, 4.9 Hz, 1H, H-1b), 2.13–1.85 (m, 14H, 4×COCH_3_, H-11), 1.71–1.53 (m, 2H, H-16), 1.50–1.22 (m, 12H, H-8, H-9, H-10, H-17, H-18, H-19), 0.98–0.74 (m, 3H, H-20). ^13^C NMR (125 MHz, CDCl_3_) δ 170.80, 170.27, 170.01, 169.73 (4 × C=O), 147.76 (C-14), 142.94 (C_ar_), 128.84 (2 × CH_ar_), 128.08 (C_ar_), 125.56 (2 × CH_ar_), 119.25 (C-13), 74.63 (C-3), 69.50 (C-4), 69.36 (C-2), 61.60 (C-6), 59.47 (C-5), 52.78 (C-1), 51.49 (C-7), 50.15 (C-12), 35.71 (C-15), 31.69 (C-16), 31.35 (C-17), 30.23 (C-11), 28.91 (C-18), 26.54 (C-8), 26.29 (C-9), 24.75 (C-10), 22.58 (C-19), 20.82, 20.80, 20.70, 20.65 (4 × CO*C*H_3_), 14.09 (C-20).

### 3.4. General Procedure C—Deacetylation Reaction of Compounds ***23***–***33***

Compounds **23–33** were dissolved in methanol, and NaOMe (1.0 M in methanol) was added until pH 9.0 was achieved. After stirring overnight at room temperature, the mixture was neutralized with ion exchange resin DOWEX^®^ 50WX4-50 (H^+^), filtered and concentrated under reduced pressure. The resulting product (compounds **10–20**) was obtained in quantitative yield.

**(2R,3R,4R,5S)-1-(4-(4-(4-ethylphenyl)-1H-1,2,3-triazol-1-yl)butyl)-2-(hydroxymethyl)piperidine-3,4,5-triol 10**

Prepared according to procedure C. Compound **23** (190 mg, 0.34 mmol). Yield: 130 mg, 0.33 mmol, 96%, white solid, *R*_f_ = 0.22 (3:1; CH_2_Cl_2_: MeOH + 1% NH_4_OH), [α]_D_^25^ 22.6 (*c* 0.5, H_2_O). ^1^H NMR (500 MHz, D_2_O) δ 7.78 (s, 1H, CH- triazole), 7.42 (d, J = 7.4 Hz, 2H, ArH), 6.73 (d, J = 7.8 Hz, 2H, ArH), 4.09 (m, 2H, H-10), 3.69 (dd, J = 20.9, 12.1 Hz, 2H, H-6), 3.49 (d, J = 4.6 Hz, 1H, H-2), 3.33 (t, J = 9.4 Hz, 1H, H-3), 3.16 (t, J = 9.1 Hz, 1H, H-4), 2.86 (d, J = 6.2 Hz, 1H, H-1a), 2.58 (m, 1H, H-7a), 2.37 (m, 1H, H-7b), 2.17–1.88 (m, 4H, H-5, H-1b, H-13), 1.52 (m, 2H, H-9), 1.26 (m, 2H. H-8), 0.77 (t, J = 7.4 Hz, 3H, H-14). ^13^C NMR (125 MHz, D_2_O) δ 147.02 (C-12), 143.61 (C_ar_), 128.09 (2 × CH_ar_), 127.62 (C_ar_), 125.40 (2 × CH_ar_), 120.74 (C-13), 78.35 (C-3), 69.80 (C-4), 68.75 (C-2), 65.25 (C-6), 57.26 (C-5), 55.46 (C-1), 51.30 (C-7), 49.93 (C-10), 28.15 (C-13), 27.88 (C-9), 20.35 (C-8), 14.95 (C-14). HRMS (ESI) *m*/*z* calcd for C_20_H_31_N_4_O_4_^+^ [M+H]^+^ 391.23398, found 391.23346.

**(2R,3R,4R,5S)-2-(hydroxymethyl)-1-(4-(4-(4-propylphenyl)-1H-1,2,3-triazol-1-yl)butyl)piperidine-3,4,5-triol 11**

Prepared according to procedure C. Compound **24** (150 mg, 0.26 mmol). Yield:100 mg, 0.25 mmol, 95%, white solid, *R*_f_ = 0.25 (3:1; CH_2_Cl_2_: MeOH + 1% NH_4_OH), [α]_D_^25^ −10.7 (*c* 0.35, H_2_O). ^1^H NMR (500 MHz, D_2_O) δ 7.84 (s, 1H, CH-triazole), 7.45 (d, J = 6.6 Hz, 2H, ArH), 6.72 (d, J = 7.1 Hz, 2H, ArH), 4.11 (m, 2H, H-10), 3.70 (dd, J = 21.6, 12.4 Hz, 2H, H-6), 3.50 (dd, J = 13.8, 9.3 Hz, 1H, H-2), 3.34 (t, J = 9.3 Hz, 1H, H-3), 3.17 (t, J = 9.0 Hz, 1H, H-4), 2.87 (d, J = 5.5 Hz, 1H, H-1a), 2.59 (m, 1H, H-7a), 2.38 (m, 1H, H-7b), 2.16–1.93 (m, 4H, H-5, H-1b, H-13), 1.53 (m, 2H, H-9), 1.38–1.07 (m, 4H, H-8, H-14), 0.59 (t, J = 6.6 Hz, 3H, H-15). ^13^C NMR (125 MHz, D_2_O) δ 147.01 (C-12), 141.95 (C_ar_), 128.63 (2 × CH_ar_), 127.81(C_ar_), 125.35(2 × CH_ar_), 120.74 (C-13), 78.34 (C-3), 69.76 (C-4), 68.71 (C-2), 65.34 (C-6), 57.22 (C-5), 55.47 (C-1), 51.35 (C-7), 49.96 (C-10), 37.45 (C-13), 27.91 (C-9), 24.06 (C-14), 20.47 (C-8), 13.60 (C-15). HRMS (ESI) *m*/*z* calcd for C_21_H_33_N_4_O_4_^+^ [M+H]^+^ 405.24963, found 405.24997.

**(2R,3R,4R,5S)-1-(4-(4-(4-butylphenyl)-1H-1,2,3-triazol-1-yl)butyl)-2-(hydroxymethyl)piperidine-3,4,5-triol 12**

Prepared according to procedure C. Compound **25** (200 mg, 0.34 mmol). Yield: 130 mg, 0.31 mmol, 93%, white solid, *R*_f_ = 0.31 (3:1; CH_2_Cl_2_: MeOH + 1% NH_4_OH), [α]_D_^25^ −12.1 (*c* 0.39, H_2_O). ^1^H NMR (500 MHz, D_2_O) δ 7.83 (s, 1H, CH-triazole), 7.46 (s, 2H, ArH), 6.72 (s, 2H, ArH), 4.10 (m, 2H, H-10), 3.68 (d, J = 19.6 Hz, 2H, H-6), 3.49 (m, 1H, H-2), 3.33 (t, J = 8.8 Hz, 1H, H-3), 3.15 (t, J = 8.4 Hz, 1H, H-4), 2.86 (m, 1H, H-1a), 2.58 (m, 1H, H-7a), 2.36 (m, 1H, H-7b), 2.18–1.94 (m, 4H, H-5, H-1b, H-13), 1.51 (m, 2H, H-9), 1.39–0.91 (m, 6H, H-8, H-14, H-15), 0.65 (m, 3H, H-16). ^13^C NMR (125 MHz, D_2_O) δ 146.96 (C-12), 142.05 (C_ar_), 128.52 (2 × CH_ar_), 127.82(C_ar_), 125.35(2 × CH_ar_), 120.74 (C-13), 78.35 (C-3), 69.74 (C-4), 68.70 (C-2), 65.34 (C-6), 57.20 (C-5), 55.48 (C-1), 51.35 (C-7), 49.96 (C-10), 35.15 (C-13), 33.19 (C-14), 27.94 (C-9), 22.37 (C-15), 20.50 (C-8), 13.72 (C-16). HRMS (ESI) *m*/*z* calcd for C_22_H_35_N_4_O_4_^+^ [M+H]^+^ 419.26528, found 419.26492.

**(2R,3R,4R,5S)-1-(4-(4-(4-hexylphenyl)-1H-1,2,3-triazol-1-yl)butyl)-2-(hydroxymethyl)piperidine-3,4,5-triol 13**

Prepared according to procedure C. Compound **26** (200 mg, 0.33 mmol). Yield: 140 mg, 0.31 mmol, 95%, white solid, *R*_f_ = 0.34 (3:1; CH_2_Cl_2_: MeOH + 1% NH_4_OH), [α]_D_^25^ 7 (*c* 0.2, H_2_O). ^1^H NMR (500 MHz, D_2_O) δ 7.84 (s, 1H, CH-triazole), 7.49 (s, 2H, ArH), 6.78 (s, 2H, ArH), 4.09 (m, 2H, H-10), 3.71 (m, 2H, H-6), 3.50 (m, 1H, H-2), 3.34 (m, 1H, H-3), 3.16 (m, 1H, H-4), 2.87 (m, 1H, H-1a), 2.59 (m, 1H, H-7a), 2.36 (m, 1H, H-7b), 2.05 (m, 4H, H-5, H-1b, H-13), 1.25 (m, 12H, H-8, H-9, H-14, H-15, H-16, H-17), 0.80 (m, 3H, H-18). ^13^C NMR (125 MHz, D_2_O) δ 146.94(C-12), 142.05(C_ar_), 128.49 (2 × CH_ar_), 128.01(C_ar_), 125.40(2 × CH_ar_), 120.70 (C-13), 78.48 (C-3), 69.85 (C-4), 68.79 (C-2), 65.43 (C-6), 57.34 (C-5), 55.62 (C-1), 51.43 (C-7), 49.99 (C-10), 35.64 (C-13), 31.91 (C-14), 31.20 (C-15), 29.60 (C-16), 28.02 (C-9), 22.83 (C-17), 20.69 (C-8), 14.01 (C-18). HRMS (ESI) *m*/*z* calcd for C_24_H_39_N_4_O_4_^+^ [M+H]^+^ 447.29658, found 447.29654.

**(2R,3R,4R,5S)-2-(hydroxymethyl)-1-(6-(4-phenyl-1H-1,2,3-triazol-1-yl)hexyl)piperidine-3,4,5-triol 14**

Prepared according to procedure C. Compound **27** (122 mg, 0.22 mmol). Yield: 80 mg, 0.2 mmol, 93%, white solid, *R*_f_ = 0.21 (3:1; CH_2_Cl_2_: MeOH + 1% NH_4_OH), [α]_D_^25^ −8.2 (*c* 0.28, CH_3_OH). ^1^H NMR (500 MHz, CD_3_OD) δ 8.31 (s, 1H, CH- triazole), 7.81 (d, J = 7.4 Hz, 2H, ArH), 7.42 (t, J = 7.6 Hz, 2H, ArH), 7.33 (t, J = 7.4 Hz, 1H, ArH), 4.43 (t, J = 7.1 Hz, 2H, H-12), 3.85 (d, J = 2.1 Hz, 2H, H-6), 3.50 (td, J = 10.1, 4.8 Hz, 1H, H-2), 3.37 (t, J = 9.3 Hz, 1H, H-3), 3.16 (t, J = 9.1 Hz, 1H, H-4), 3.02 (dd, J = 11.2, 4.8 Hz, 1H, H-1a), 2.90–2.75 (m, 1H, H-7a), 2.65–2.56 (m, 1H, H-7b), 2.22 (m, 2H, H-5, H-1b), 1.94 (dd, J = 14.1, 7.0 Hz, 2H, H-11), 1.50 (m, 2H, H-8), 1.34 (m, 4H, H-9, H-10). ^13^C NMR (125 MHz, CD_3_OD) δ 147.42 (C-14), 130.35 (C_ar_), 128.63 (2 × CH_ar_), 127.97 (CH_ar_), 125.27 (2 × CH_ar_), 120.84 (C-13), 78.93 (C-3), 70.32 (C-4), 69.07 (C-2), 66.00 (C-6), 57.69 (C-5), 56.02 (C-1), 52.24 (C-7), 50.01 (C-12), 29.81 (C-11), 26.45 (C-8), 25.95 (C-9), 23.59 (C-10). HRMS (ESI) *m*/*z* calcd for C_20_H_31_N_4_O_4_^+^ [M+H]^+^ 391.23398, found 391.23373.

**(2R,3R,4R,5S)-2-(hydroxymethyl)-1-(6-(4-(p-tolyl)-1H-1,2,3-triazol-1-yl)hexyl)piperidine-3,4,5-triol 15**

Prepared according to procedure C. Compound **28** (152 mg, 0.27 mmol). Yield: 100 mg, 0.25 mmol, 94%, white solid, *R*_f_ = 0.25 (3:1; CH_2_Cl_2_: MeOH + 1% NH_4_OH), [α]_D_^25^ −7.1 (*c* 0.35, CH_3_OH). ^1^H NMR (500 MHz, CD_3_OD) δ 8.23 (s, 1H, CH- triazole), 7.68 (d, J = 7.9 Hz, 2H, ArH), 7.22 (d, J = 7.9 Hz, 2H, ArH), 4.39 (t, J = 7.0 Hz, 2H, H-12), 3.85 (m, 2H, H-6), 3.50 (td, J = 9.9, 4.8 Hz, 1H, H-2), 3.38 (t, J = 9.3 Hz, 1H, H-3), 3.17 (t, J = 9.1 Hz, 1H, H-4), 3.00 (dd, J = 11.1, 4.6 Hz, 1H, H-1a), 2.88–2.70 (m, 1H, H-7a), 2.58 (d, J = 6.6 Hz, 1H, H-7b), 2.33 (s, 3H, PhCH_3_, H-15), 2.25–2.09 (m, 2H, H-5, H-1b), 1.92 (dd, J = 14.2, 7.8 Hz, 2H, H-11), 1.47 (m, 2H, H-8), 1.28 (m, 4H, H-9, H-10). ^13^C NMR (125 MHz, CD_3_OD) δ 147.48 (C-14), 137.99 (C_ar_), 129.26 (2 × CH_ar_), 127.51 (C_ar_), 125.26 (2 × CH_ar_), 120.50 (C-13), 79.04 (C-3), 70.45 (C-4), 69.20 (C-2), 65.97 (C-6), 57.86 (C-5), 56.14 (C-1), 52.24 (C-7), 50.00 (C-12), 29.84 (C-11), 26.51 (C-8), 25.99 (C-9), 23.61 (C-10), 20.04 (C-15). HRMS (ESI) *m*/*z* calcd for C_21_H_33_N_4_O_4_^+^ [M+H]^+^ 405.24963, found 405.24973.

**(2R,3R,4R,5S)-1-(6-(4-(4-ethylphenyl)-1H-1,2,3-triazol-1-yl)hexyl)-2-(hydroxymethyl)piperidine-3,4,5-triol 16**

Prepared according to procedure C. Compound **29** (205 mg, 0.35 mmol). Yield: 140 mg, 0.33 mmol, 93%, white solid, *R*_f_ = 0.26 (3:1; CH_2_Cl_2_: MeOH + 1% NH_4_OH), [α]_D_^25^ −9.1 (*c* 0.5, CH_3_OH). ^1^H NMR (500 MHz, D_2_O) δ 7.88 (s, 1H, CH-triazole), 7.55 (s, 2H, ArH), 6.84 (s, 2H, ArH), 4.11 (m, 2H, H-12), 3.79 (m, 2H, H-6), 3.58 (m, 1H, H-2), 3.43 (m, 1H, H-3), 3.25 (d, J = 14.5 Hz, 1H, H-4), 2.95 (m, 1H, H-1a), 2.52 (m, 2H, H-7), 2.19 (m, 4H, H-5, H-1b, H-15), 1.58 (m, 2H, H-11), 1.23 (m, 2H, H-8), 0.92 (m, 7H, H-9, H-10, H-16). ^13^C NMR (125 MHz, D_2_O) δ 147.04 (C-14), 143.66 (C_ar_), 128.13 (2 × CH_ar_), 127.75 (C_ar_), 125.42 (2 × CH_ar_), 120.62 (C-13), 78.44 (C-3), 69.89 (C-4), 68.82 (C-2), 65.26 (C-6), 57.39 (C-5), 55.65 (C-1), 52.10 (C-7), 50.19 (C-12), 30.03 (C-11), 28.23 (C-15), 26.51 (C-8), 25.92 (C-9), 22.96 (C-10), 15.02 (C-16). HRMS (ESI) *m*/*z* calcd for C_22_H_35_N_4_O_4_^+^ [M+H]^+^ 419.26528, found 419.26465.

**(2R,3R,4R,5S)-2-(hydroxymethyl)-1-(6-(4-(4-propylphenyl)-1H-1,2,3-triazol-1-yl)hexyl)piperidine-3,4,5-triol 17**

Prepared according to procedure C. Compound **30** (228 mg, 0.38 mmol). Yield: 150 mg, 0.35 mmol, 92%, white solid, *R*_f_ = 0.28 (3:1; CH_2_Cl_2_: MeOH + 1% NH_4_OH), [α]_D_^25^ −8 (*c* 0.5, CH_3_OH). ^1^H NMR (500 MHz, D_2_O) δ 7.84 (s, 1H, CH- triazole), 7.49 (s, 2H, ArH), 6.73 (s, 2H, ArH), 4.06 (m, 2H, H-12), 3.74 (dd, J = 27.0, 10.8 Hz, 2H, H-6), 3.51 (m, 1H, H-2), 3.36 (t, J = 8.8 Hz, 1H, H-3), 3.19 (t, J = 8.5 Hz, 1H, H-4), 2.89 (m, 1H, H-1a), 2.46 (m, 2H, H-7), 2.11 (m, 4H, H-5, H-1b, H-15), 1.51 (m, 2H, H-11), 1.28–0.81 (m, 8H, H-8, H-9, H-10, H-16), 0.56 (m, 3H, H-17). ^13^C NMR (125 MHz, D_2_O) δ 147.00 (C-14), 141.88 (C_ar_), 128.62 (2 × CH_ar_), 127.94 (C_ar_), 125.31 (2 × CH_ar_), 120.61 (C-13), 78.46 (C-3), 69.89 (C-4), 68.82 (C-2), 65.30 (C-6), 57.42 (C-5), 55.71 (C-1), 52.09 (C-7), 50.20 (C-12), 37.44 (C-15), 30.09 (C-11), 26.54 (C-8), 25.97 (C-9), 24.07 (C-10), 22.99 (C-16), 13.56 (C-17). HRMS (ESI) *m*/*z* calcd for C_23_H_37_N_4_O_4_^+^ [M+H]^+^ 433.28093, found 433.28061.

**(2R,3R,4R,5S)-1-(6-(4-(4-butylphenyl)-1H-1,2,3-triazol-1-yl)hexyl)-2-(hydroxymethyl)piperidine-3,4,5-triol 18**

Prepared according to procedure C. Compound **31** (184 mg, 0.3 mmol). Yield: 120 mg, 0.27 mmol, 90%, white solid, *R*_f_ = 0.32 (3:1; CH_2_Cl_2_: MeOH + 1% NH_4_OH), [α]_D_^25^ −6.9 (*c* 0.35, CH_3_OH). ^1^H NMR (500 MHz, D_2_O) δ 7.86 (s, 1H, CH- triazole), 7.50 (s, 2H, ArH), 6.74 (s, 2H, ArH), 4.07 (m, 2H, H-12), 3.74 (dd, J = 15.4, 10.0 Hz, 2H, H-6), 3.52 (m, 1H, H-2), 3.36 (t, J = 8.6 Hz, 1H, H-3), 3.20 (t, J = 8.2 Hz, 1H, H-4), 2.90 (m, 1H, H-1a), 2.48 (m, 2H, H-7), 2.09 (m, 4H, H-5, H-1b, H-15), 1.53 (m, 2H, H-11), 1.09 (m, 10H, H-8, H-9, H-10, H-16, H-17), 0.63 (m, 3H, H-18). ^13^C NMR (125 MHz, D_2_O) δ 146.98 (C-14), 141.99 (C_ar_), 128.54 (2 × CH_ar_), 127.98 (C_ar_), 125.35 (2 × CH_ar_), 120.51 (C-13), 78.47 (C-3), 69.89 (C-4), 68.81 (C-2), 65.35 (C-6), 57.44 (C-5), 55.73 (C-1), 52.11(C-7), 50.23 (C-12), 35.16 (C-15), 33.18 (C-16), 30.15 (C-11), 26.59 (C-8), 26.02 (C-9), 23.08 (C-10), 22.34 (C-17), 13.71 (C-18). HRMS (ESI) *m*/*z* calcd for C_24_H_39_N_4_O_4_^+^ [M+H]^+^ 447.29658, found 447.29678.

**(2R,3R,4R,5S)-2-(hydroxymethyl)-1-(6-(4-(4-pentylphenyl)-1H-1,2,3-triazol-1-yl)hexyl)piperidine-3,4,5-triol 19**

Prepared according to procedure C. Compound **32** (214 mg, 0.34 mmol). Yield: 150 mg, 0.33 mmol, 96%, white solid, *R*_f_ = 0.36 (3:1; CH_2_Cl_2_: MeOH + 1% NH_4_OH), [α]_D_^25^ −7.5 (*c* 0.2, CH_3_OH). ^1^H NMR (500 MHz, D_2_O) δ 7.86 (s, 1H, CH- triazole), 7.51 (m, 2H, ArH), 6.75 (m, 2H, ArH), 4.04 (m, 2H, H-12), 3.73 (m, 2H, H-6), 3.51 (m, 1H, H-2), 3.34 (d, J = 7.6 Hz, 1H, H-3), 3.19 (m, 1H, H-4), 2.89 (m, 1H, H-1a), 2.47 (m, 2H, H-7), 2.10 (m, 4H, H-5, H-1b, H-15), 1.52 (m, 2H, H-11), 1.12 (m, 12H, H-8, H-9, H-10, H-16, H-17, H-18), 0.68 (m, 3H, H-19). ^13^C NMR (125 MHz, D_2_O) δ 149.40 (C-14), 144.41 (C_ar_), 130.96 (2 × CH_ar_), 130.52 (C_ar_), 127.83 (2 × CH_ar_), 123.01 (C-13), 80.97 (C-3), 72.37 (C-4), 71.29 (C-2), 67.85 (C-6), 59.93 (C-5), 58.23 (C-1), 54.59 (C-7), 52.69 (C-12), 37.95 (C-15), 34.29 (C-16), 33.32 (C-17), 32.66 (C-11), 29.08 (C-8), 28.55 (C-9), 25.60 (C-10), 24.97 (C-18), 16.41 (C-19). HRMS (ESI) *m*/*z* calcd for C_25_H_41_N_4_O_4_^+^ [M+H]^+^ 461.31223, found 461.31213.

**(2R,3R,4R,5S)-1-(6-(4-(4-hexylphenyl)-1H-1,2,3-triazol-1-yl)hexyl)-2-(hydroxymethyl)piperidine-3,4,5-triol 20**

Prepared according to procedure C. Compound **33** (160 mg, 0.25 mmol). Yield: 110 mg, 0.23 mmol, 92%, white solid, *R*_f_ = 0.4 (3:1; CH_2_Cl_2_: MeOH + 1% NH_4_OH), [α]_D_^25^ 7 (*c* 0.2, CH_3_OH). ^1^H NMR (500 MHz, D_2_O) δ 7.86 (s, 1H, CH- triazole), 7.52 (s, 2H, ArH), 6.77 (s, 2H, ArH), 4.04 (m, 2H, H-12), 3.76 (m, 2H, H-6), 3.52 (m, 1H, H-2), 3.36 (m, 1H, H-3), 3.20 (m, 1H, H-4), 2.91 (m, 1H, H-1a), 2.48 (m, 2H, H-7), 2.11 (m, 4H, H-5, H-1b, H-15), 1.53 (m, 2H, H-11), 1.35–0.63 (m, 17H, H-8, H-9, H-10, H-16, H-17, H-18, H-19, H-20). ^13^C NMR (125 MHz, D_2_O) δ 146.95 (C-14), 141.78 (C_ar_), 128.44 (2 × CH_ar_), 127.99 (C_ar_), 125.40 (2 × CH_ar_), 120.50 (C-13), 78.56 (C-3), 69.95 (C-4), 68.87 (C-2), 65.42 (C-6), 57.52 (C-5), 55.85 (C-1), 52.13 (C-7), 50.21 (C-12), 35.66 (C-15), 31.88 (C-16), 31.18 (C-17), 30.23 (C-11), 29.59 (C-18), 26.67 (C-8), 26.13 (C-9), 23.20 (C-10), 22.81 (C-19), 13.98 (C-20). HRMS (ESI) *m*/*z* calcd for C_26_H_43_N_4_O_4_^+^ [M+H]^+^ 475.32788, found 475.32794.

### 3.5. Glucosidase Inhibitory Assays

The α-glucosidase [32] and β-glucosidase assays [33] were performed in 50 mM phosphate buffer (pH 6.8) at 37 °C. The inhibitors were pre-incubated with the enzyme solutions at 37 °C for 15 min, and then the corresponding substrates were added. After the enzymatic reaction was incubated at 37 °C for 15 min., it was quenched by adding Na_2_CO_3_. Absorbance readings were taken on a BioTek µQuant Microplate Spectrophotometer at 405 nm. Experiments were performed in triplicate. The IC_50_ values were determined graphically using GraphPad Prism (version 8.0).

### 3.6. Kinetics of Enzyme Inhibition

The inhibition types of the selected compounds were determined from Lineweavere–Burk plots [34,35]. Inhibition constant (*K*_i_) was performed in 50 mM phosphate buffer (pH 6.8) at 37 °C, using para-nitrophenyl α-D-glucopyranoside as the substrate. The assay was initiated by adding α-glucosidase (*K*_m_ = 0.35 mM, yeast) to a solution of the substrate (concentrations used: 0.0625 mM, 0.125 mM, 0.25 mM, 0.5 mM, 1 mM) in the presence of inhibitors (concentrations used: 0 M, 3.125 µM, 12.5 µM). The enzyme reaction was performed according to the conditions described above.

### 3.7. Cell Proliferation Assay [36,37]

HL60 cells in DMEM supplemented with 10% fetal bovine serum were seeded at a density of 5 × 10^3^ cells/well in a 96-well plate and incubated at 37 °C for 24 h. The medium was substituted with fresh medium containing a range of concentrations of inhibitors in DMSO and cultured for another 24 h. CCK-8 solution (10 µL) was added to each well incubated at 37 °C for 1 h. The absorbance was read on a BioTek µQuant Microplate Spectrophotometer at 450 nm. The cell vitality curve was depicted with GraphPad Prism (version 8.0).

### 3.8. Molecular Docking Studies

The three-dimensional model of yeast α-glucosidase (MAL12, P53341) for docking studies, obtained from the SWISS-MODEL [38], was constructed by homology modeling using oligo-1,6-glucosidase from *S. cerevisiae* (PDB code: 3AXI, P53051) as the template (sequence identity: 72.12%). MOE Dock was used for the molecular docking of MAL12 with two ligands (DNJ and compound **20**). The 2D structure of ligands was converted to 3D in MOE through energy minimization. Then, LigX optimized the protonation state of the target and the orientation of the hydrogens. All docked poses of molecules were ranked by London dG scoring first; then, a force field refinement was carried out on the top 30 poses, followed by a rescoring of GBVI/WSA dG.

## 4. Conclusions

A range of DNJ derivatives were synthesized, and the preliminary glucosidase inhibition activities were evaluated in vitro. The structure–activity relationship studies had shown that the potency and selectivity were significantly dependent on the length of the linker and the alkyl chain at the 4-position of the phenyl group. These data confirmed our previous findings on the importance of alkyl chain length for enzyme inhibition. Compounds **17**, **18**, **19** and **20** were particularly potent α-glucosidase inhibitors compared to DNJ. Compound **20** was cytotoxic when viability was measured at 50 µM. Molecular docking studies showed that compound **18** was bound to the active site pocket of α-glucosidase through the hydrogen bond, hydrophobic and van der Waals interactions. Overall, these novel compounds represent new chemical scaffolds for developing potent and selecting glucosidase inhibitors.

## Figures and Tables

**Table 1:** Glycosidase inhibitory activity values of IC_50_^a^ (μM).

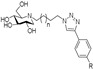 n = 1	α-Glucosidase	β-Glucosidase	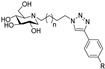 n = 3	α-Glucosidase	β-Glucosidase
7	551 ± 25	NI ^b^	14	130 ± 9	130 ± 5
8	324 ± 65	NI	16	68 ± 4	65 ± 2
10	244 ± 32	NI	17	14 ± 1	62 ± 4
11	171 ± 25	739 ± 78	18	11 ± 1	69 ± 5
12	105 ± 9	770 ± 92	19	11 ± 1	73 ± 7
9	63 ± 6	665 ± 57	20	16 ± 2	77 ± 4
13	82 ± 5	529 ± 41	DNJ	155 ± 15	648 ± 36

^a^ IC_50_ is defined as the compound concentration at which 50% of the activities of glucosidases. The values are mean ± SD from three independent experiments. ^b^ NI = no inhibition (less than 50% inhibition at 1 mM).

**Table 2:** Docking scores for DNJ and compound **18** against α-glucosidase.

Receptor	Ligand	Docking Scores (Kcal/mol)
MAL12	DNJ	−5.8
MAL12	**18**	−8.7

## Data Availability

Data is contained within the article.

## Supplementary Materials

The following supporting information can be downloaded at: https://www.mdpi.com/article/10.3390/molecules29215062/s1, Compounds characterization–Pages 2–31: ^1^H NMR, ^13^C NMR, and HRMS of all the target compounds. Homology modeling of Protein–Page 32.
