# Supplementary material for: Synthesis and Evaluation of Phenyltriazole-Deoxynojirimycin Hybrids as Potent α-Glucosidase Inhibitors
Source: Molecules. 2024 Oct 26;29(21):5062. doi: 10.3390/molecules29215062 (PMC11547804; doi:10.3390/molecules29215062)
Supplement: Supplementary file 1 [file molecules-29-05062-s001.zip › molecules-3238045-supplementary.pdf]

Synthesis and Evaluation of Phenyltriazole-deoxynojirimycin Hybrids as Potent α-Glucosidase Inhibitors

Lin Wang[1] , Wei Luo[1], Yonghong Zhao[2], Xinling Guo[1], Xiangru Bai[1], Leilei Guo[1], Nailiang Zhu[1] *

[1] School of Pharmacy, Xinyang Agriculture and Forestry University , Xinyang 464000, China
[2] Nanjing Institute for Food and Drug Control, Nanjing211198, China
* Correspondence: 2021260008@xyafu.edu.cn; Tel.: 15210647059

Supporting Information

List of Contents:

S2 to S31 NMR Spectra of compounds.
S32 Figures S1 showing homology modeling of protein.

NMR Spectra of compounds

^{1}H NMR (500 MHz, $CDCl_3$) spectrum of **22a**

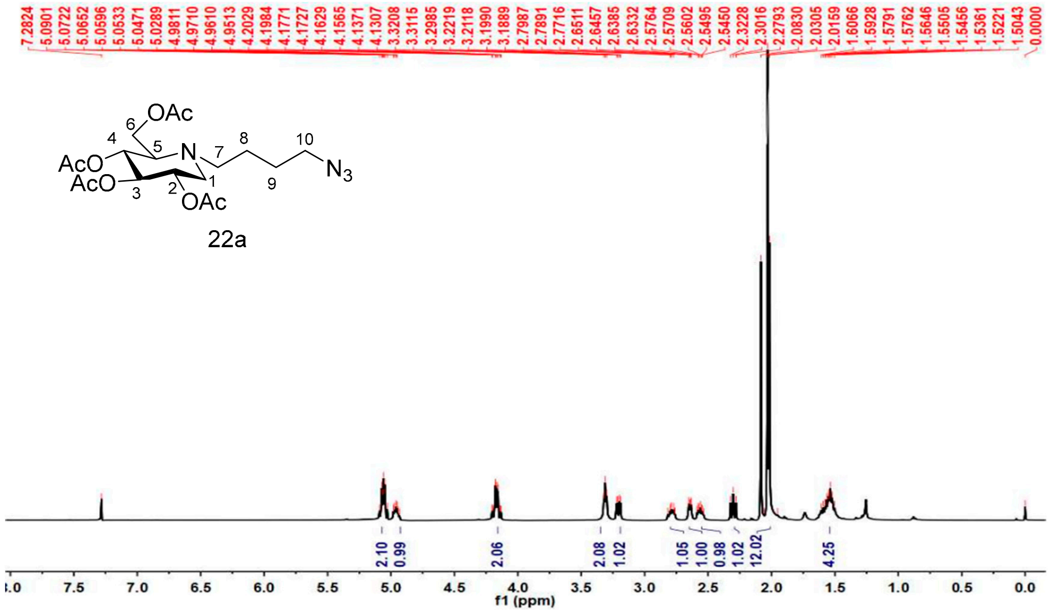

^{13}C NMR (125 MHz, $CDCl_3$) spectrum of **22a**

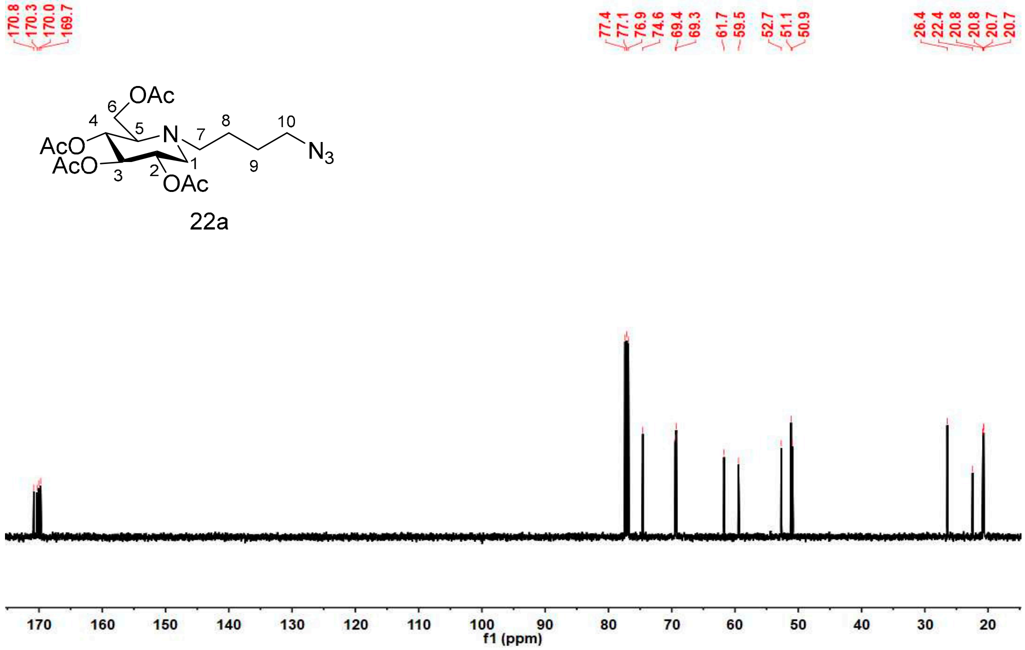

^{1}H NMR (500 MHz, $CDCl_3$) spectrum of **22b**

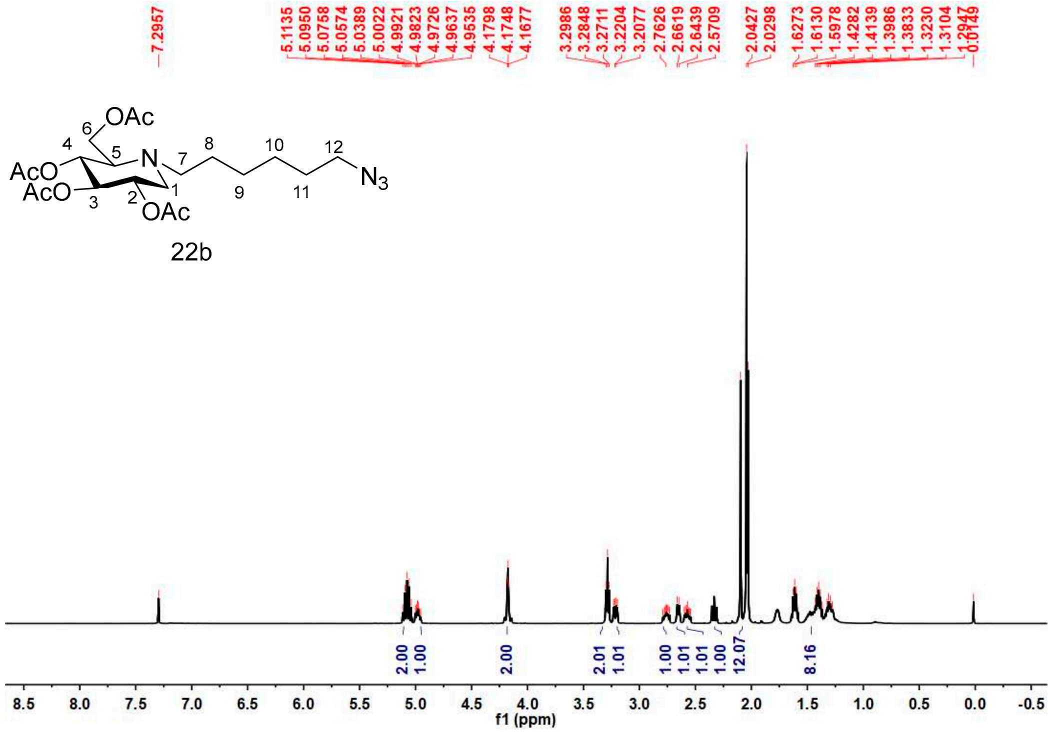

^{13}C NMR (125 MHz, $CDCl_3$) spectrum of **22b**

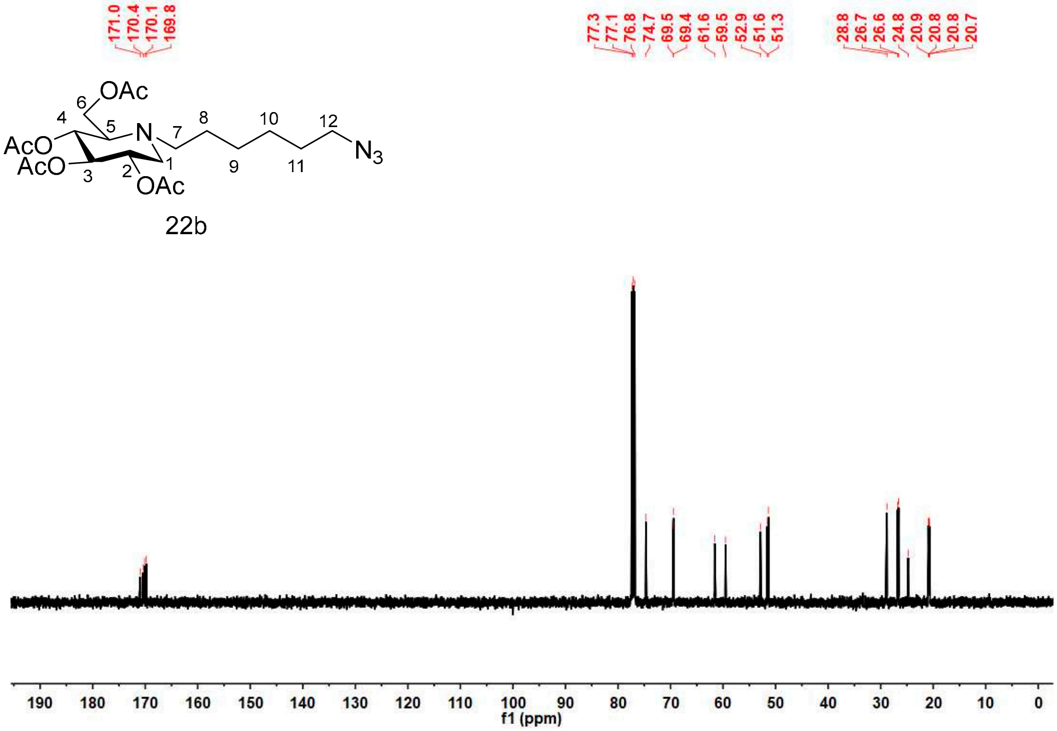

^{1}H NMR (500 MHz, $CDCl_3$) spectrum of **23**

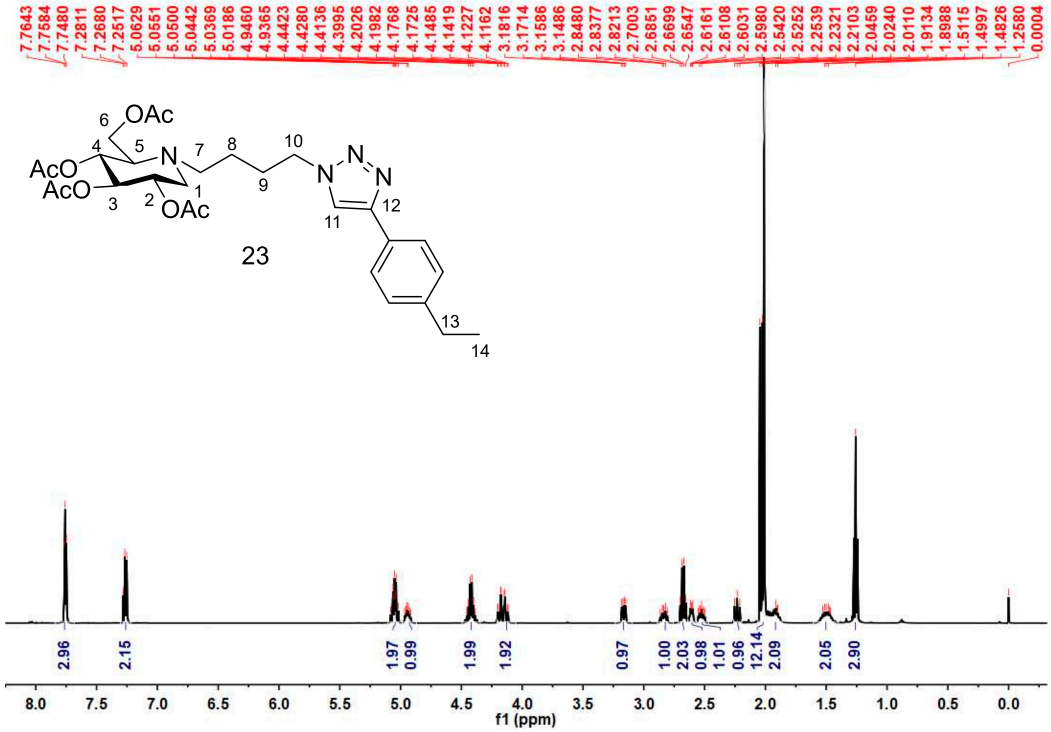

^{13}C NMR (125 MHz, $CDCl_3$) spectrum of **23**

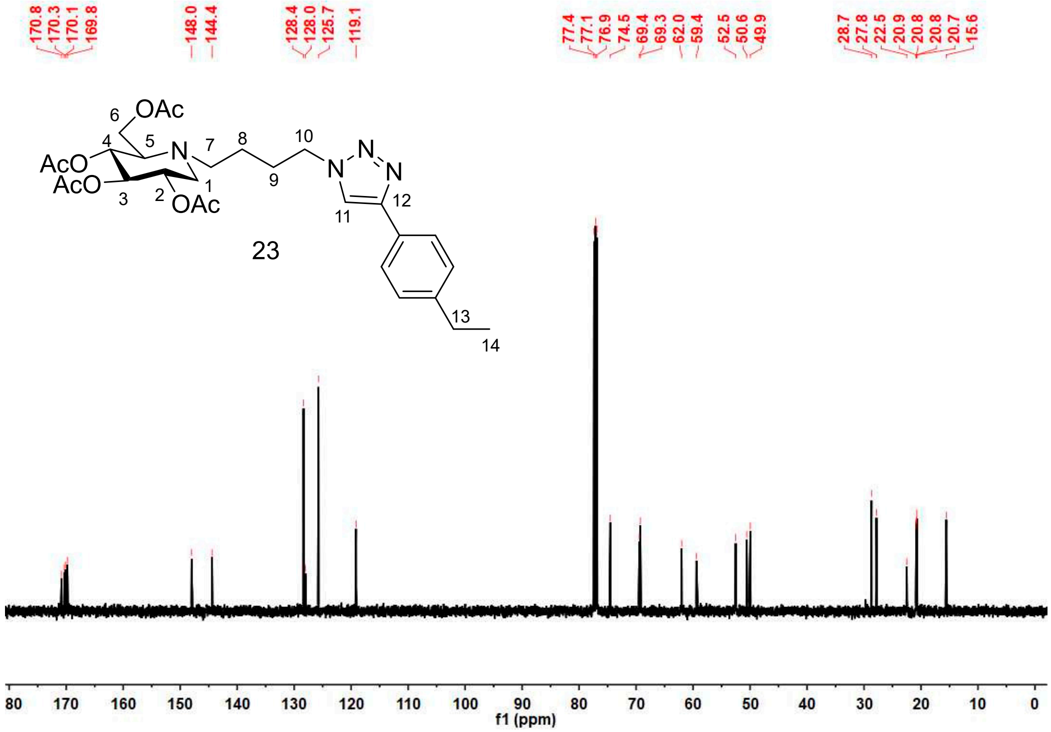

^{1}H NMR (500 MHz, $CDCl_3$) spectrum of **24**

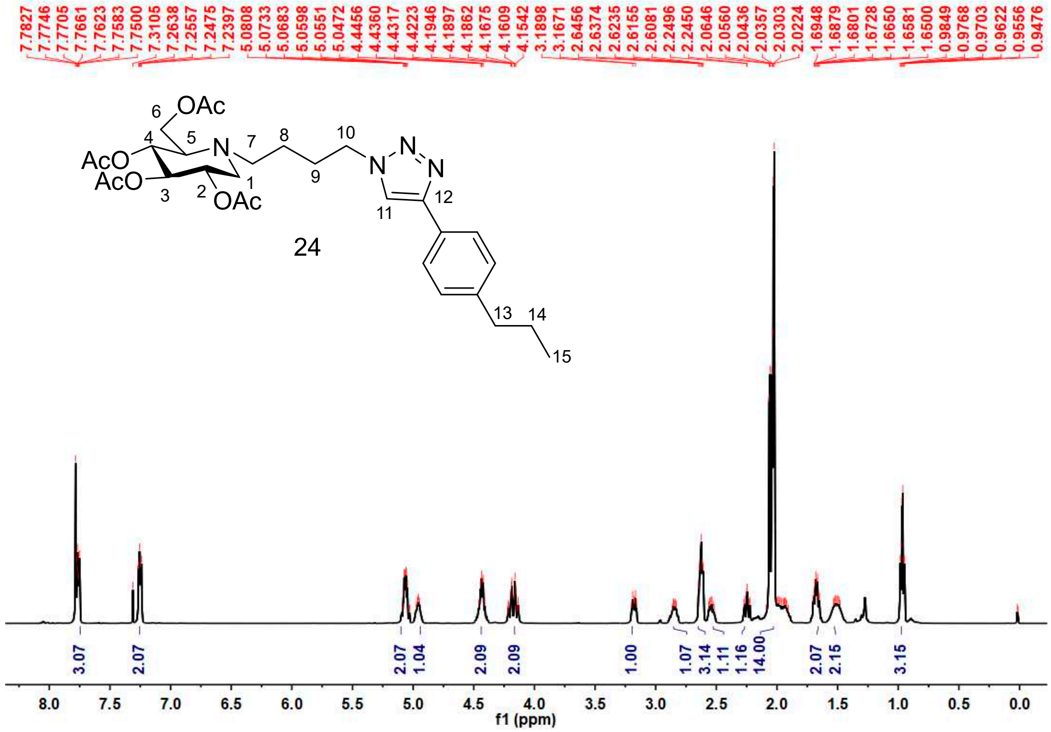

^{13}C NMR (125 MHz, $CDCl_3$) spectrum of **24**

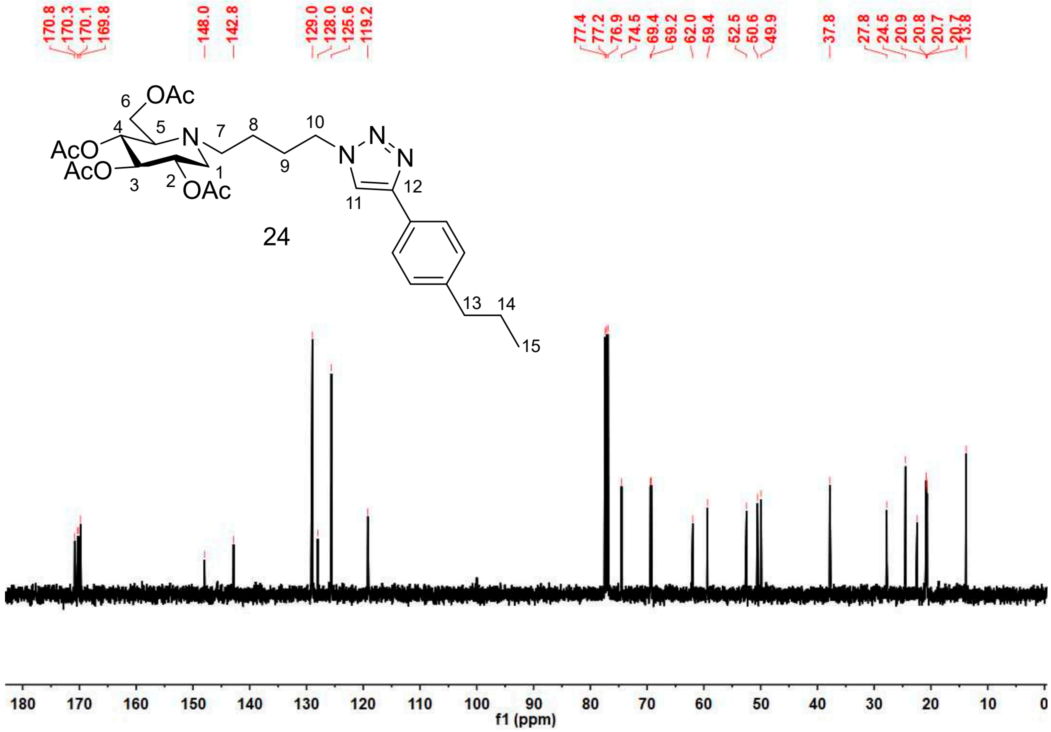

^{1}H NMR (500 MHz, $CDCl_3$) spectrum of **25**

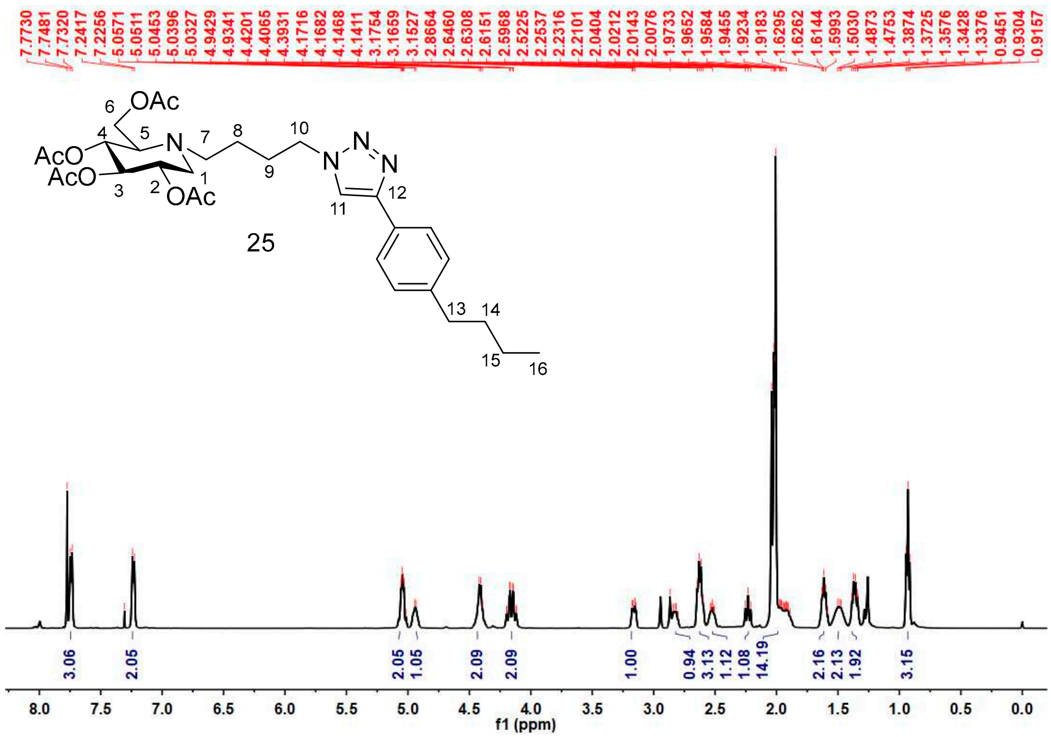

^{13}C NMR (125 MHz, $CDCl_3$) spectrum of **25**

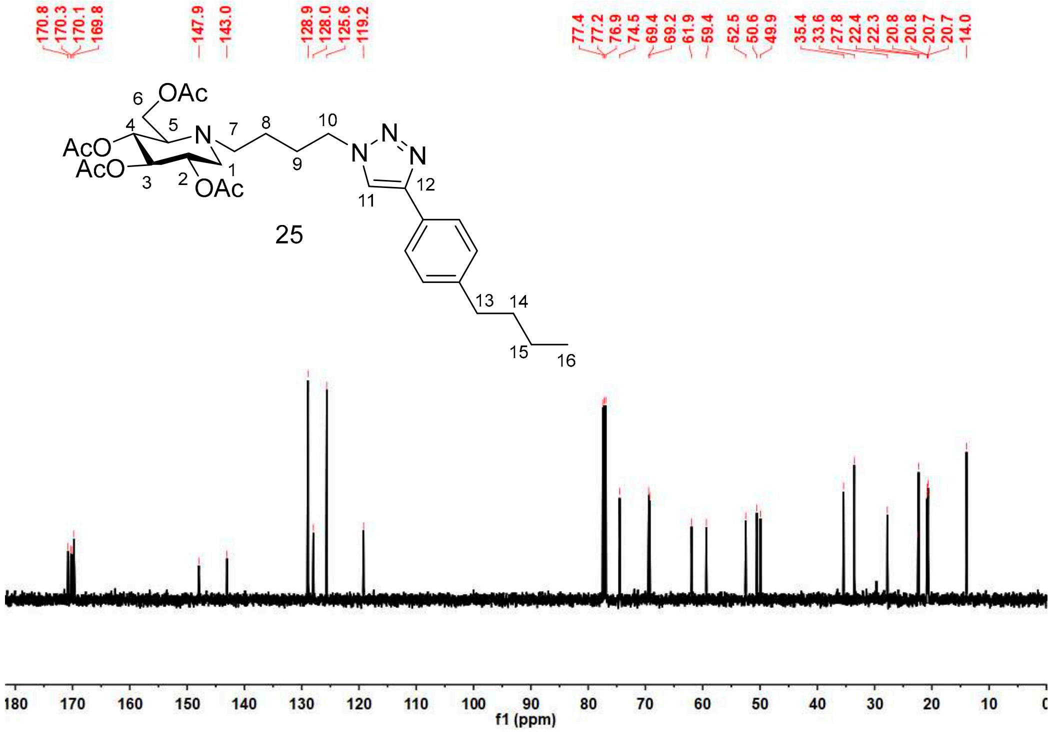

^{1}H NMR (500 MHz, $CDCl_3$) spectrum of **26**

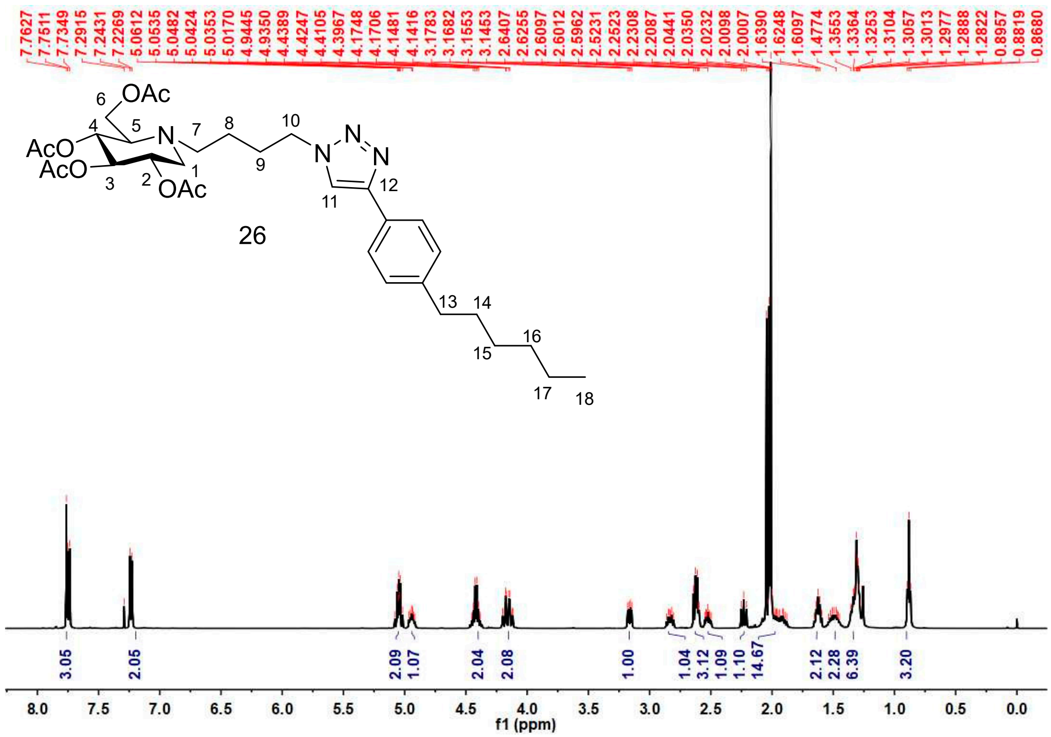

^{13}C NMR (125 MHz, $CDCl_3$) spectrum of **26**

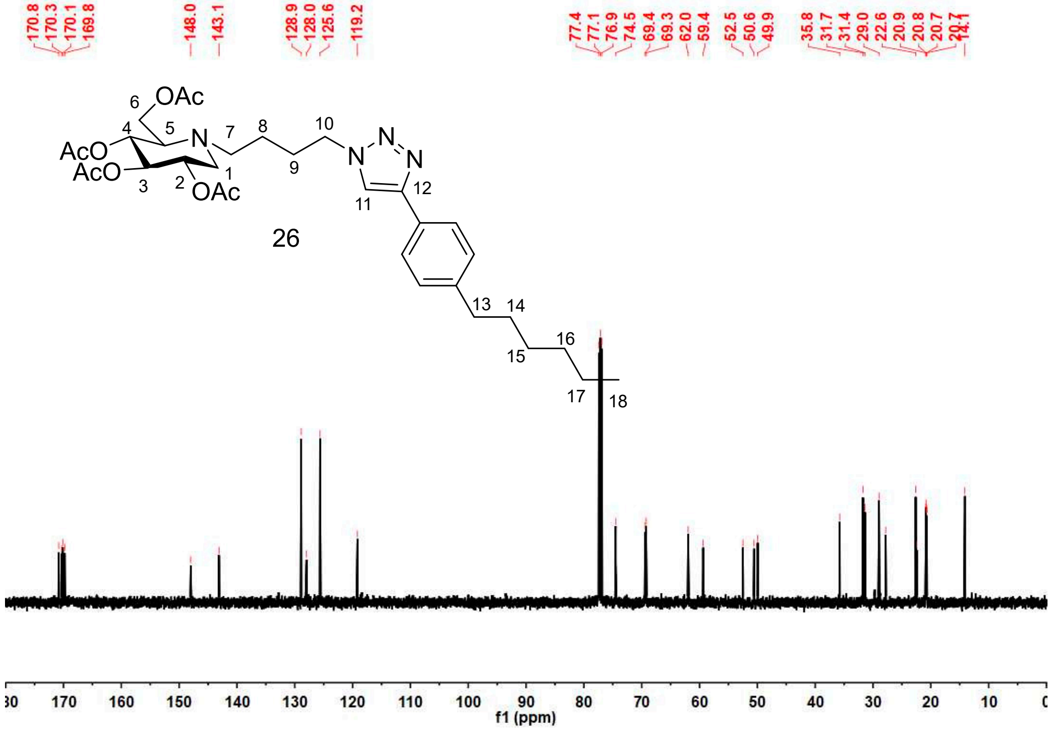

^{1}H NMR (500 MHz, $CDCl_3$) spectrum of **27**

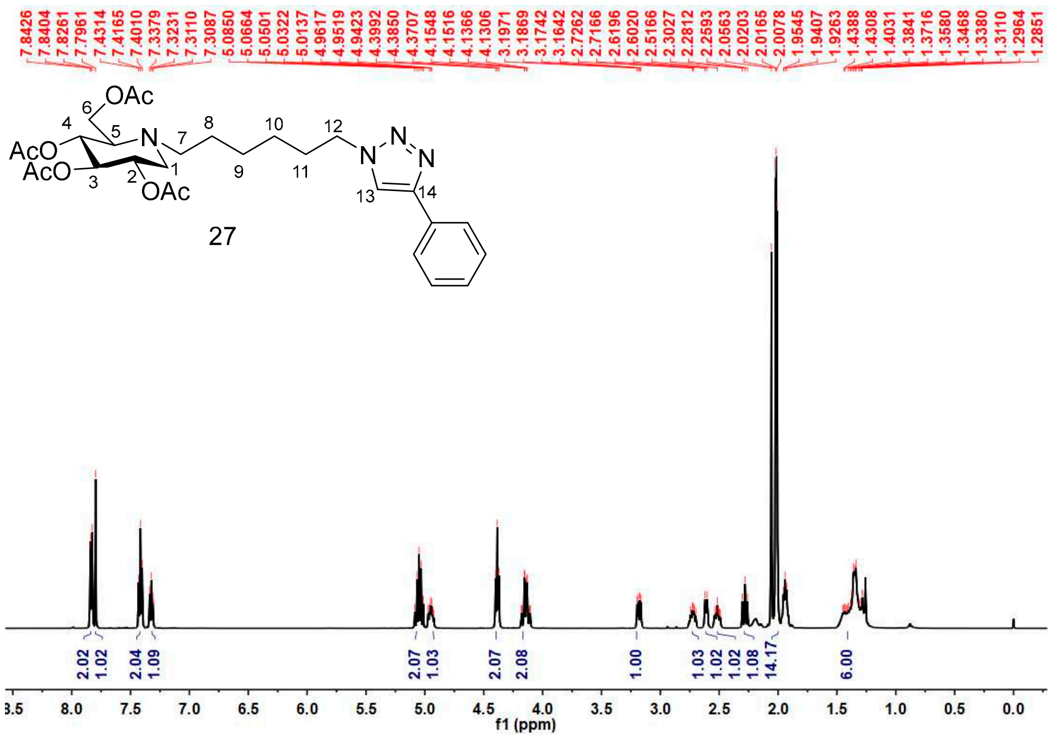

^{13}C NMR (125 MHz, $CDCl_3$) spectrum of **27**

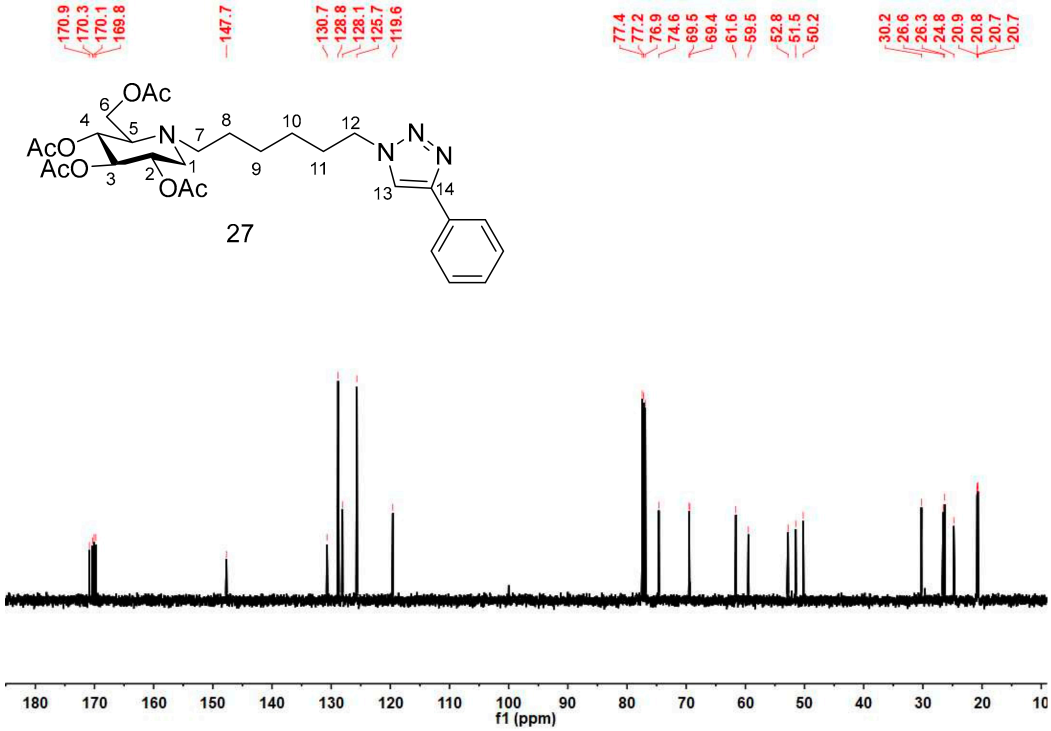

^{1}H NMR (500 MHz, $CDCl_3$) spectrum of **28**

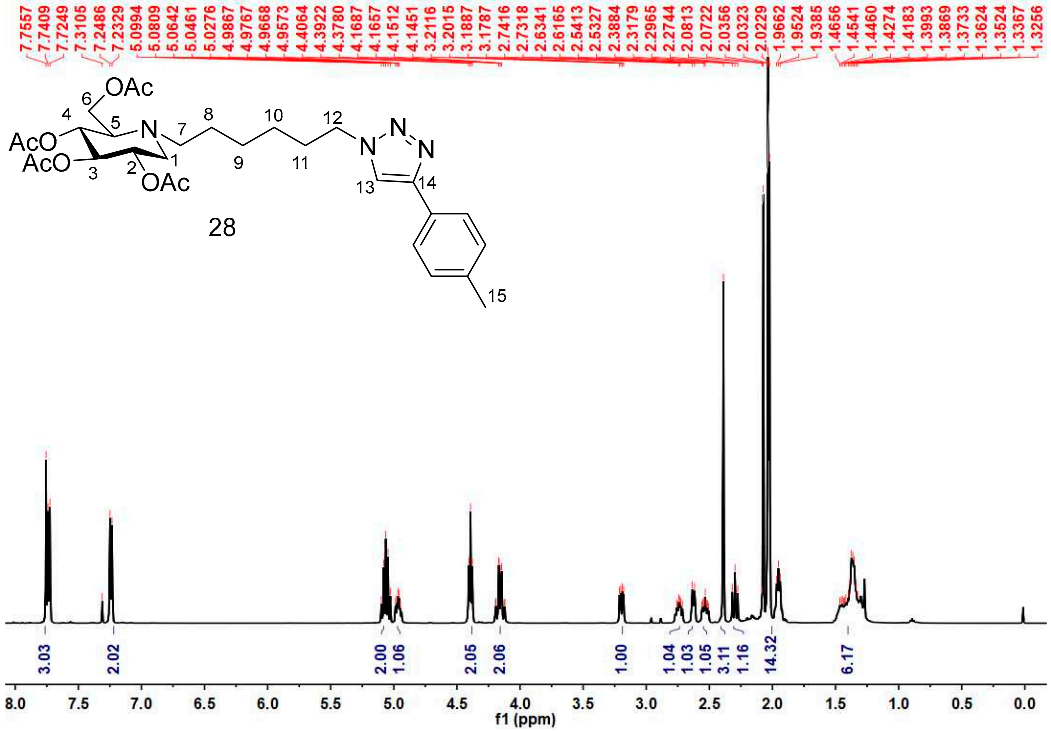

^{13}C NMR (125 MHz, $CDCl_3$) spectrum of **28**

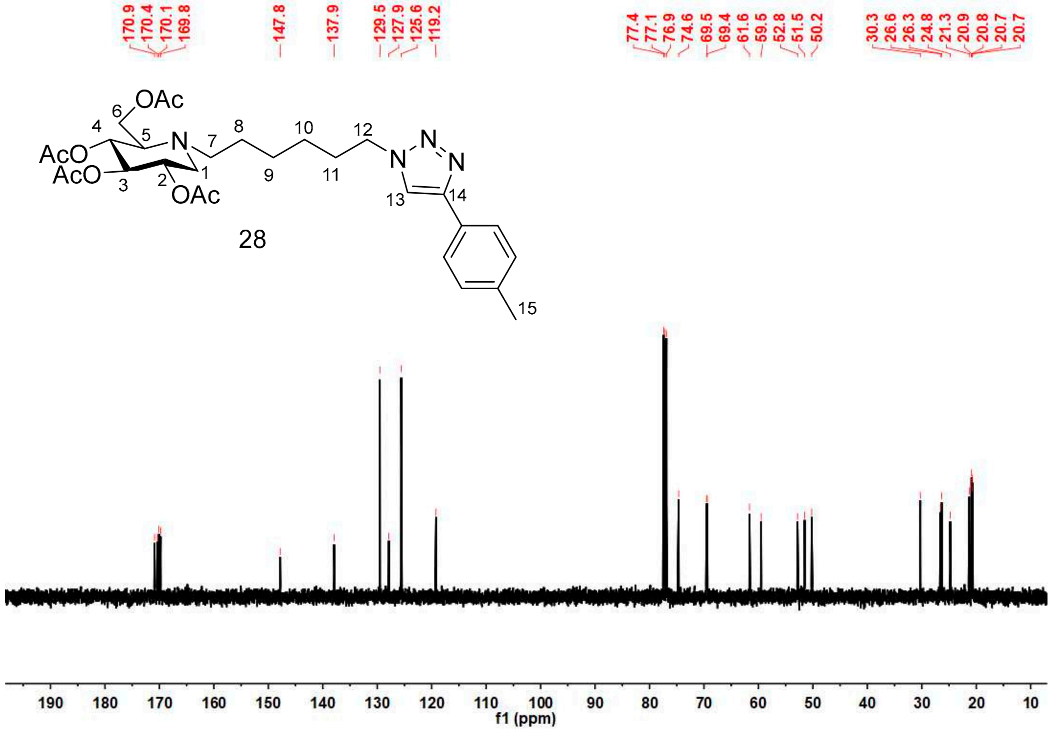

^{1}H NMR (500 MHz, $CDCl_3$) spectrum of **29**

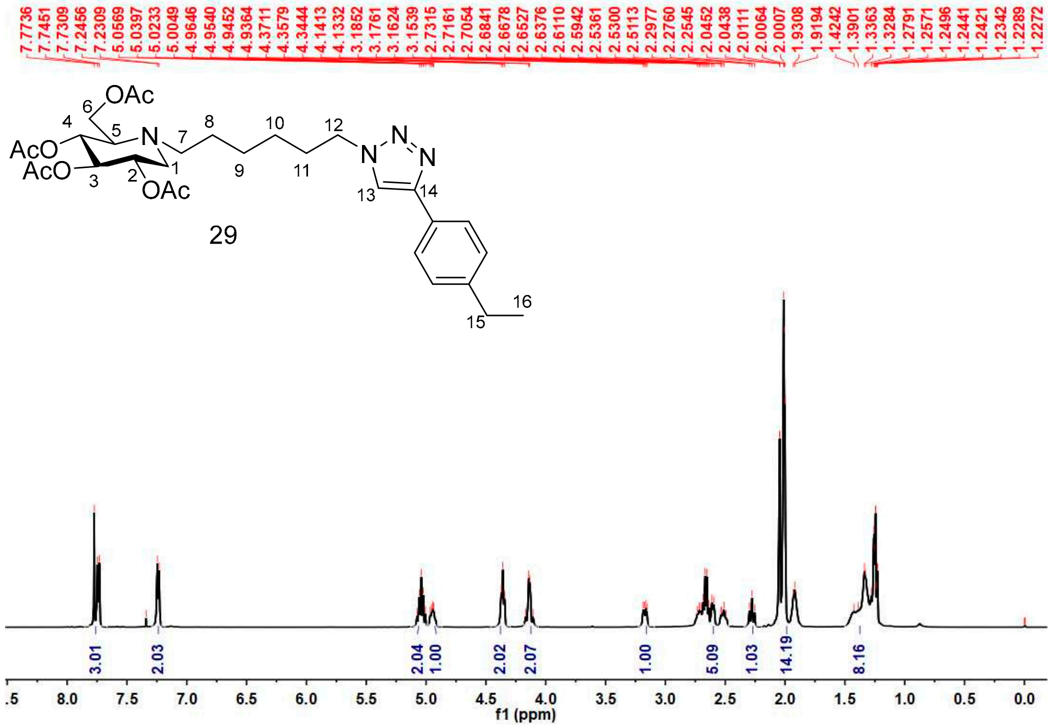

^{13}C NMR (125 MHz, $CDCl_3$) spectrum of **29**

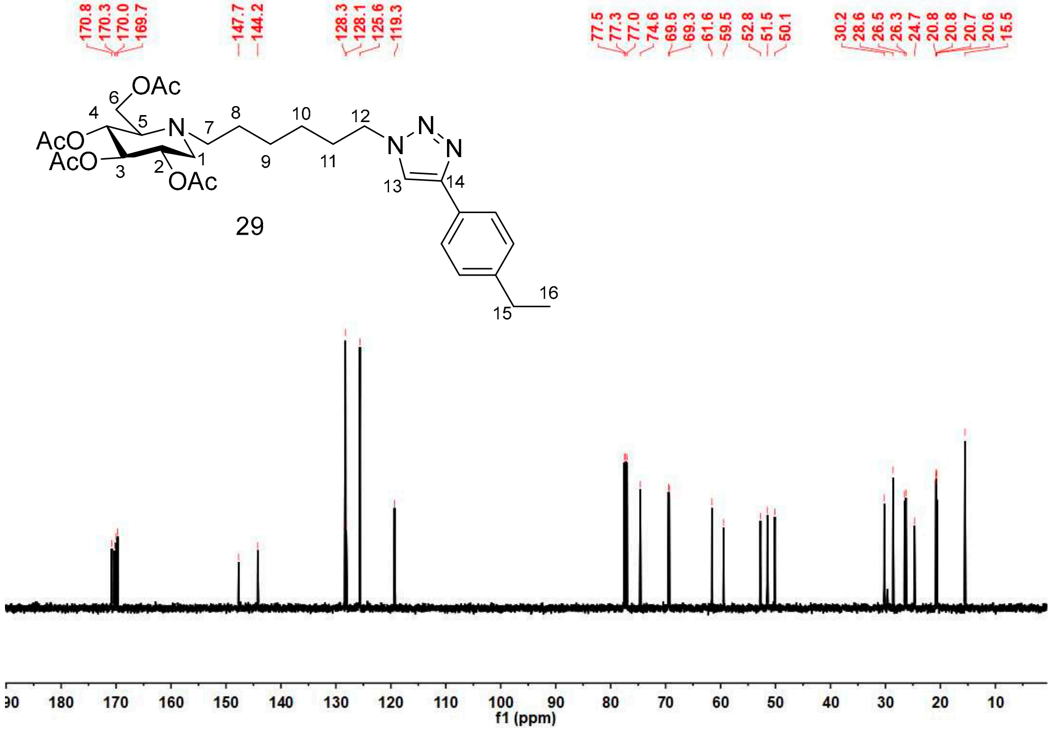

^{1}H NMR (500 MHz, $CDCl_3$) spectrum of **30**

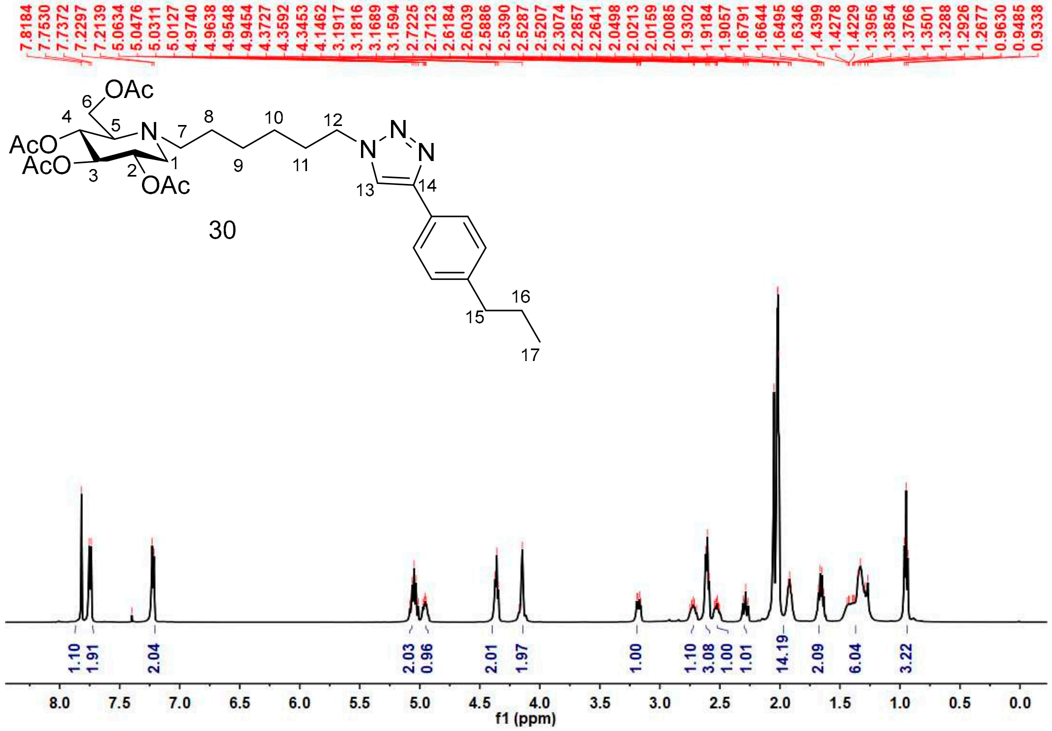

^{13}C NMR (125 MHz, $CDCl_3$) spectrum of **30**

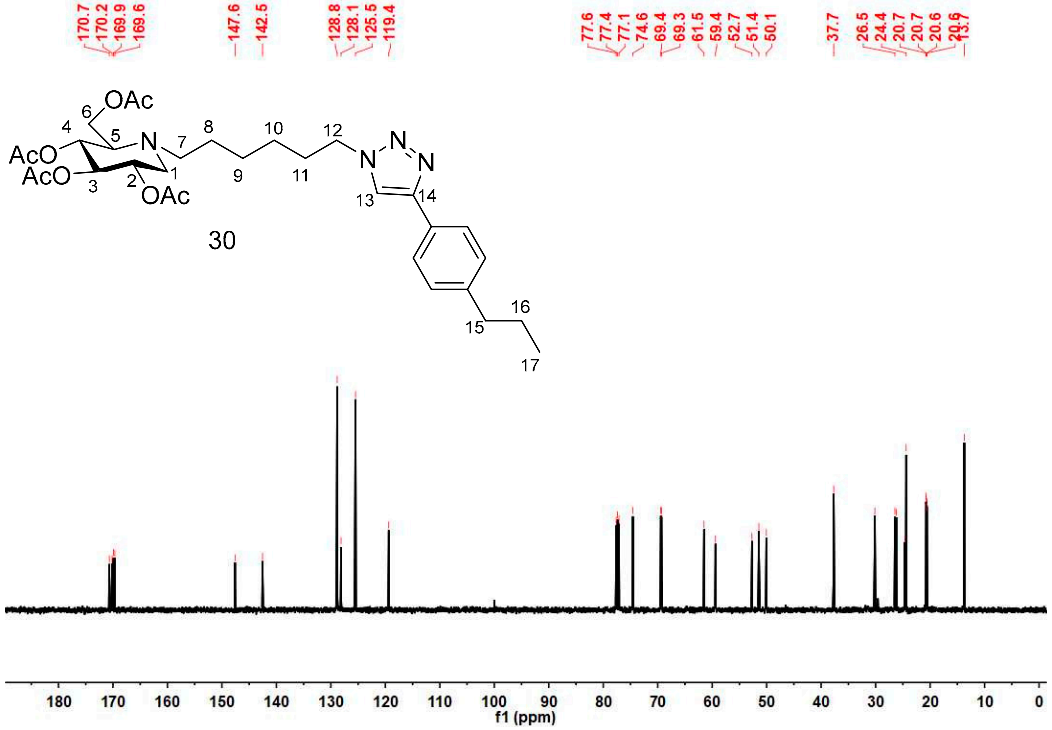

^{1}H NMR (500 MHz, $CDCl_3$) spectrum of **31**

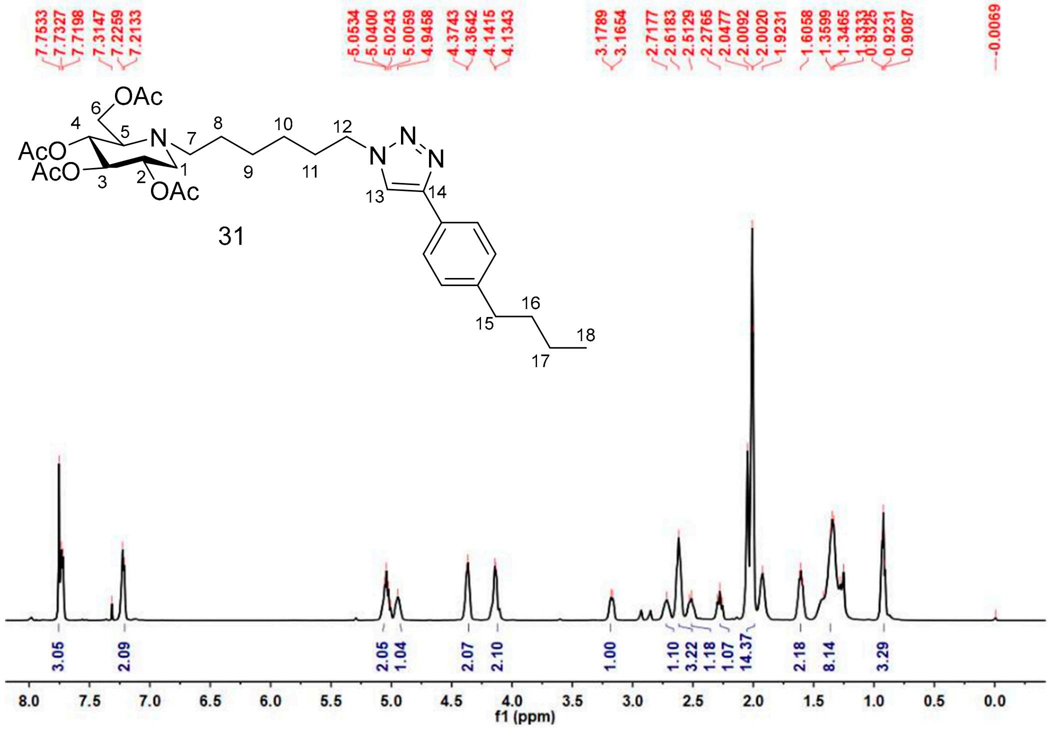

^{13}C NMR (125 MHz, $CDCl_3$) spectrum of **31**

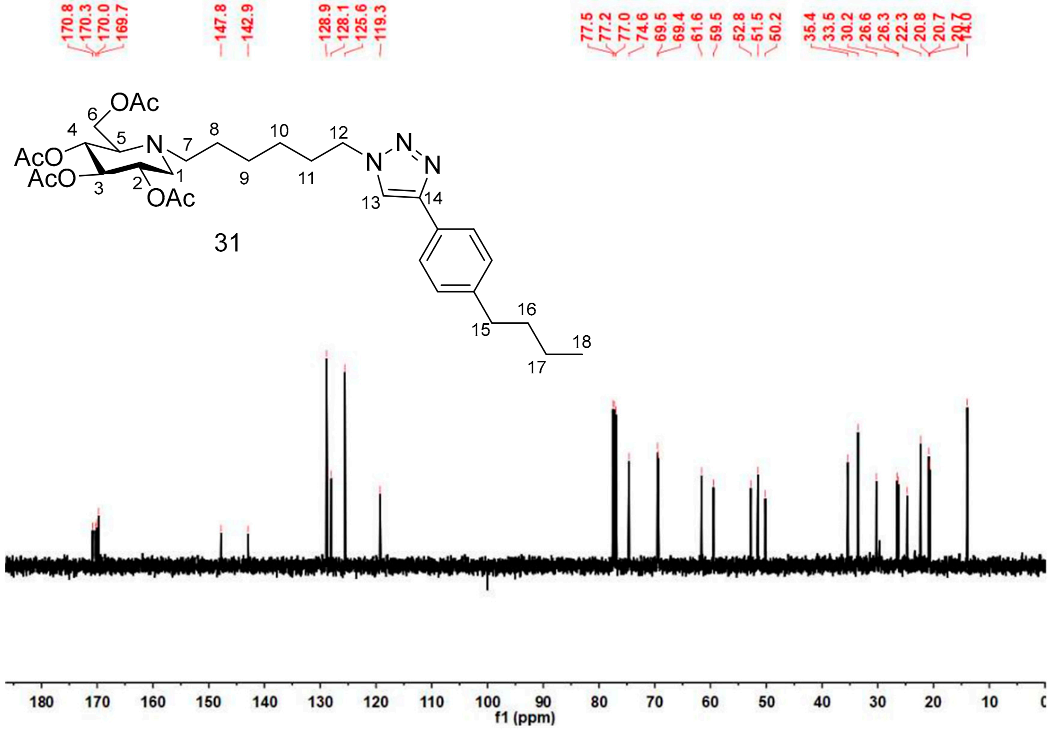

^{1}H NMR (500 MHz, CDCl$_3$) spectrum of **32**

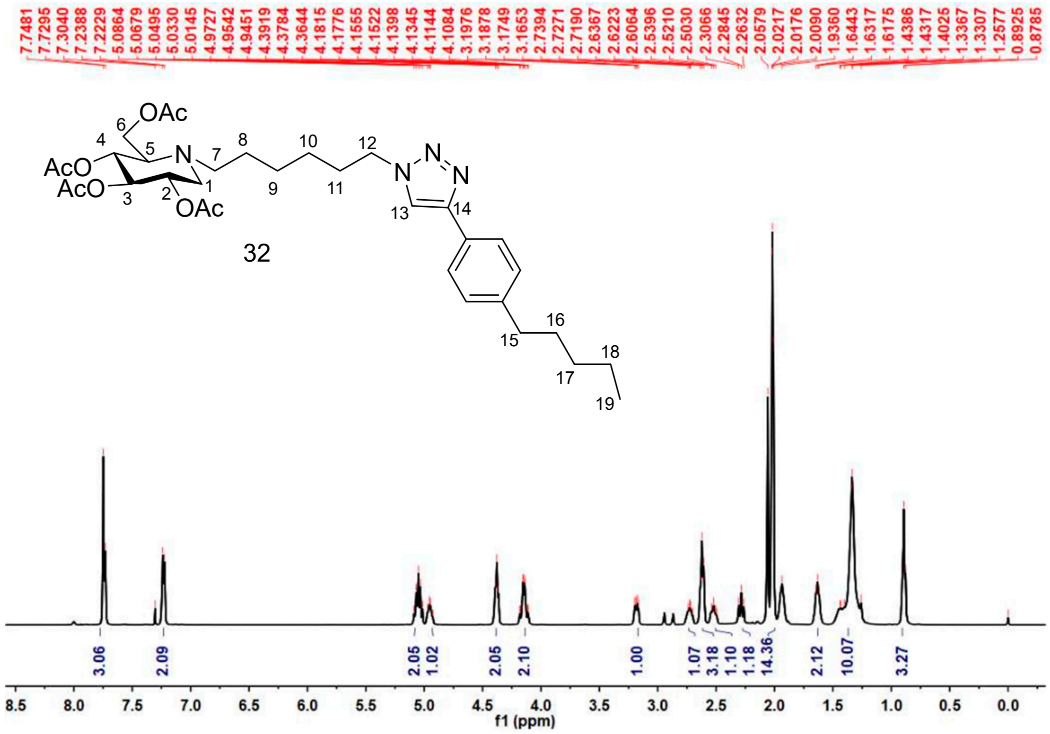

^{13}C NMR (125 MHz, CDCl$_3$) spectrum of **32**

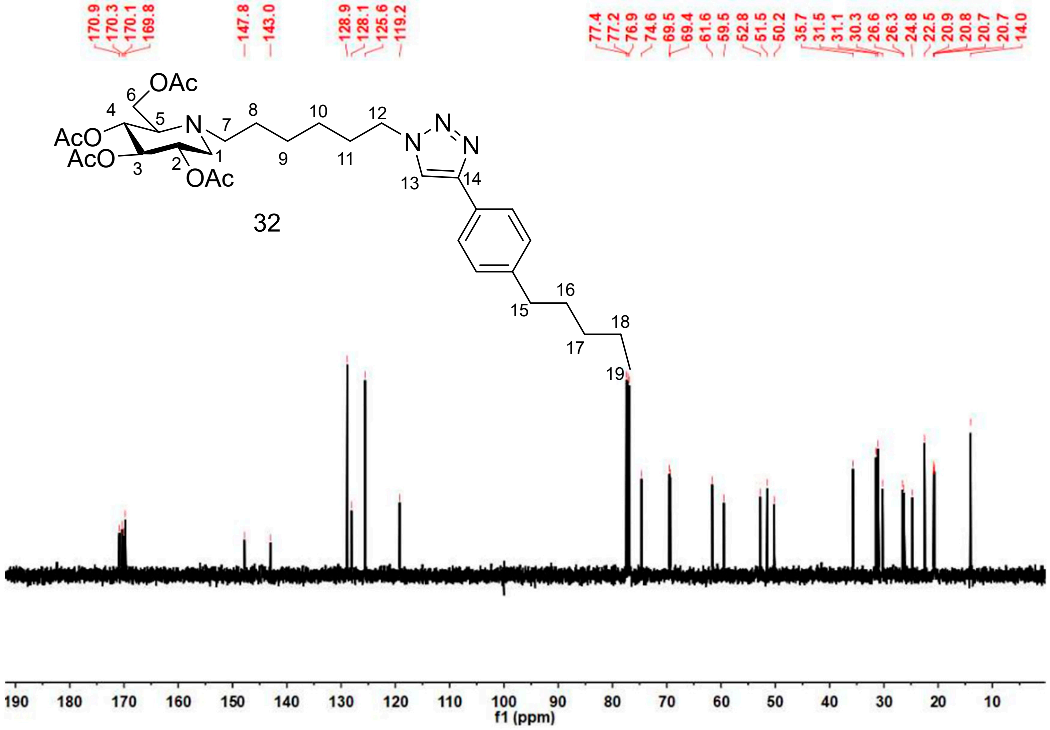

^{1}H NMR (500 MHz, CDCl$_3$) spectrum of **33**

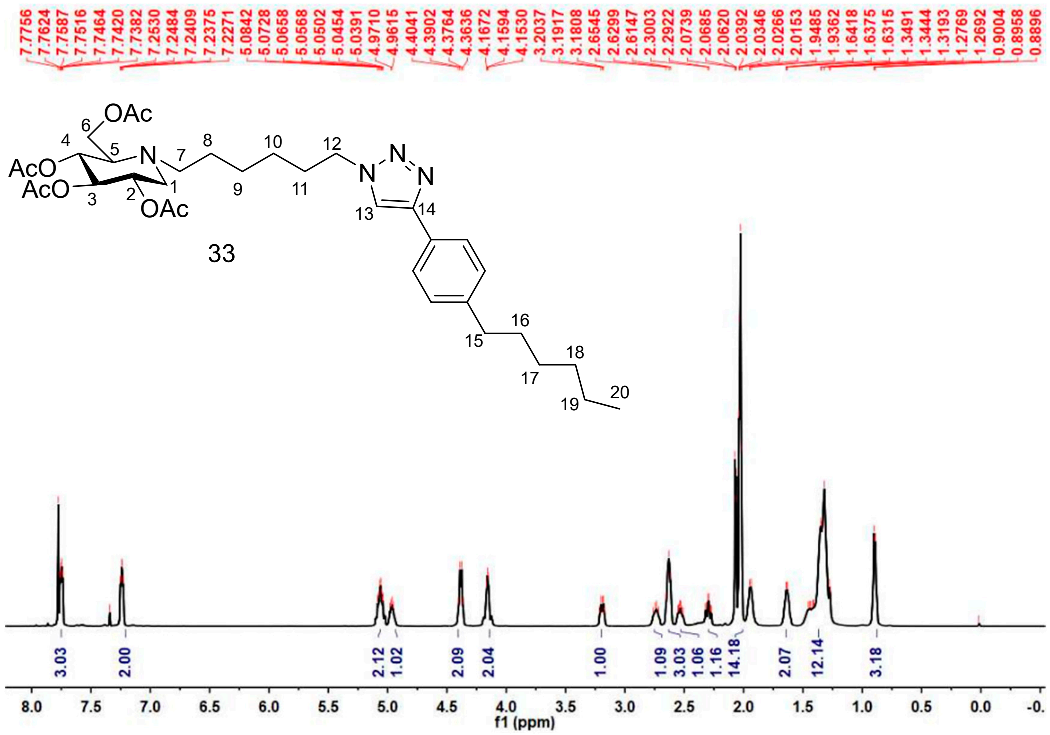

^{13}C NMR (125 MHz, $CDCl_3$) spectrum of **33**

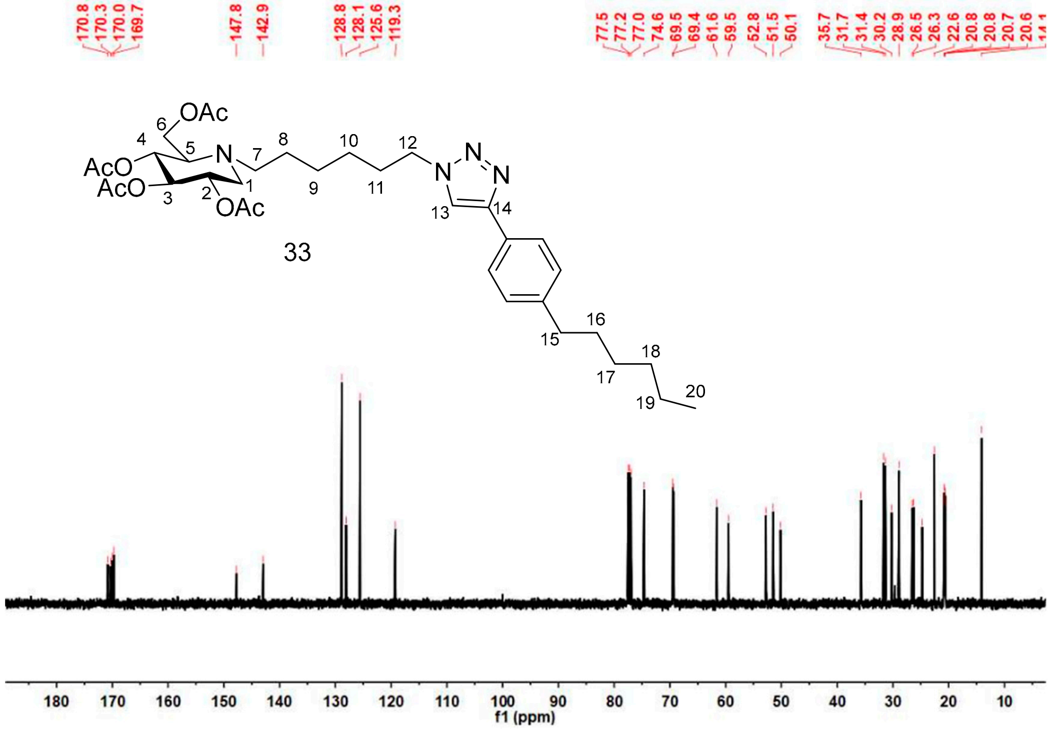

^{1}H NMR (500 MHz, D_2O) spectrum of **10**

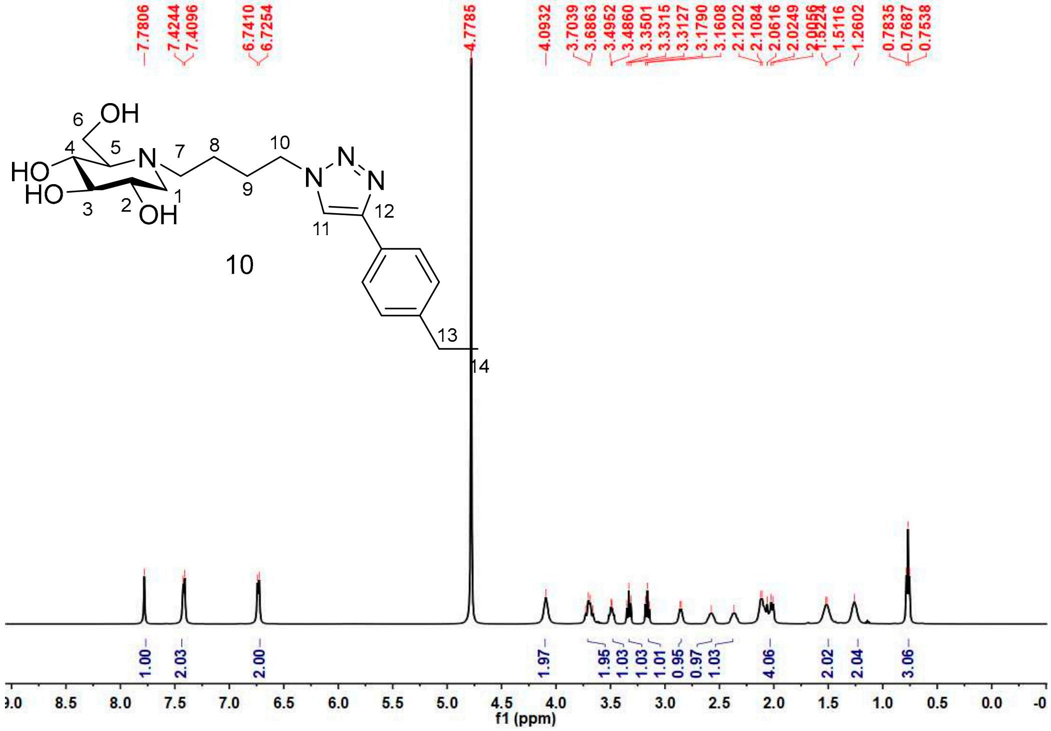

^{13}C NMR (125 MHz, D_2O) spectrum of **10**

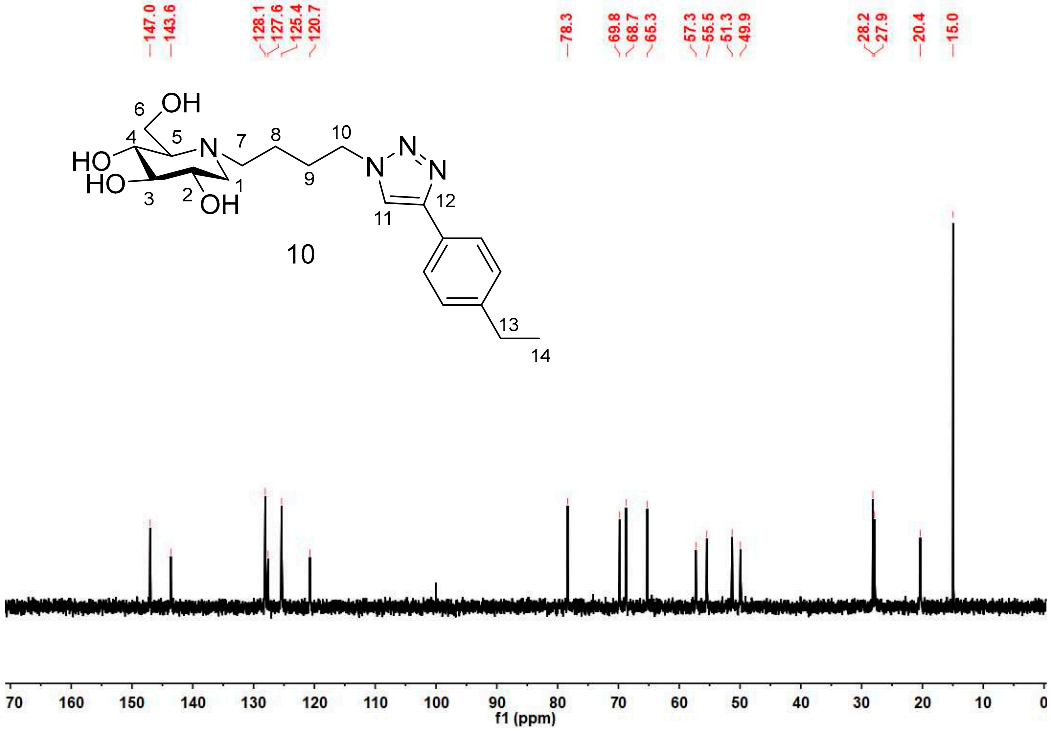

HRMS spectrum of **10**

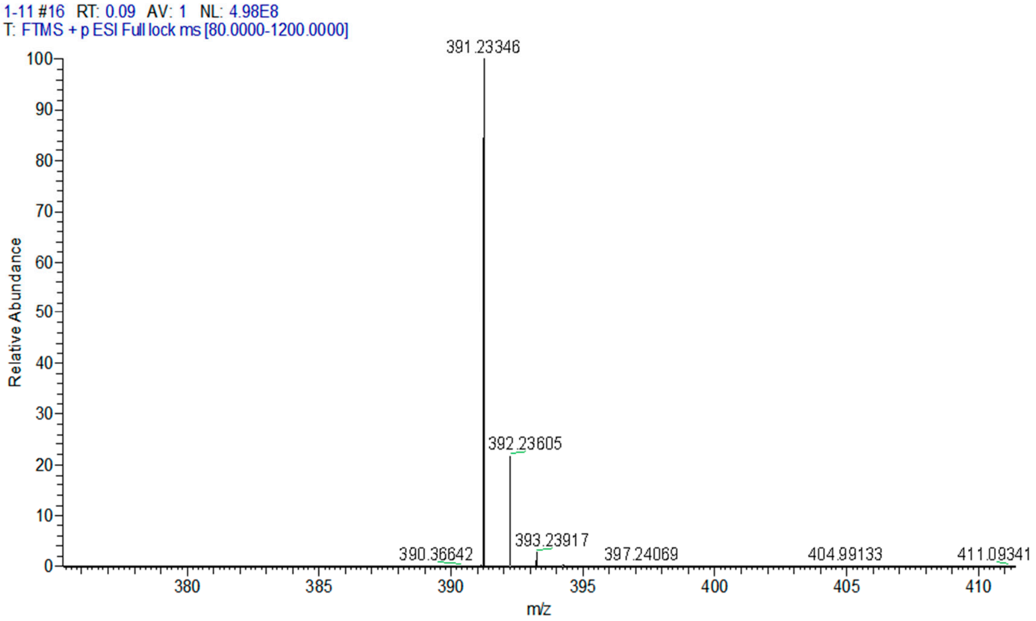

^{1}H NMR (500 MHz, D_2O) spectrum of **11**

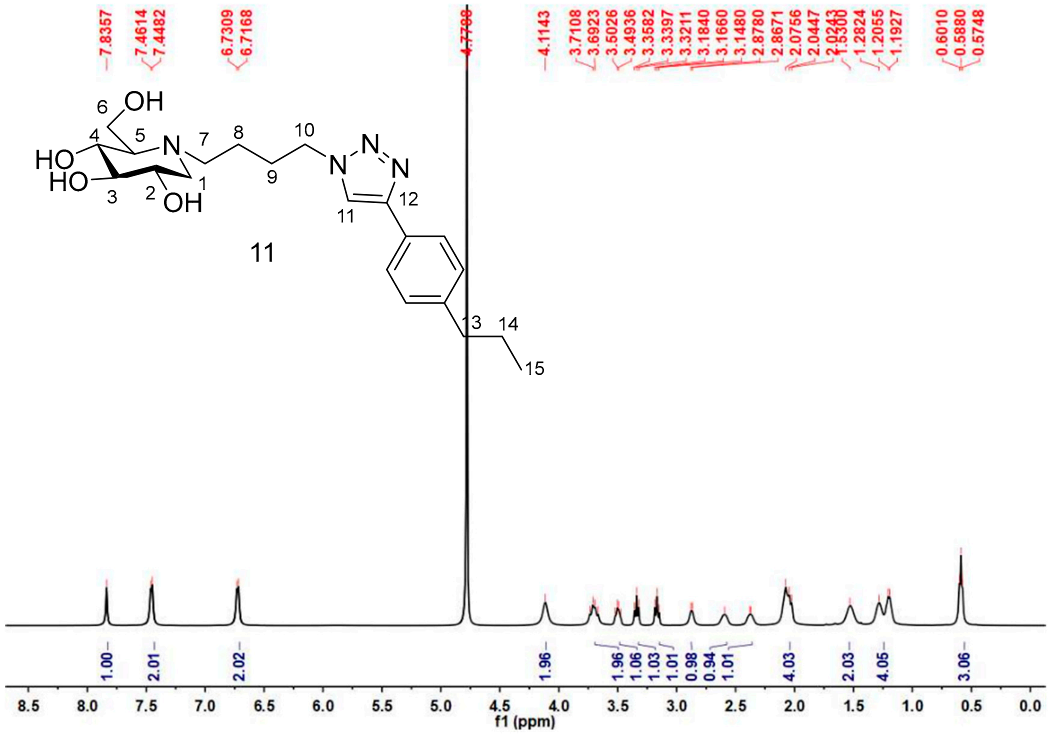

^{13}C NMR (125 MHz, D_2O) spectrum of **11**

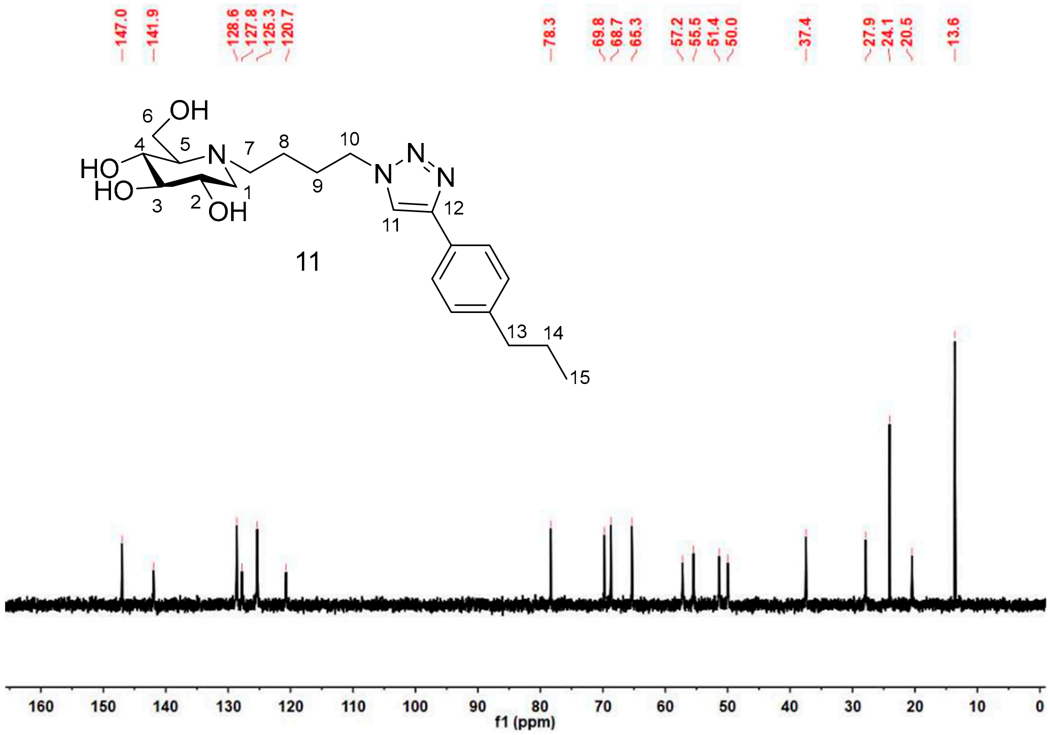

HRMS spectrum of **11**

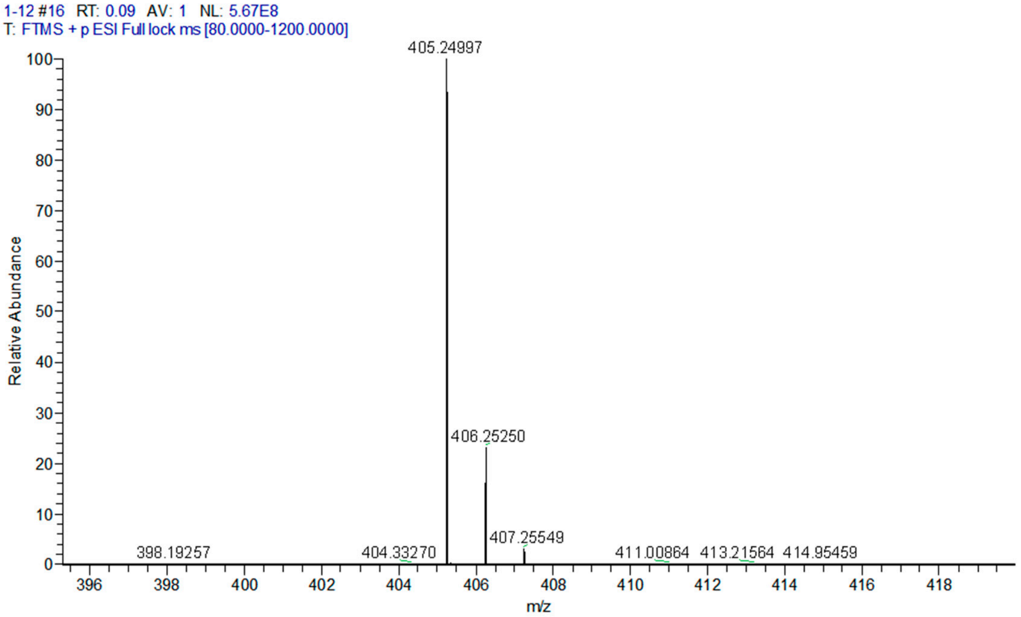

^{1}H NMR (500 MHz, D_2O) spectrum of **12**

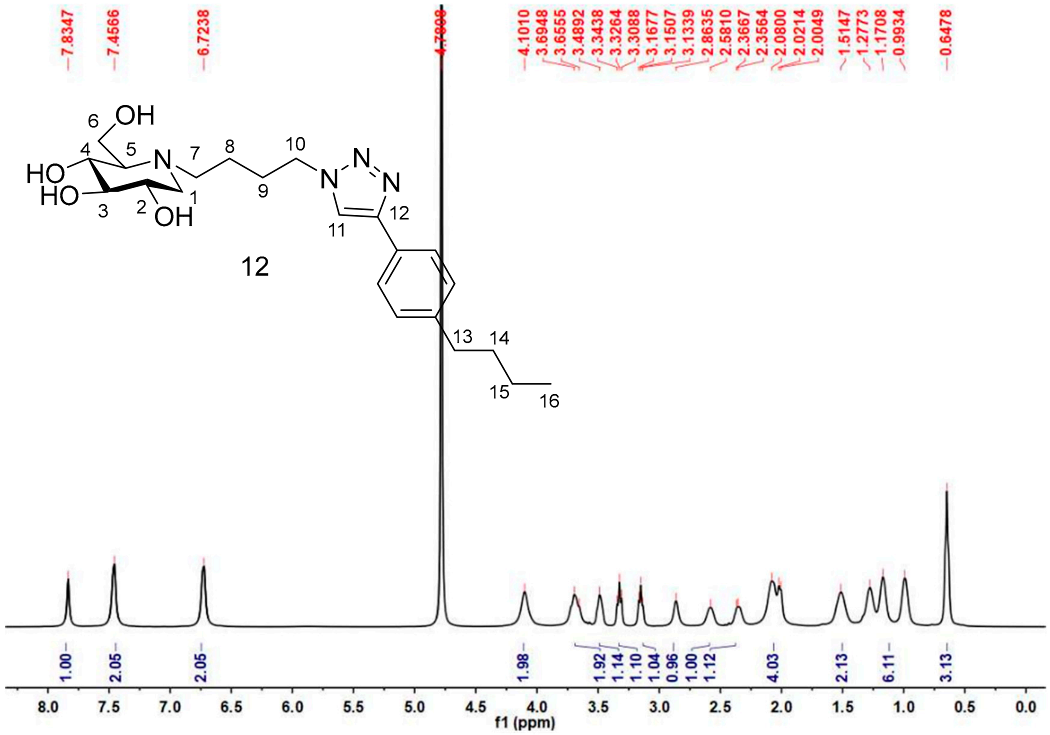

^{13}C NMR (125 MHz, D_2O) spectrum of **12**

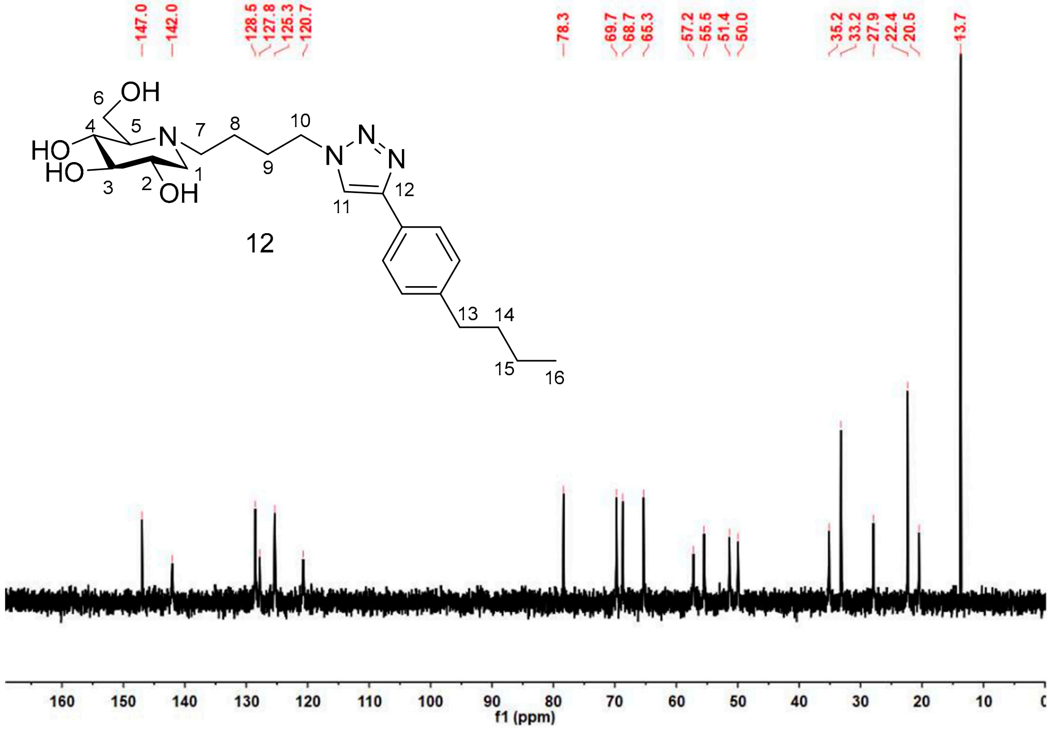

HRMS spectrum of **12**

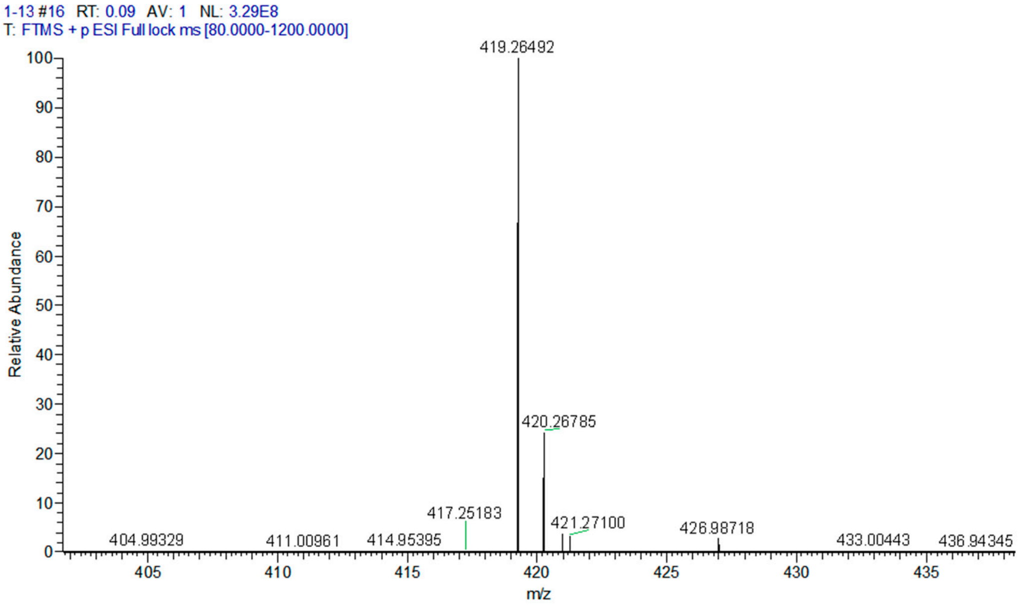

^{1}H NMR (500 MHz, D_2O) spectrum of **13**

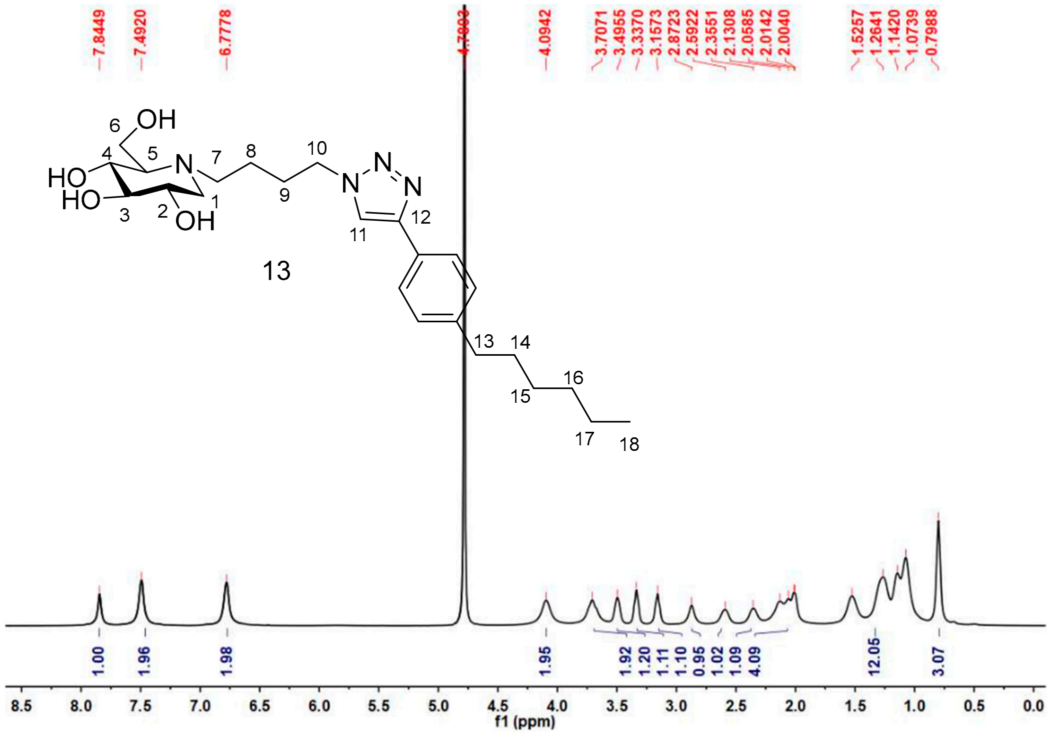

^{13}C NMR (125 MHz, D_2O) spectrum of **13**

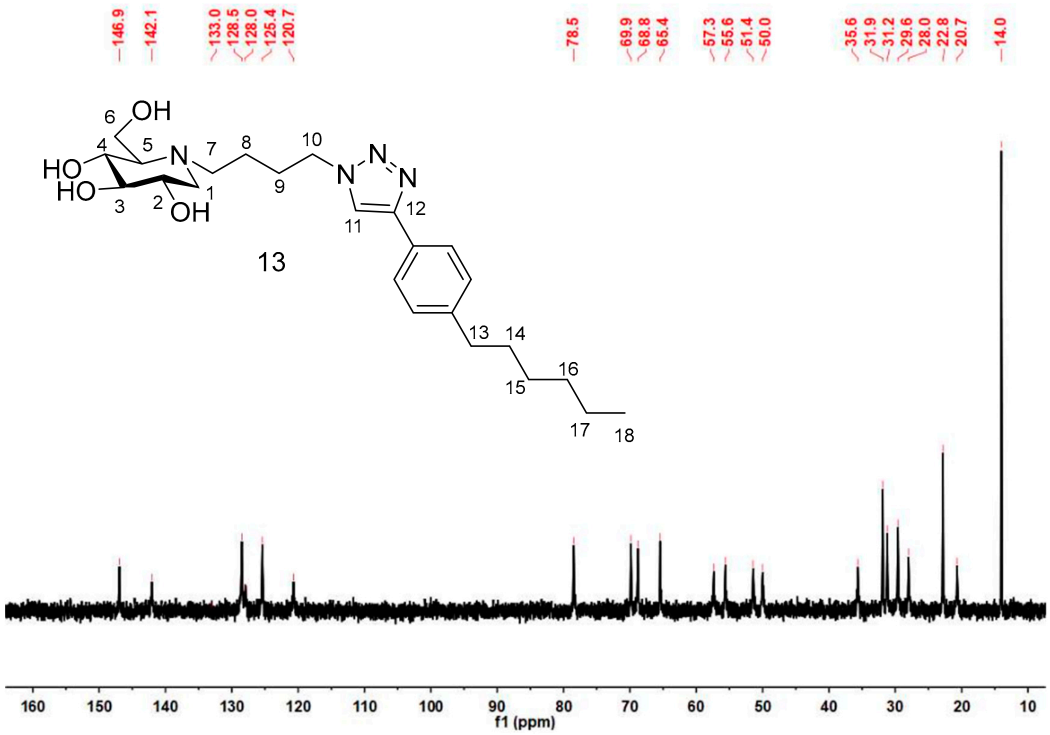

HRMS spectrum of **13**

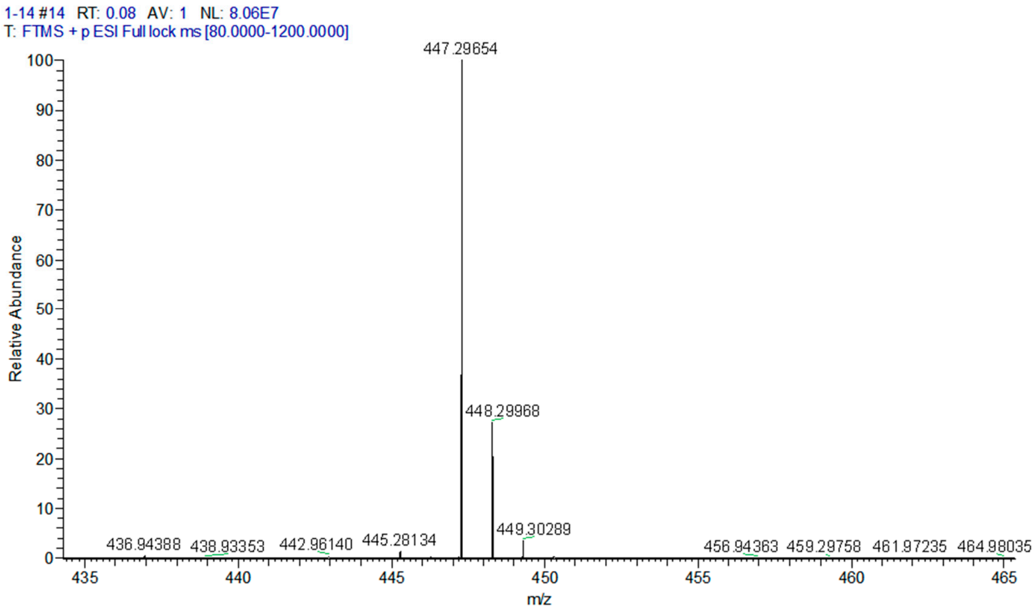

^{1}H NMR (500 MHz, CD3OD_SPE) spectrum of **14**

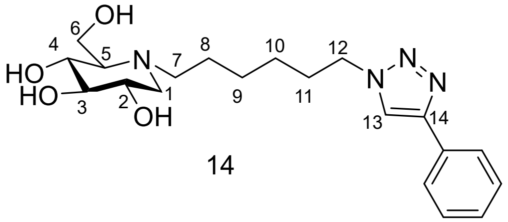

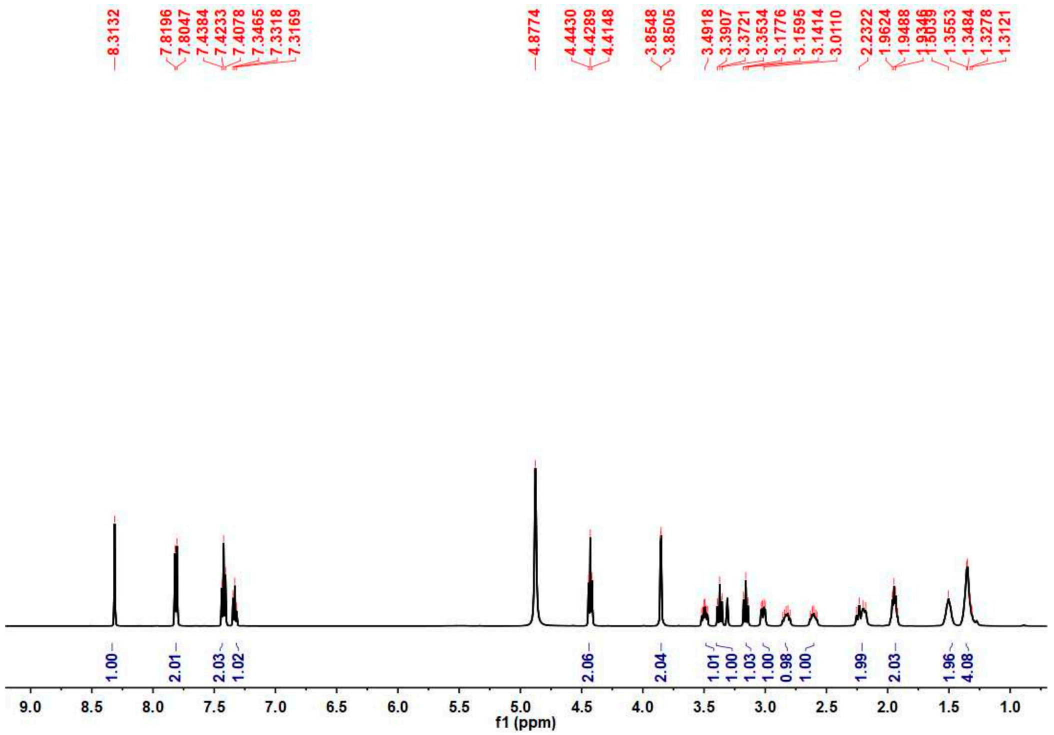

^{13}C NMR (125 MHz, CD_3OD_SPE) spectrum of **14**

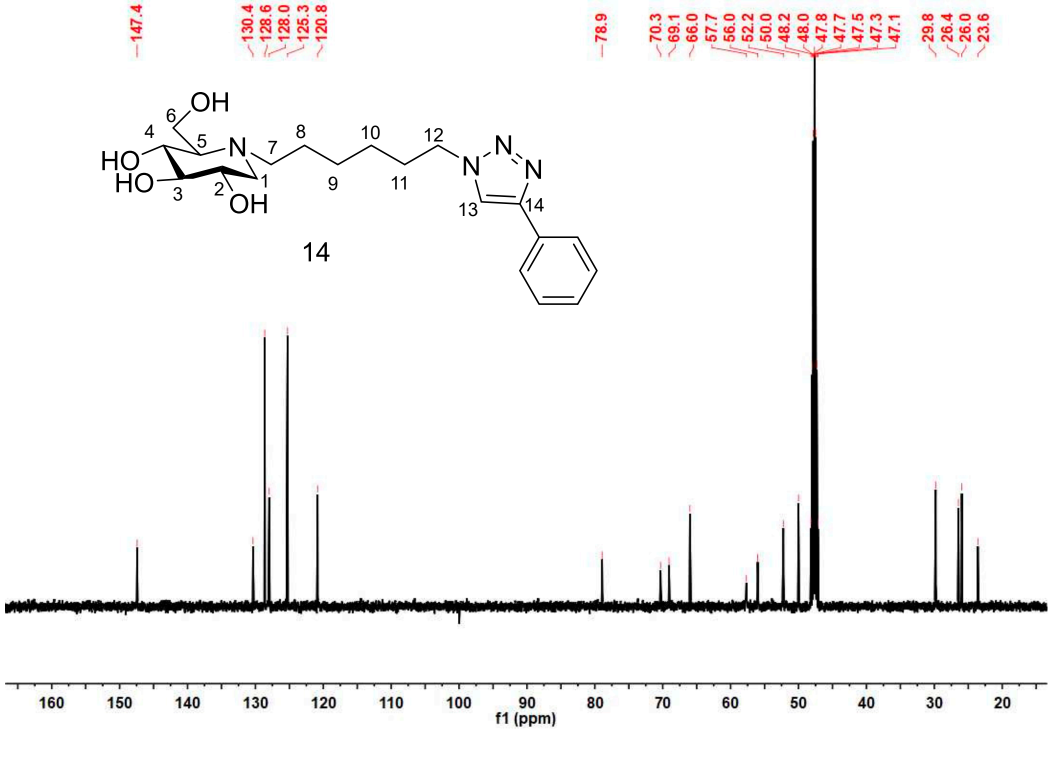

HRMS spectrum of **14**

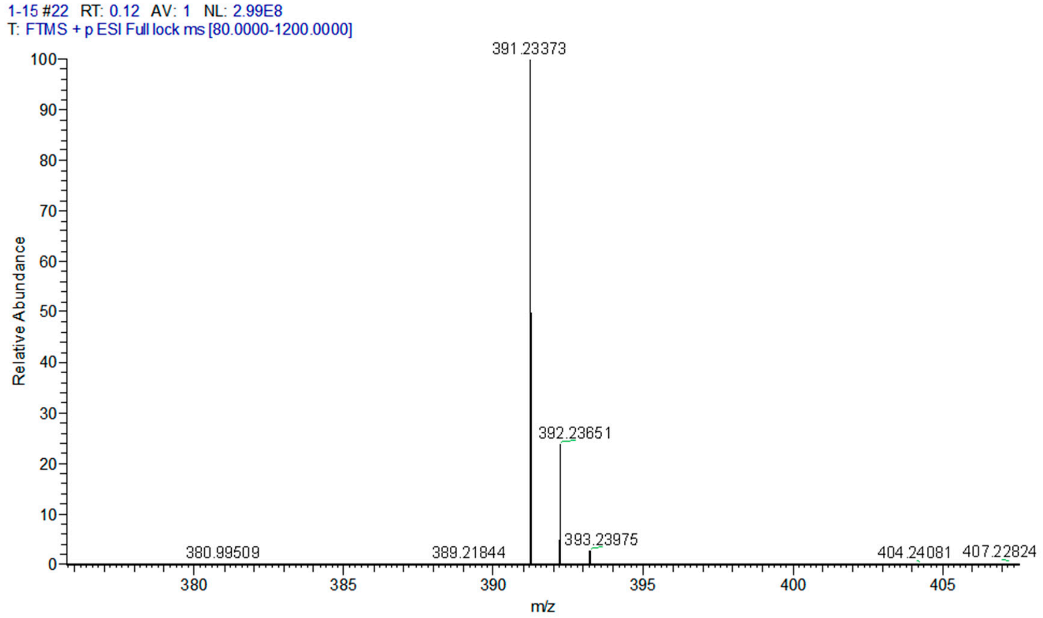

^{1}H NMR (500 MHz, CD$_3$OD_SPE) spectrum of **15**

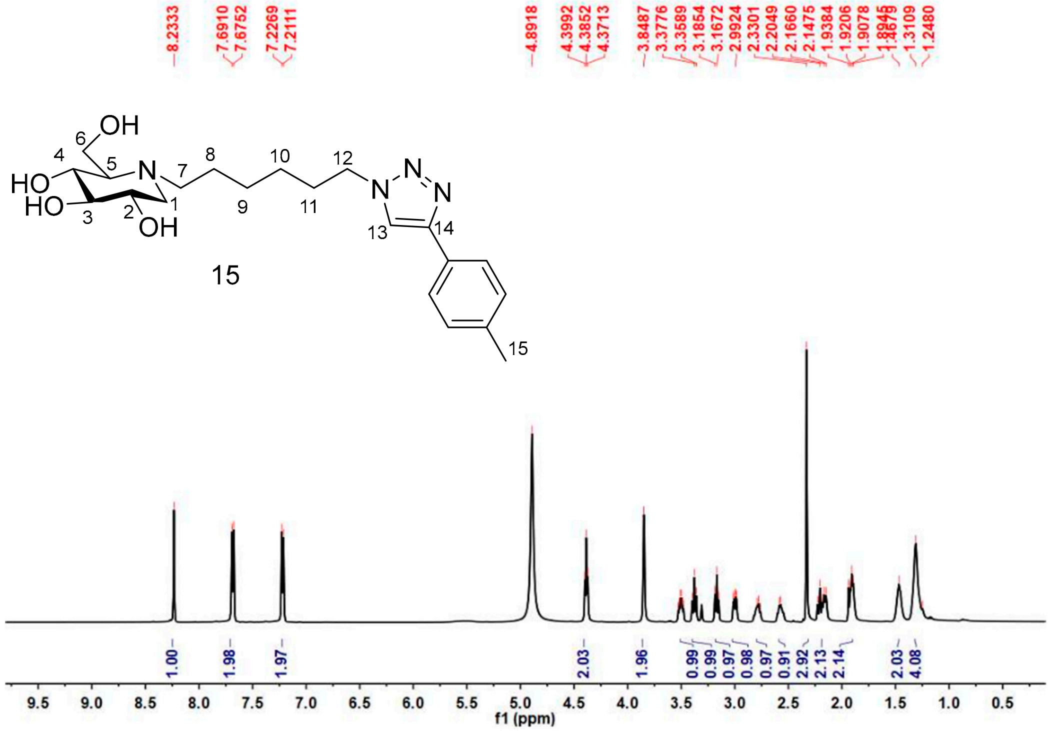

^{13}C NMR (125 MHz, CD$_3$OD_SPE) spectrum of **15**

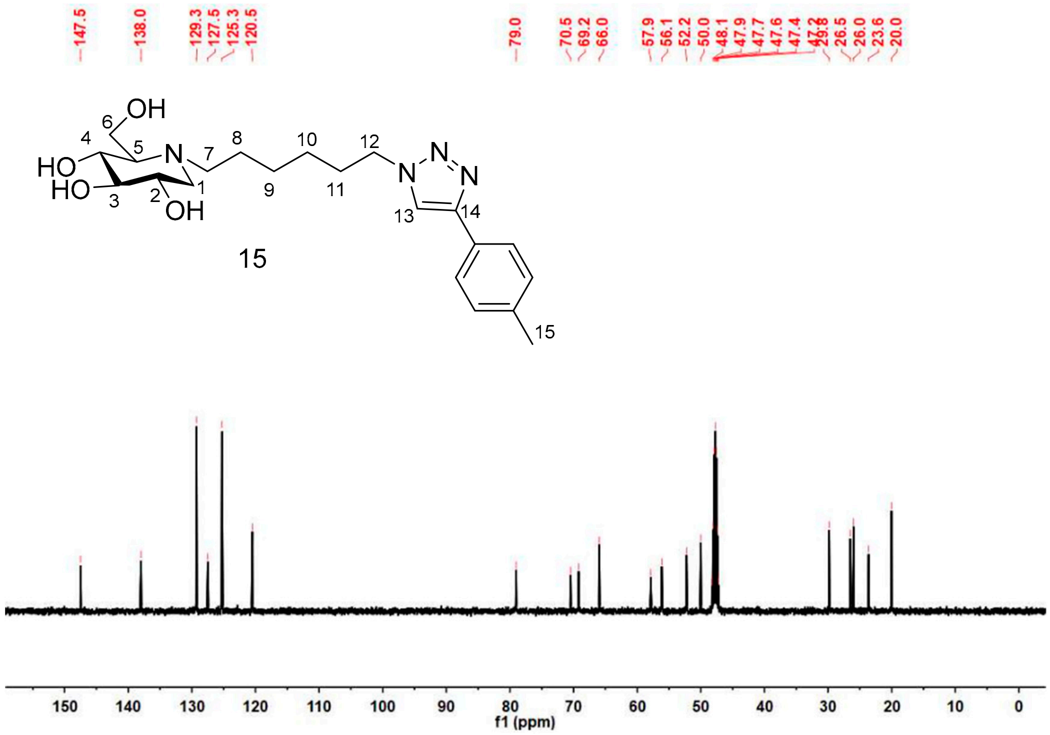

HRMS spectrum of **15**

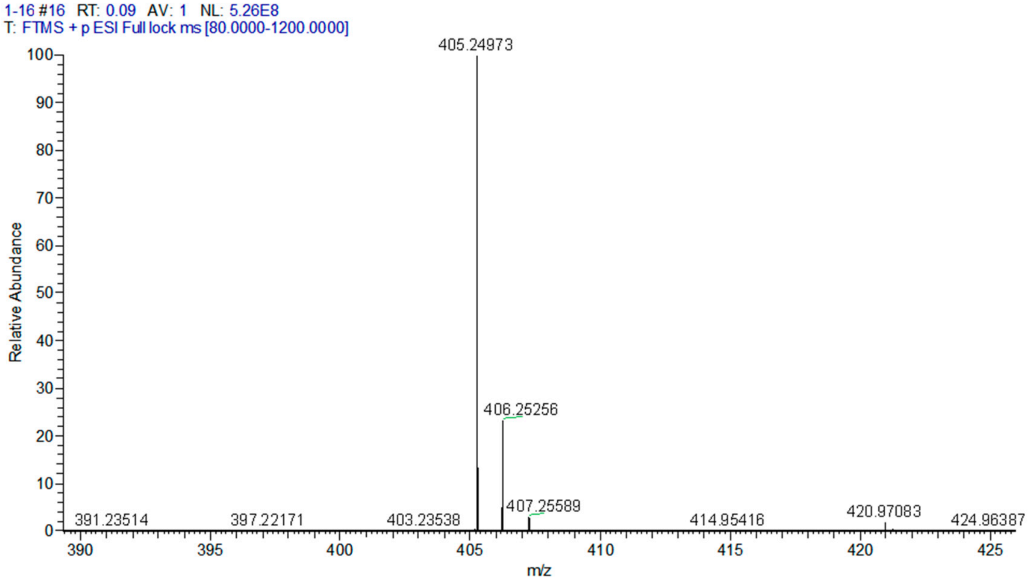

^{1}H NMR (500 MHz, D_2O) spectrum of **16**

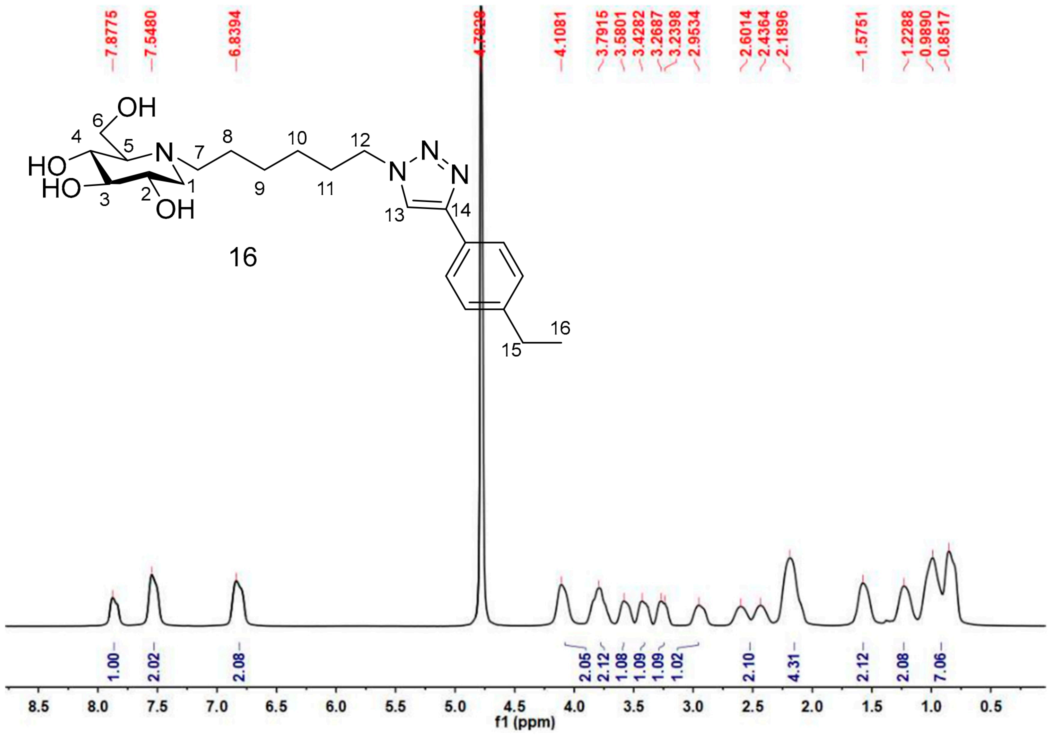

^{13}C NMR (125 MHz, D_2O) spectrum of **16**

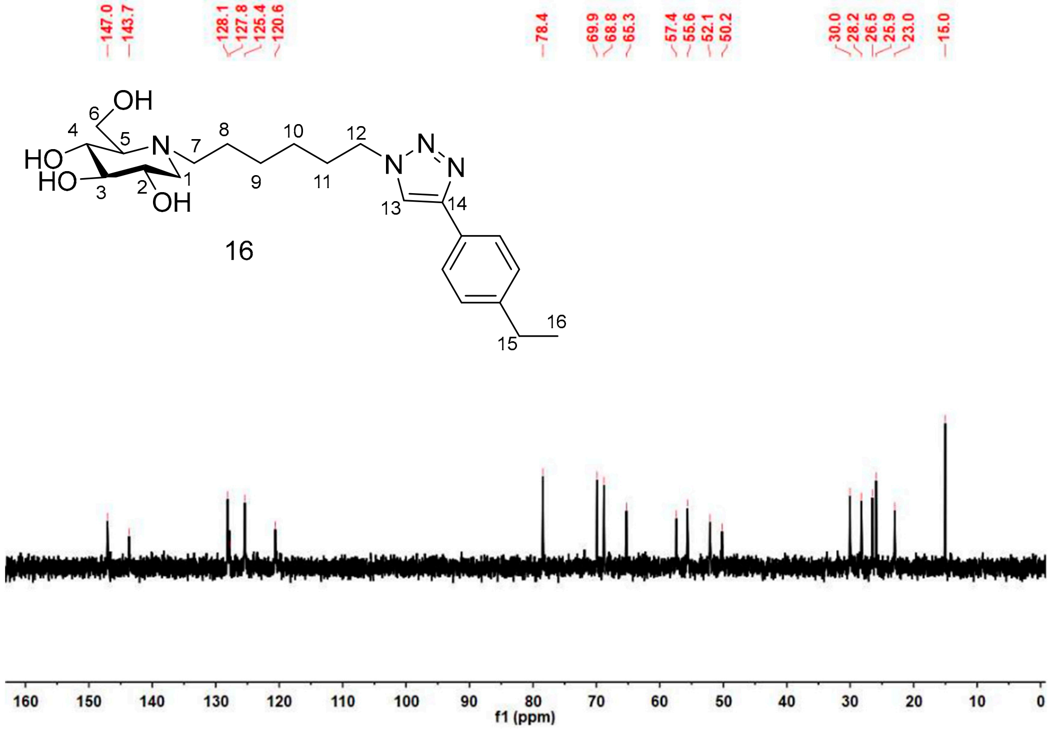

HRMS spectrum of **16**

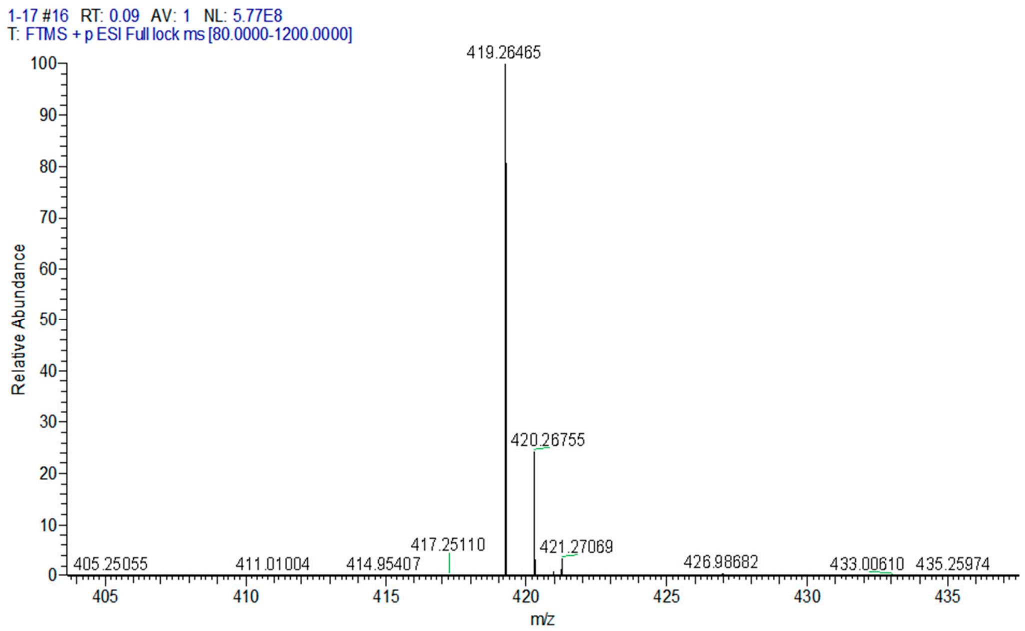

^{1}H NMR (500 MHz, D_2O) spectrum of **17**

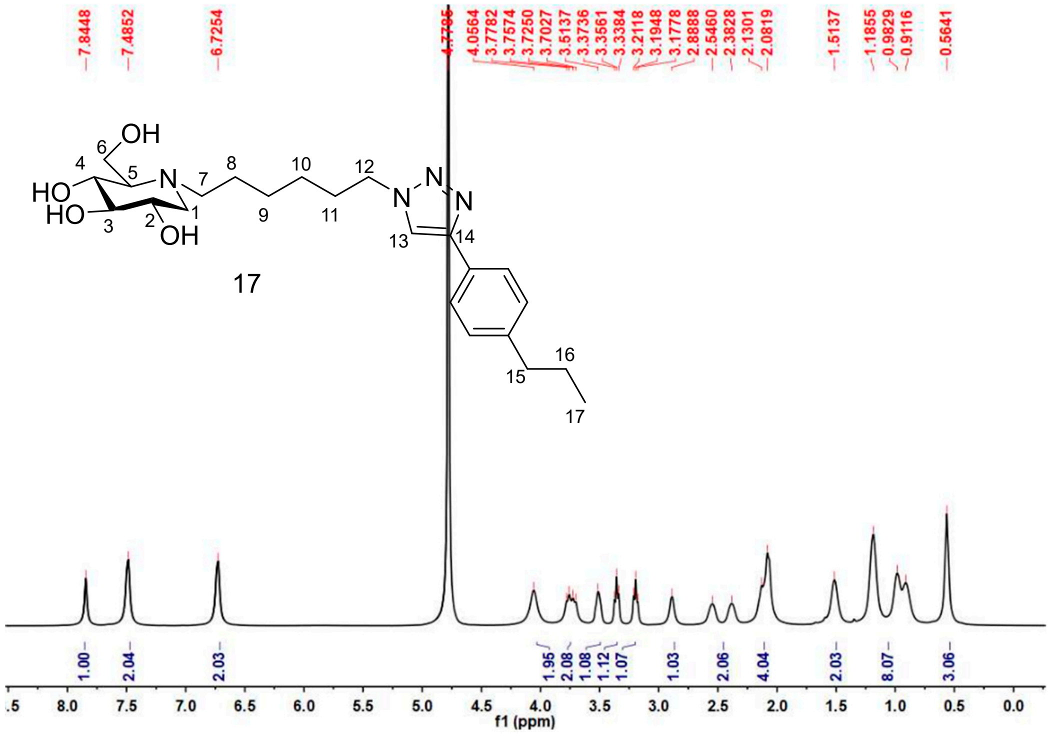

^{13}C NMR (125 MHz, D_2O) spectrum of **17**

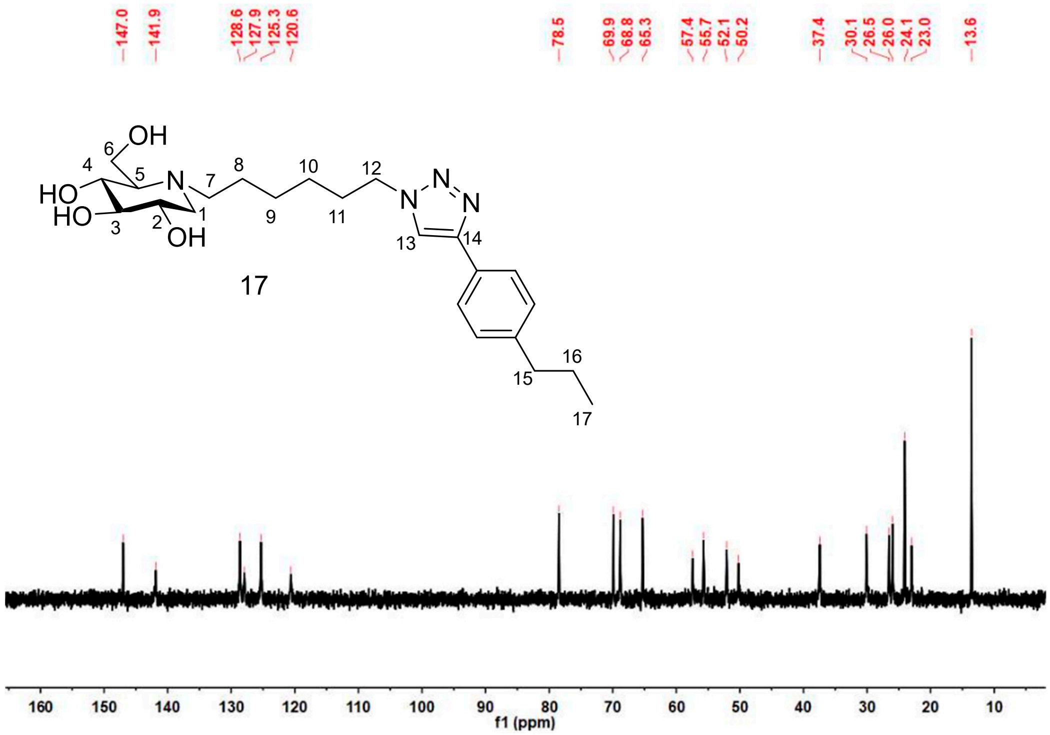

HRMS spectrum of **17**

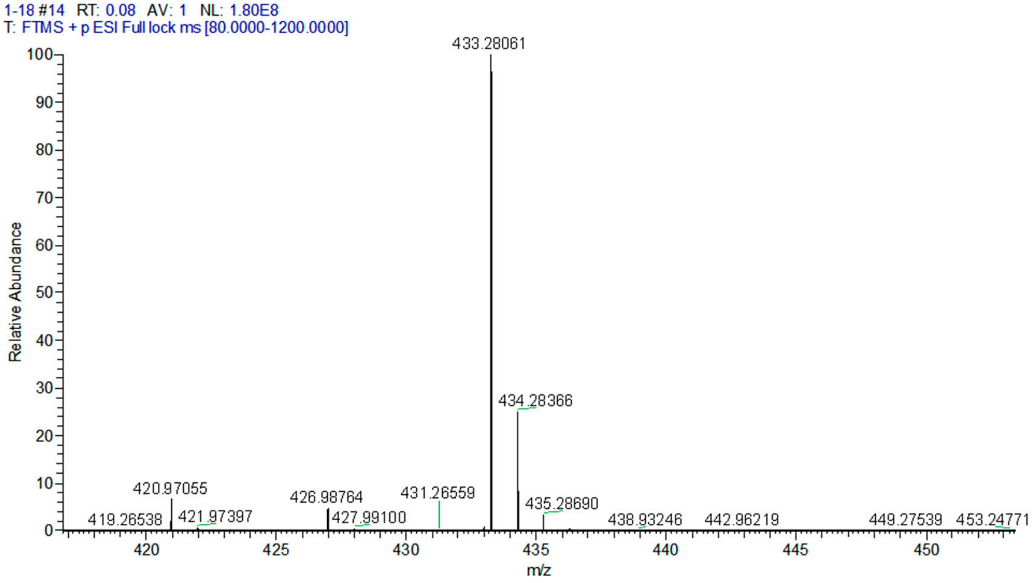

^{1}H NMR (500 MHz, D_2O) spectrum of **18**

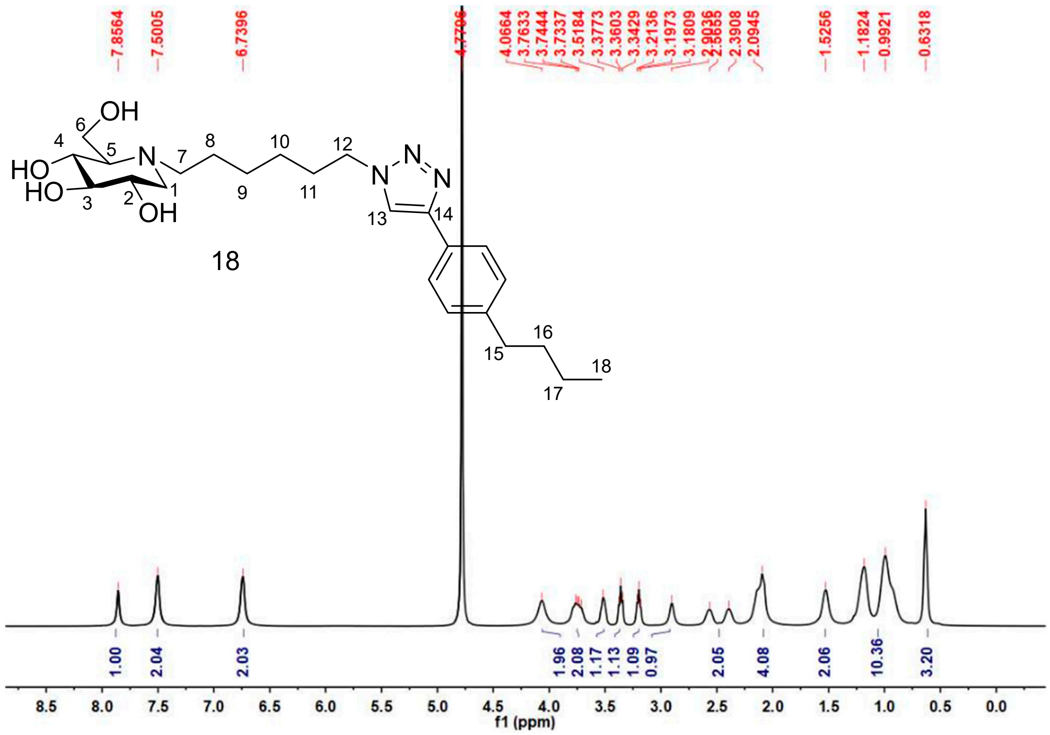

^{13}C NMR (125 MHz, D_2O) spectrum of **18**

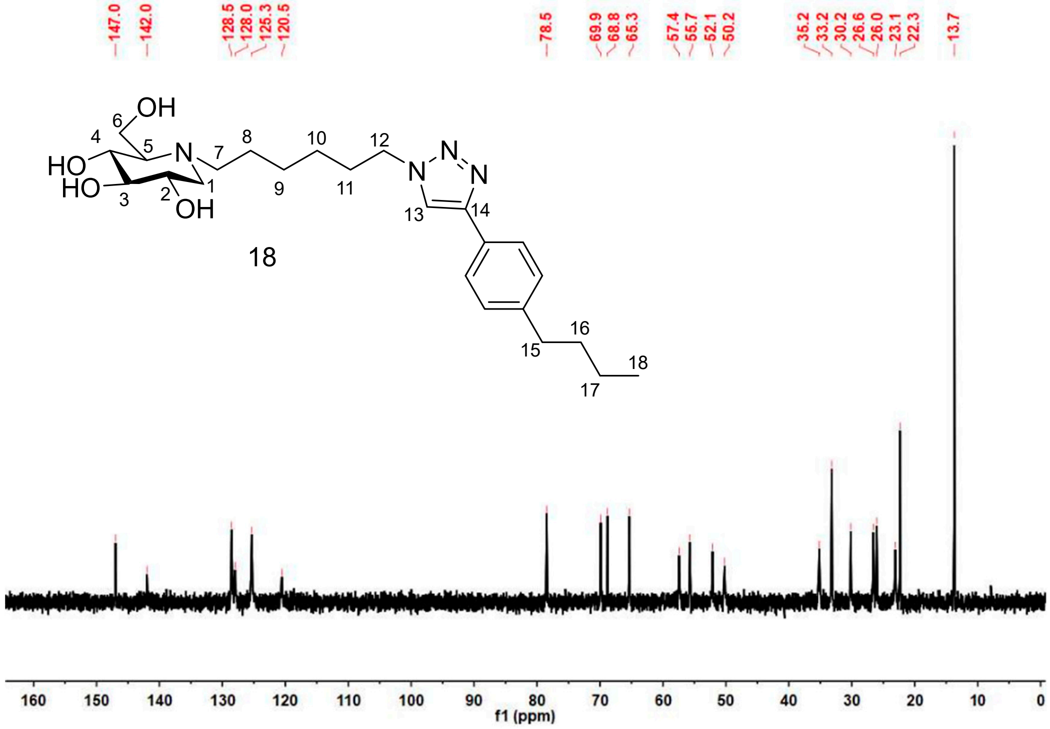

HRMS spectrum of **18**

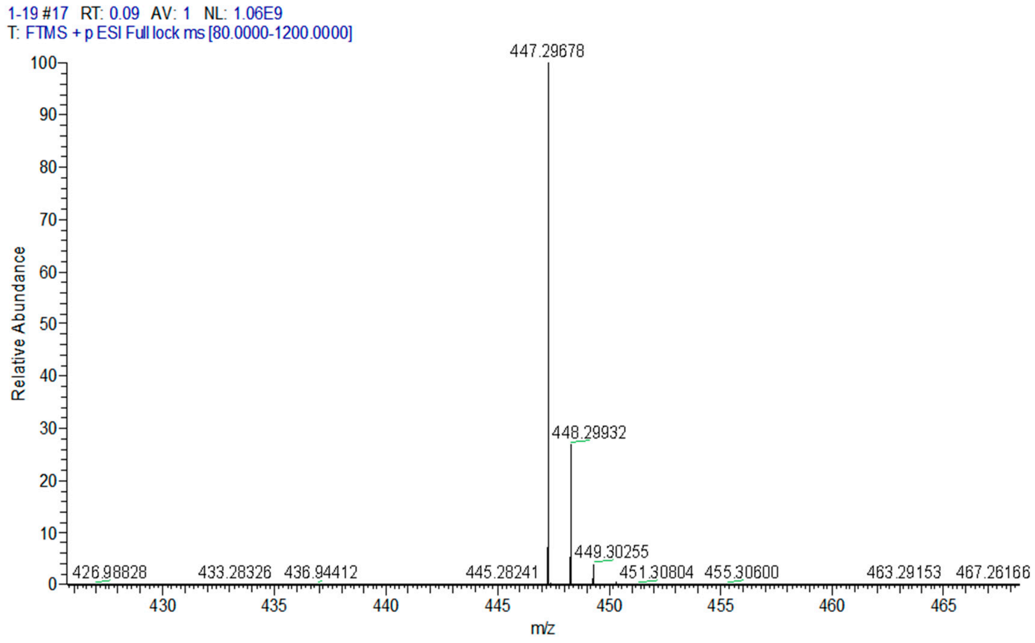

^{1}H NMR (500 MHz, D_2O) spectrum of **19**

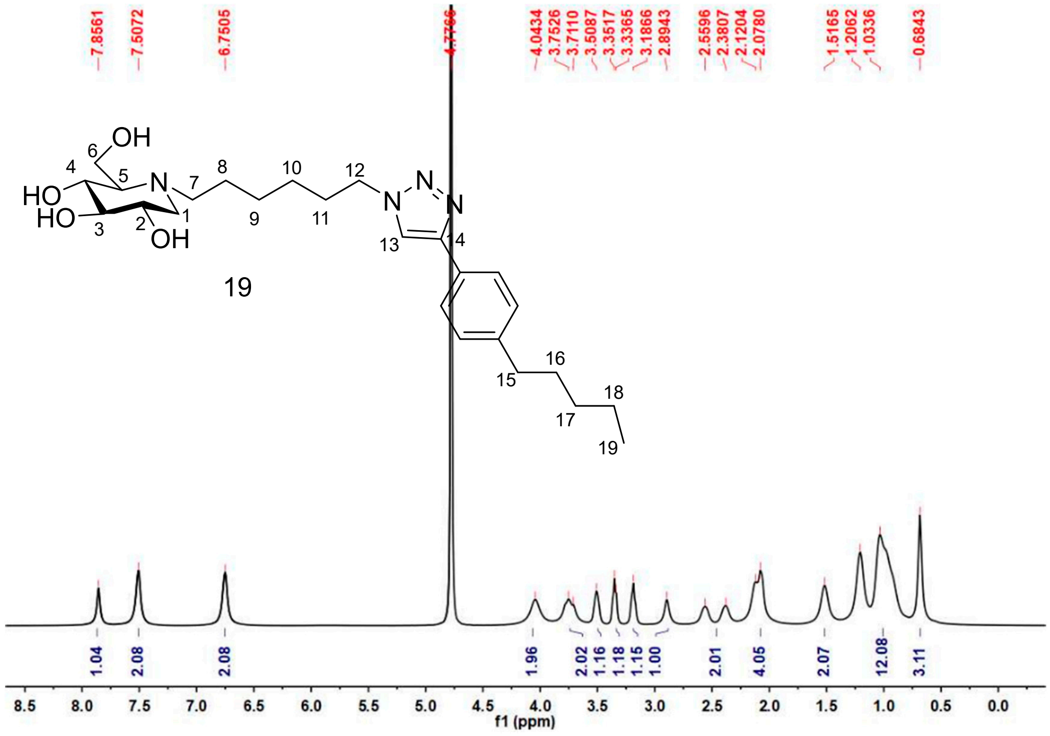

^{13}C NMR (125 MHz, D_2O) spectrum of **19**

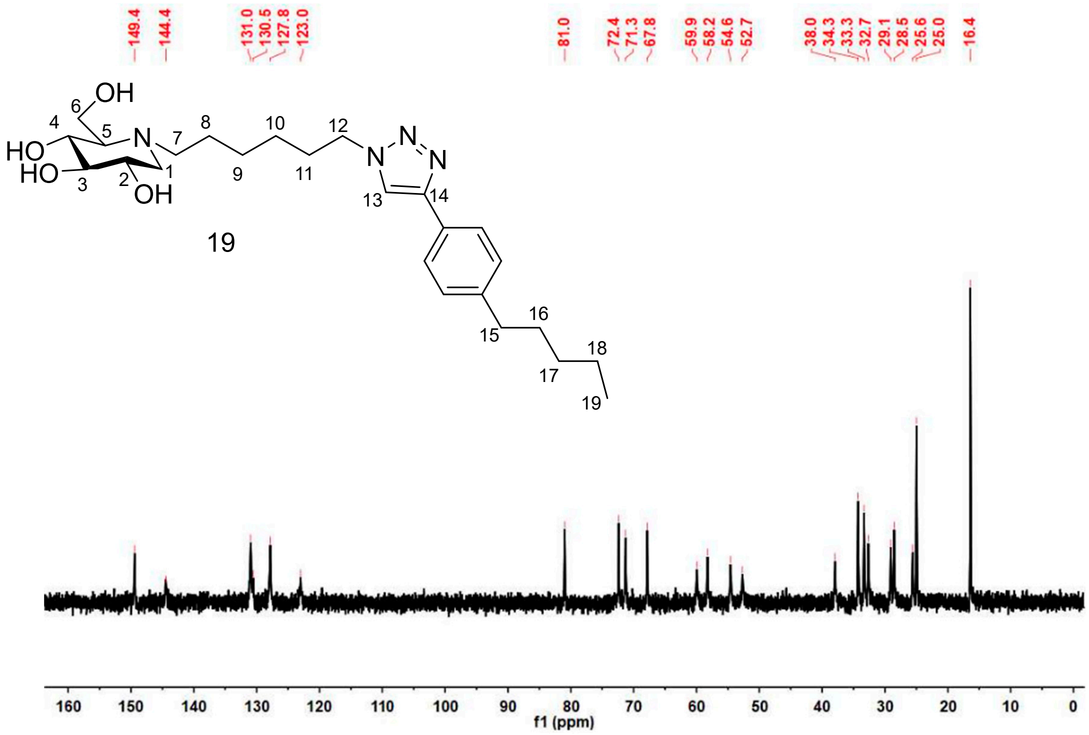

HRMS spectrum of **19**

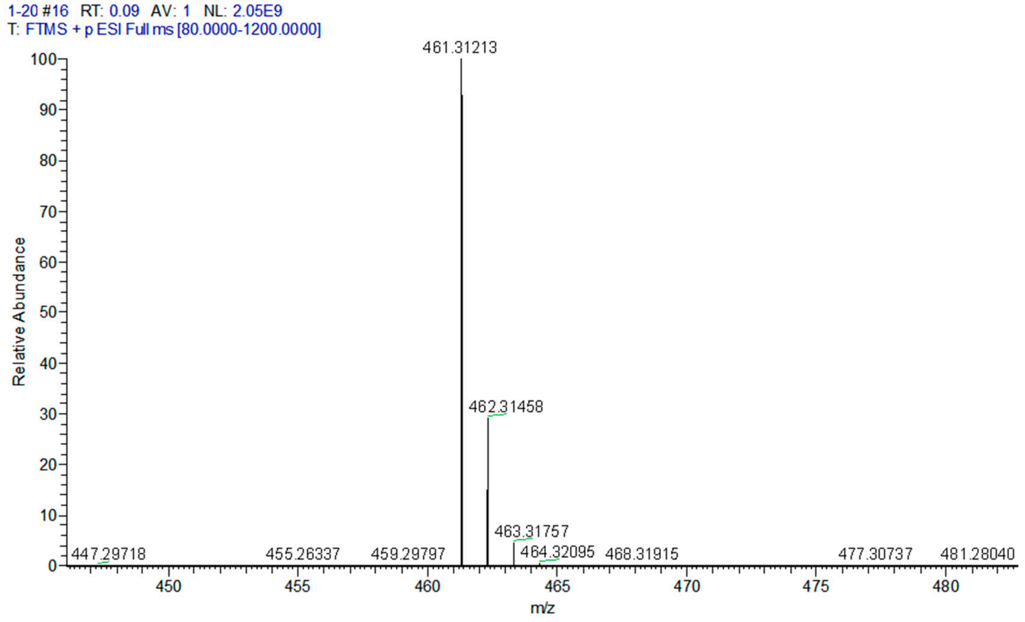

^{1}H NMR (500 MHz, D_2O) spectrum of **20**

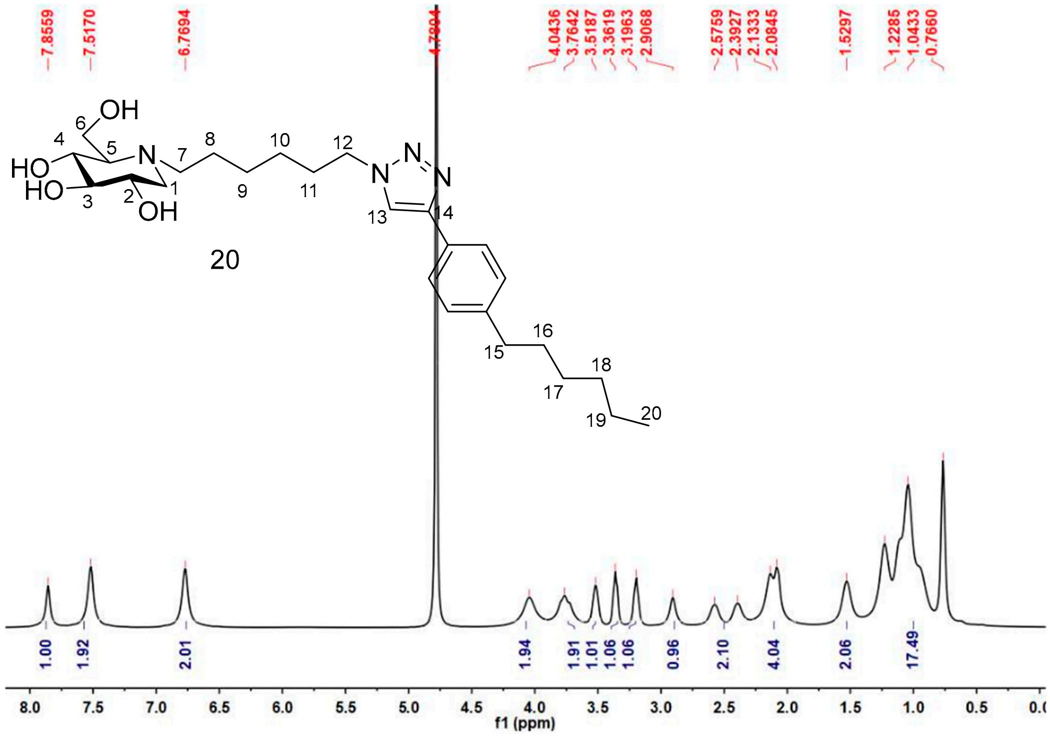

^{13}C NMR (125 MHz, D_2O) spectrum of **20**

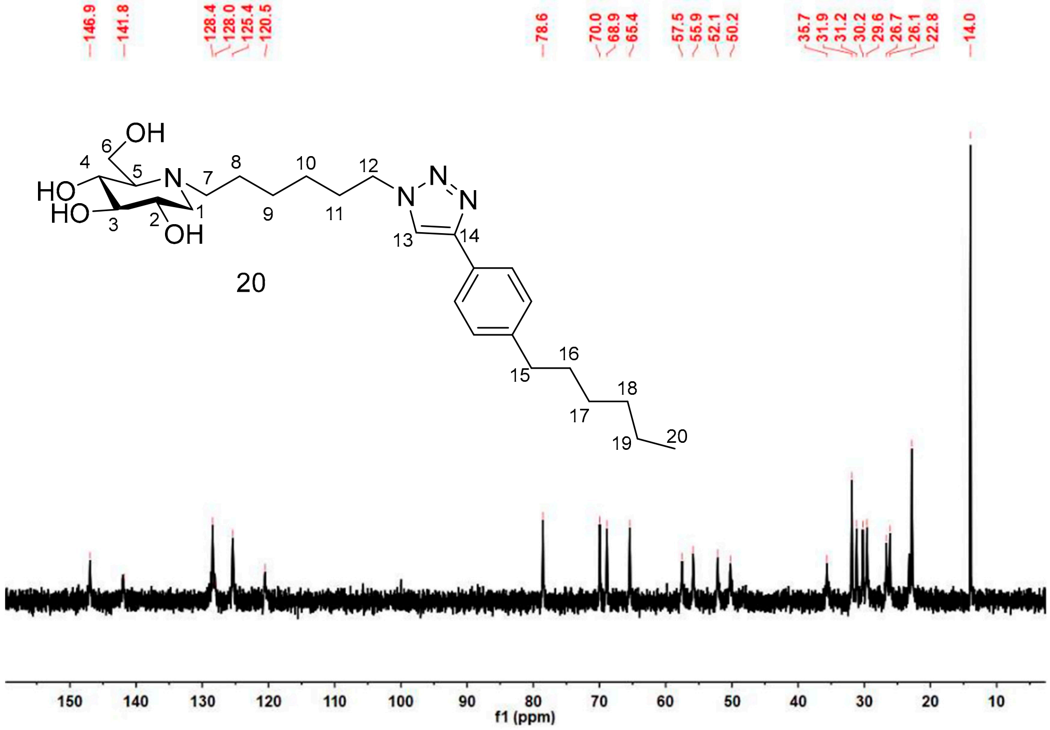

HRMS spectrum of **20**

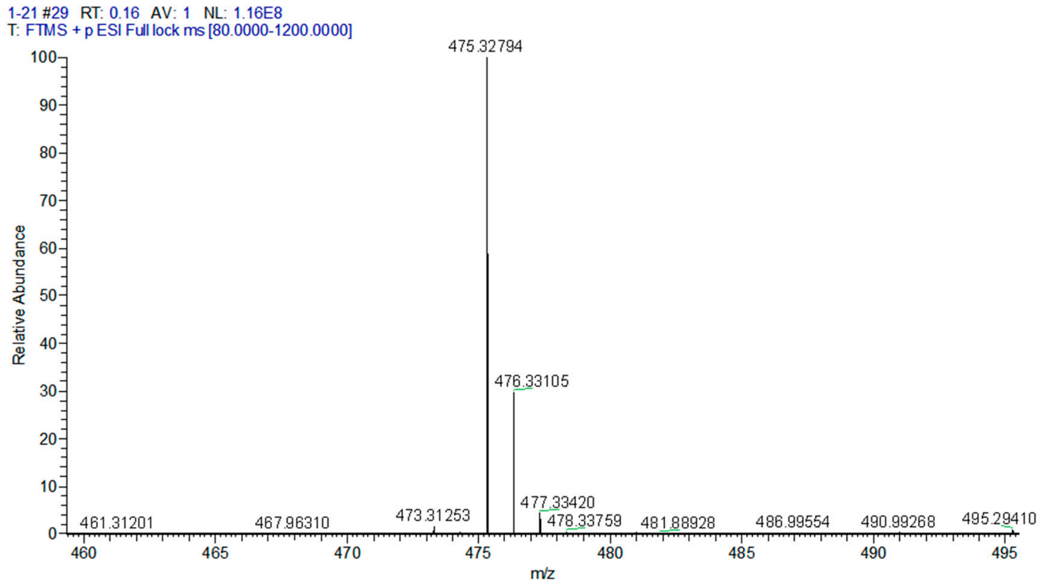
1-21 #29 RT: 0.16 AV: 1 NL: 1.16E8
T: FTMS + p ESI Full lock ms [80.0000-1200.0000]
475.32794
476.33105
477.33420
478.33759
473.31253
461.31201
467.96310
481.88928
486.99554
490.99268
495.29410
Relative Abundance
m/z

Homology modeling of Protein

The modeling result of MAL12 is depicted in Figure S1A and S1B. The Ramachandran plot for MAL12 showed that 99% residues are in allowed regions, indicating that the 3D structure of the model is reasonable. The structural analysis of the MAL12 modeling results is shown in Figure S1C. The MAL12 structure is basically consistent with the template structure. Both of them have the same alpha helix and beta strand regions. The overall identity of the amino acid sequence was 72.12%.

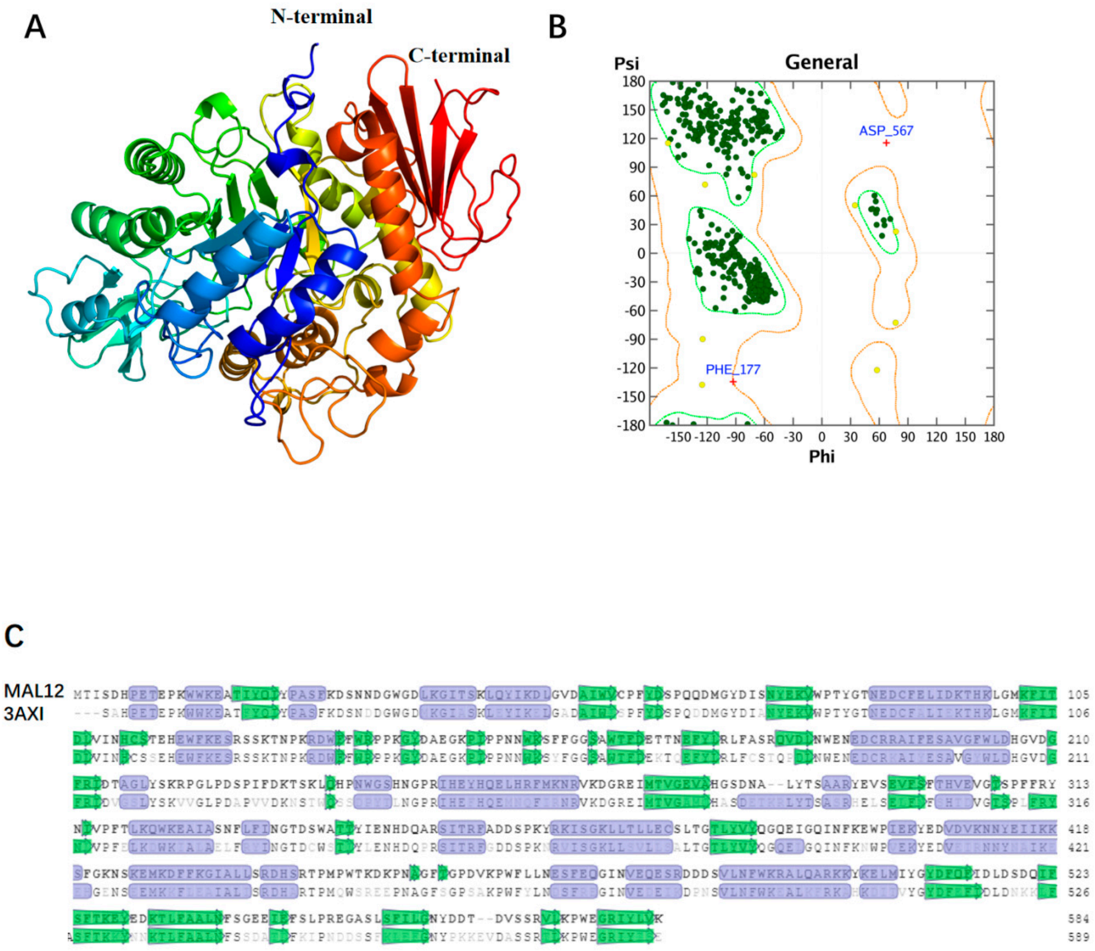

Figure S1. (A) The homology model of MAL12. (B) Ramachandran plot for MAL12. Dark green dots represent the residues in favored regions; yellow dots represent the residues in allowed regions, red cross represent the residues in irrational regions. (C) The structure-based sequence comparison between MAL12 and the template structure. The mismatched residues are displayed with fade, the sequence corresponding to alpha-helix and beta strand regions are marked with blue and green color.
